# Stopping the Chain: Isolated Molecular Intermediates of Anionic Styrene Polymerization

**DOI:** 10.1002/anie.202510397

**Published:** 2025-08-21

**Authors:** Annika Schmidt, Carsten Strohmann

**Affiliations:** ^1^ Inorganic Chemistry TU Dortmund University Otto‐Hahn‐Straße 6 44227 Dortmund Germany

**Keywords:** Anionic polymerization, Lithium alkyl chemistry, Organometallic chemistry, Reactive intermediate, X‐ray diffraction

## Abstract

Anionic polymerization developed to one of the most used polymerization techniques both in industry and academia in the last 70 years. Due to the required high reactivity of the polymerization, the isolation of the organometallic intermediate in between initiation and propagation step has not been possible so far and its mechanism is still not fully understood. Additionally, deprotonations in benzylic positions remain challenging, which is a prerequisite for a successful living carbanionic polymerization, but also complicating the synthetic access to the intermediates. Herein, we present the synthesis and isolation of the first‐step carbolithiation product of anionic styrene polymerization in diethyl ether and tetrahydrofuran. Characterizations in solid‐state by X‐ray diffraction and in solution state by extended nuclear magnetic resonance (NMR) experiments were carried out. Differing initiation reactivity was observed by in situ fourier transform‐infrared (FT‐IR) spectroscopy, revealing the intermediates to be responsible for polymerization reactivity. A possible mechanistic explanation for the initiation state was presented by density functional theory (DFT) calculations. By our approach of isolating the chain‐relevant intermediates on a molecular level, we gained new insights on the existing mechanism.

## Graphical abstract

We present the organometallic first‐step carbolithiation products of anionic styrene polymerization with diethyl ether and thf, that have not been isolated so far due to the high reactivity of anionic polymerization. X‐ray solid‐state and NMR solution‐state experiments show different intermediates in the different solvents. In situ FT‐IR and DFT‐calculations present mechanistic proposals for the initiation reaction.

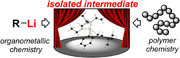

Plastics have become one of the most important materials in our industrialized world and are omnipresent in our everyday life. Out of the multiple techniques to synthesize polymers, anionic polymerization is one of the most precise methods due to its living character^[^ ^1^, ^2^ ^]^ and has paved the way for numerous complex polymer architectures^[^ ^3^, ^4^, ^5^, ^6^ ^]^ or beneficial polymer properties like those of thermoplastic elastomers (TPEs).^[^ ^7^, ^8^, ^9^ ^]^ However, due to the inherent high reactivity of the anionic polymerization process, the investigation of its mechanisms and the isolation of essential intermediates is challenging. Herein, we report the isolation of the first‐step intermediate of anionic styrene polymerization. By our organometallic method of isolating and investigating the key molecular intermediates, we aim to further elucidate the polymerization mechanism.

Carbolithiations are defined as the reactions of lithium organyls with double bonds. From their first studies by Ziegler et al. in 1928,^[^ ^10^, ^11^ ^]^ they developed to a useful tool in organic synthesis.^[^ ^12^, ^13^, ^14^ ^]^ One of their most used applications is the anionic polymerization (Scheme 1). In this reaction cascade, a lithium alkyl **A** adds to a carbon‐carbon double bond [e.g., styrene (**1**)] in an initiation step. This formed intermediate **B** is able to add to further double bond monomers in propagation steps that are repeated to form the polymer chain **C**.^[^ ^15^, ^16^ ^]^ In the 1950s, Szwarc et al. observed the “living” character of anionic polymerization, meaning that without electrophilic termination reagents, the polymer chain is able to “live” and propagate until consumption of (further added) monomers.^[^ ^17^, ^18^ ^]^ This characteristic of anionic polymerization explains the low polydispersity as well as the easy synthesis of block‐co‐polymers, like industrially relevant polystyrene block‐co‐polymers with polybutadiene.^[^ ^19^, ^20^, ^21^ ^]^

**Scheme 1:**
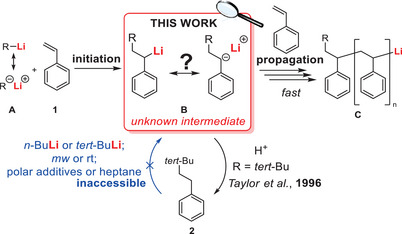
Schematic overview on the mechanism of anionic styrene polymerization (R = org. substituent). In this work, the structure of the so far unknown intermediate will be covered.

Even though the reaction is known for almost 100 years and carbanionic polymerizations are carried out in industry on a mega ton scale annually,^[^ ^22^ ^]^ the mechanism of anionic polymerization has not been clarified completely, inter alia as the intermediate between initiation and propagation has not been isolated. As the reaction of lithium alkyl reagents with vinylic monomers leads to polymerization products, which is clearly wished for the synthesis of a polymer, the halting of the chain‐growth is challenging.

Currently, mechanistic suggestions are based on studies from the 1960s and 1970s, that already showed an influence of the solvent on the reactivity.^[^ ^23^, ^24^, ^25^, ^26^, ^27^, ^28^, ^29^ ^]^ Waack et al. monitored the reaction of various lithium alkyls to 1,1‐diphenylethylene (DPE), which does not homopolymerize itself,^[^ ^30^, ^31^, ^32^, ^33^, ^34^ ^]^ in polar solvents and determined reactions orders regarding DPE and lithium alkyls.^[^ ^35^, ^36^, ^37^, ^38^ ^]^ It was found that lithium alkyls show reaction orders of fractions below 1 (e.g., 0.25 for *n*‐butyllithium in THF), which was attributed to aggregation effects and ion‐pair interactions. However, solid‐ and solution‐state structures of most lithium alkyls had not yet been reported at that time.

Nevertheless, knowledge of polymerization processes is needed today more than ever, that is, for the understanding of reactivities for selective polymer syntheses,^[^ ^39^, ^40^, ^41^, ^42^, ^43^, ^44^, ^45^, ^46^, ^47^, ^48^, ^49^ ^]^ insights into formation principles as basis for possible depolymerization approaches^[^ ^50^, ^51^, ^52^, ^53^, ^54^, ^55^ ^]^ and new (bio‐based) monomers, which often display more complicated structural features.^[^ ^56^, ^57^, ^58^, ^59^, ^60^, ^61^, ^62^, ^63^ ^]^

Herein, we report the isolation of the first‐step intermediate of anionic styrene polymerization in polar solvents.^[^ ^64^ ^]^ The intermediates with diethyl ether and THF were isolated and characterized in solid‐state by X‐ray diffraction and in solution by extended nuclear magnetic resonance (NMR) studies. The varying reactivity in the initiation step was shown with in situ fourier transform‐infrared (FT‐IR) spectroscopy and a mechanistic hypothesis was given by DFT‐calculations.

On our journey to aim the isolation of the intermediate of anionic styrene polymerization, we followed a procedure described by Taylor,^[^ ^65^, ^66^ ^]^ who brought the reaction cascade to a standstill after initiation. Analogously, we carried out the addition of *tert*‐BuLi to styrene in diethyl ether at –80 °C yielding only the monoaddition product **2** (Scheme 1, R = *tert*‐Bu). However, our approaches to relithiate the monoaddition product **2** at the benzyl position were unsuccessful with strong lithium bases like *tert*‐butyllithium and *n*‐butyllithium, neither under ambient conditions nor in the microwave, or with addition of polar additives (see SI; Scheme 1). The challenging deprotonation of benzylic carbanions^[^ ^67^, ^68^, ^69^, ^70^, ^71^, ^72^, ^73^ ^]^ (like those in the polymer chain) with lithium alkyls (like the active polymer chain) is one prerequisite for successful polymerization without termination steps.

Fortunately, by direct addition of *tert*‐butyllithium to styrene in diethyl ether without quenching of the lithiated addition product, we could isolate the first‐step carbometallation product as crystalline solid directly from the reaction solution at −80 °C (Scheme 2). The X‐ray structure obtained shows a monomeric carbanionic addition product of *tert*‐BuLi to styrene that coordinates the lithium center in a *η*_3_‐coordination of the formal carbanionic center and two neighboring carbon centers of the aromatic ring (i.e., *ipso*‐C and one *ortho‐C)*. This lithium center is further coordinated by two diethyl ether ligands.

**Scheme 2:**
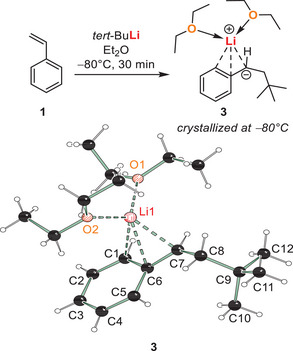
Solid‐state structure of addition product of *tert*‐butyllithium initiated styrene polymerization with diethyl ether. Only one molecule from the asymmetric unit is shown. Selected bond lengths [Å]: C7─Li1 2.1927(12); C6─Li1 2.3181(12); C1─Li1 2.4215(13); C6─C7 1.4063(8); O1–Li1 1.9431(12); O2─Li1 1.9345(12).^[^ ^74^ ^]^

The direct addition of *tert*‐butyllithium to styrene proved to be sensitive to the reaction conditions, as by performing the equivalent reaction in THF, contrary to diethyl ether, polymerization even at –80 °C was observed.^[^ ^35^, ^36^, ^37^, ^38^, ^65^, ^66^ ^]^ To isolate the first carbolithiation intermediate with different ligands, like coordinating THF (**4**), the ligand exchange reaction at –80 °C with intermediate **3** proved to be a successful approach (Figure 1). In **4**, the lithium center is coordinated by three THF molecules and the carbanionic atom C7 in a *η*_1_‐fashion. The C6─C7 bond distance is slightly elongated in comparison to **3** which suggests less stabilization of the carbanionic charge through delocalization in the *π*‐system of the phenyl ring, in accordance to the lithium position in **4**. Also, the Li1─C7 bond distance is elongated by 0.04 Å in comparison to **3**, which may be attributed to a smaller proportion of the charge balance through the carbanionic center in **4** than in **3**.

**Figure 1:**
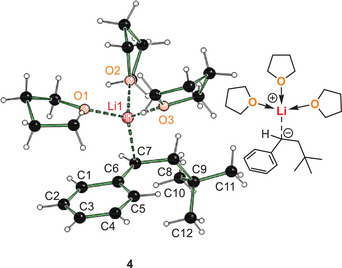
Solid‐state structure of addition product of *tert*‐butyllithium initiated styrene polymerization with THF. Selected bond lengths [Å]: C7─Li1 2.2363(18); C6─C7 1.4116(11); O1─Li1 1.9493(17); O2─Li1 1.9581(18); O3─Li1 1.9754(18).^[^ ^74^ ^]^

To confirm the solid‐state structures in solution, extended NMR experiments were performed. Figure 2 shows an overview of the experiments performed for **3** (for further information and data for **4**, see SI). NMR experiments of the reactive intermediate **3** were feasible even at room temperature as the ^1^H‐NMR spectrum (Figure 2a) shows. The integral ratio of carbanionic compound protons to etheric protons is ∼1:2, confirming the stoichiometry of the solid‐state structure. The ^1^H‐DOSY and the ^7^Li‐DOSY experiment (Figure 2c) showed similar diffusion coefficients of 5.27 ± 0.02 Å (for ^1^H) for the carbanionic and 5.55 ± 0.01 Å (for ^7^Li) for the lithium‐containing part. Therefore, the lithium center and the carbanionic substituent must be in the same aggregate. Together with the found contacts in the ^1^H‐^7^Li‐HOESY NMR experiment (Figure 2d; lithium center contact to *ortho*‐aromatic proton, to the proton of the carbanionic center and to the adjacent C*H*_2_‐protons), this proves the existence of a contact ion pair in solution, like structure **3**.

**Figure 2:**
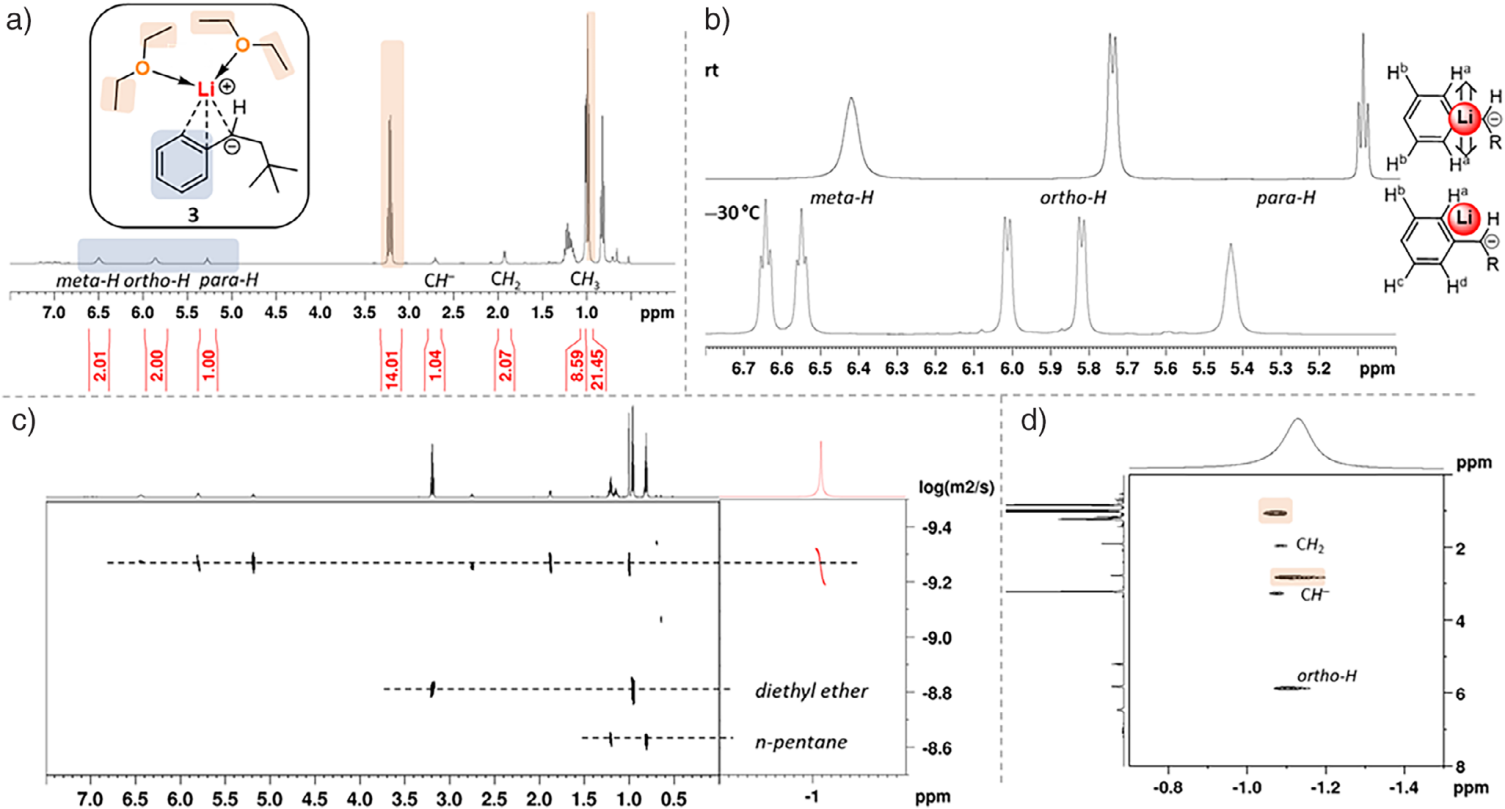
NMR studies performed on **3**. a) ^1^H‐NMR spectrum with assignment of peaks. b) Excerpt of ^1^H‐NMR spectrum at rt and –30 °C for aromatic protons of **3**. c) Comparison of ^1^H‐DOSY and ^7^Li‐DOSY (red). d) ^1^H‐^7^Li‐HOESY with assignment of peaks.

By cooling the sample to lower temperatures of −30 °C (Figure 2b), the signals of aromatic protons were split up. We explain this apparent desymmetrization of the aromatic ring with the *η*_3_‐coordination of the carbanion. At lower temperatures, the coordination “freezes” at one side of the aromatic ring, like in structure **3**, leading to chemically inequivalent protons. Analog experiments were performed for solid‐state structure **4** (see SI). Most features of the NMR experiments remain comparable to **3** (i.e., chemical shifts, aggregate size from DOSY‐experiment, etc.). The ^13^C chemical shift of the carbanionic carbon, however, is 53.9 ppm for **4** and 59.5 ppm for **3**, indicating a higher electron density at the carbanionic center in **4** than in **3**, which is in accordance with the bond distances from the solid‐state structures. Furthermore, the ^1^H‐^7^Li‐HOESY shows a lower relative contact‐integral between the lithium cation and the protons of the carbanion, which also agrees with the elongated Li─C distances for **4** in the solid‐state structures.^[^ ^75^ ^]^

As the monoaddition reaction of *tert*‐butyllithium to styrene showed a high dependency on the solvent and was not feasible in THF, we were interested in the underlying processes. The observed polymerization in THF may be explained either with a low initiation rate or a fast propagation of intermediate **4**. As we found a way to stop the polymerization cascade and were able to isolate the intermediates, we could also investigate the initiation step separately from propagation. Therefore, the addition reaction of *tert*‐butyllithium to styrene in diethyl ether was followed in situ by FT‐IR spectroscopy. Scheme 3 displays the temporal course of IR frequency bands 2666 and 1589 cm^−1^. The band of 2666 cm^−1^ can be attributed to a C─H vibration from *tert*‐butyllithium, that is found in a similar range for related lithium alkyls.^[^ ^76^ ^]^ Upon addition of styrene (at 100 seconds), the *tert*‐butyllithium band decreases while an increase in an IR band at 1589 cm^−1^ is observed. After reaction, the monoaddition product **2** was the only product, identified by GC/MS. Therefore, this band must be assigned to product **3**, likely a C═C bond vibration. The reaction proceeds fast even at −78 °C in a cooling bath (∼−60 °C inside the flask) with a half‐life of 60 s and a full conversion after ∼400 s.

**Scheme 3:**
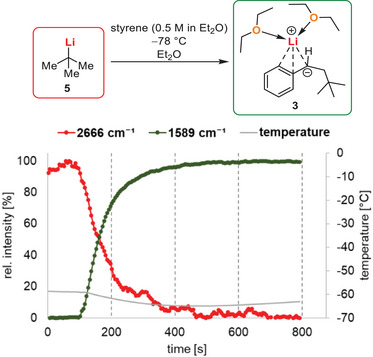
Plot of the normalized intensities of IR bands 2666 and 1589 cm^1^ against time for in situ FT‐IR experiment of addition of *tert*‐butyllithium to styrene in diethyl ether at –78 °C.

The observed fast polymerization in THF prevents the in situ FT‐IR investigation of the initiation mechanism in THF. Therefore, the model substrate DPE was used (see SI, Figure S8).^[^ ^35^, ^36^, ^37^, ^38^ ^]^ In THF, the addition of *tert*‐butyllithium to 1,1‐diphenylethylene at –80 °C is even faster, as the half‐life is only 10 seconds, and a full conversion is reached after 120 s. Especially, after conducting the reaction with the same substrate (1,1‐diphenylethylene) in diethyl ether (see SI, Figure S11, half‐life ∼5 min), the velocity difference of the reactions in dependency of the used solvents becomes apparent. Consequently, the observed polymerization in THF at low temperatures cannot be explained with a low initiation rate. This is in accordance with studies that reported the low polydispersity of polystyrenes formed in THF due to a fast initiation reaction.^[^ ^77^, ^78^, ^79^, ^80^ ^]^ The isolated intermediates **3** and **4** must play an important role in the propagation step being accountable for the solvent‐dependent reactivity.

To support our experimental findings of a fast initiation step in THF theoretically, quantum‐chemical DFT‐calculations were performed. *tert*‐Butyllithium was modelled as monomer and dimer, in accordance with the aggregation in solution (monomer in THF, dimer in diethyl ether).^[^ ^81^, ^82^, ^83^, ^84^ ^]^ M062‐X method and 6–311+G(d) as basis set were used.^[^ ^85^, ^86^ ^]^ Further conditions (temperature dependency, ligands, pcm‐models) were considered and described in the SI (see Table S3).

For the dimeric system, **TS‐d** was found with an activation barrier of 41 kJ·mol^−1^ (Figure 3), which is in accordance with the relatively fast reaction at low temperatures.

**Figure 3:**
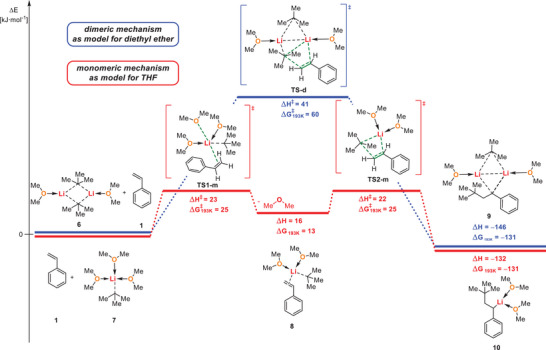
Mechanistic proposals of addition of *tert*‐butyllithium to styrene for a dimeric and monomeric system with dimethyl ether ligands with calculated transition states. The activation barrier energies ΔH^≠^ and ΔG^≠^_193 K_ and relative energy barriers ΔH and ΔG_193K_ were referenced to the educts **1/6** and **1/7**. Compounds **9** and **10** are the isodesmic products of the reaction; to form intermediates **3** and **4** further steps follow. The relevant center of the transition state is marked in green. [M062X, 6–311+G(d), T = 193 K].

The instantaneous reaction in THF however, suggests a transition state that differs from the one in diethyl ether. Also, we were not able to find a direct transition state of monomeric *tert*‐butyllithium (**7**) to styrene (**1**) due to the necessity of a five‐coordinated lithium cation (3 dimethyl ether, *tert*‐butyl, styrene/to‐be carbanion) in this scenario, which is very unlikely to be formed, especially without chelating ligands.^[^ ^87^ ^]^ Different scenarios were considered (see SI) of which a pre‐coordination of styrene appears to be the most realistic one. Herein, one etheric ligand is exchanged to *π*‐coordinating styrene with an energetic barrier of 23 kJ·mol^−1^ (**TS1‐m**). The pre‐coordination product **8** is destabilized by 16 kJ·mol^−1^ and can form the addition product with an additional activation barrier of 6 kJ·mol^−1^ (**TS2‐m**). The low reaction barriers are in accordance with the instantaneous reaction in THF. Also, it was already proposed that the rate determining step might involve coordination of a C−C double bond to the lithium alkyl aggregate, however this was for hydrocarbon solvents.^[^ ^30^, ^31^, ^88^, ^89^ ^]^

In conclusion, by applying organometallic methods to anionic polymerization, we were able to isolate the first‐step carbolithiation product of anionic styrene polymerization in polar solvents. Thereby, new insights into the long‐existing, reactive mechanism of anionic polymerization were obtained. As the deprotonation of the addition product was not accessible with lithium alkyl bases (which is one major prerequisite for a living anionic polymerization), the addition reaction of *tert*‐butyllithium to styrene in diethyl ether was carried out, which led to isolated monoaddition products with diethyl ether and THF. X‐ray diffraction studies revealed the molecular structure which was verified in solution by extended NMR experiments. While the intermediate with diethyl ether (**3**) is characterized by a *η*_3_‐coordination of the carbanion to the lithium center, the intermediate with THF (**4**) shows less stabilization of the carbanionic center by the aromatic ring. With the isolated intermediates in hand, it was possible to investigate the initiation step separately from propagation. Next to the observation, that the direct synthesis of the addition product was only viable in diethyl ether, in situ FT‐IR studies revealed a faster initiation reactivity in THF. Thereby, the isolated intermediates must be responsible for the differing reactivities in both etheric solvents. DFT‐calculations helped proposing a possible mechanistical hypothesis. After the separate investigation of this first initiation step of anionic polymerization, we already found in follow‐up experiments that compounds **3** and **4** can perform further carbolithiation steps. In future research, we aim to use the isolated intermediates for a separate investigation of the propagation step as a model for the active polymer chain.

## Supporting Information

The authors have cited additional references within the Supporting Information.^[^ ^90^, ^91^, ^92^, ^93^, ^94^, ^95^, ^96^, ^97^, ^98^, ^99^ ^]^

## Conflict of Interests

The authors declare no conflict of interest.

## Supporting information



Supporting Information

Supporting Information

## Data Availability

The data that support the findings of this study are available from the corresponding author upon reasonable request.
