# Supplementary material for: Stopping the Chain: Isolated Molecular Intermediates of Anionic Styrene Polymerization
Source: Angew Chem Int Ed Engl. 2025 Aug 21;64(42):e202510397. doi: 10.1002/anie.202510397 (PMC12518692; doi:10.1002/anie.202510397)
Supplement: Supplementary file 1 — Supporting Information [file ANIE-64-e202510397-s002.pdf]

Supporting Information
©Wiley-VCH 2021
69451 Weinheim, Germany

Stopping the Chain: Isolated Molecular Intermediates of Anionic Styrene Polymerization

Annika Schmidt, and Carsten Strohmann*

Abstract: Anionic polymerization developed to one of the most used polymerization techniques with a high control on the polymeric features. Despite the huge industrial and academic success of anionic polymerization in the last 70 years, its mechanism still is not fully understood. Due to the required high reactivity of the polymerization, neither the isolation nor the characterization of the organometallic intermediate in between initiation and propagation step has been possible so far. Additionally, deprotonations in benzylic positions remain challenging, which is, a prerequisite for a successful living carbanionic polymerization, but also further complicating the synthetic access to the relevant intermediates. Herein, we present the synthesis and isolation of the first-step carbolithiation product of anionic styrene polymerization in diethyl ether and tetrahydrofuran. Characterizations in solid-state by X-ray diffraction and in solution state by extended NMR experiments were carried out. Differing initiation reactivity was observed by *in situ* FT-IR spectroscopy and a possible mechanistic explanation was developed by DFT calculations. By our approach of isolating the chain-relevant intermediates on a molecular level, we gained new insights on the existing mechanism.

Table of Contents

Experimental Procedures **1**
General remarks 1
Synthesis 2
In situ FTIR studies 6
Results and Discussion **11**
Single crystal X-ray diffraction studies 11
NMR studies 13
Quantum chemical calculations 24
References **73**

Experimental Procedures

General remarks

All chemical syntheses with air-sensitive reagents (e.g. lithium alkyls) were carried out in dried, oxygen-free solvents under an inert gas atmosphere of argon (Argon 5.0 from *Messer Griesheim GmbH*). The standard glass apparatus used was baked out in an evacuated state (10^{-2} bar) before working with oxygen- or moisture-sensitive compounds. The solvents used were dried, purified, distilled and stored under argon atmosphere according to standard procedures. All reagents used, unless otherwise stated, were hand-delivered products without further purification. The solutions of the organometallic reagents used were products of *Sigma Aldrich GmbH* and *Acros Organics*. The deuterated solvents used for the NMR spectroscopy were products of *Eurisotop* and *Deutero*. All other reagents were products of the companies *Chemetall GmbH*, *Sigma Aldrich GmbH*, *FLUKA*, *Alfa Aesar*, *ABCR* and *Acros Organics*.

NMR spectra were recorded on an Avance III HD spectrometer from *Bruker AV* at a temperature of about 25 °C or –30 °C. The chemical shift data in ppm refer to the *δ*-scale. Spin-spin coupling constants (*J*) were given in hertz (Hz) ($^{n}J_{XY}$: coupling of observed nucleus X with a n bond distant nucleus Y). The following abbreviations were used to reflect multiplicities and signal shape: s = singlet, d = doublet, q = quartet, m = multiplet. ^{1}H-NMR spectra: Locking substances (internal standard): toluene (δ = 2.08, CD_2H). The number of hydrogen atoms per signal was determined by comparing the relative signal intensities. ^{13}C-NMR spectra: Lock substances (internal standard): toluene (δ = 137.48, C_6D_5H). All ^{13}C-NMR spectra were recorded ^{1}H-broadband decoupled ({^{1}H}). The signal assignments of the ^{13}C NMR spectra were partially supported by 2D-NMR experiments, the results of which are included in the corresponding signal assignments.

For gaschromatography/mass spectroscopy (GC/EI-MS coupling) the 7890B GC system, an HP-5 MS capillary column (length 30 m, ID 0.25 mm) and the Qualitative Analysis 10.0 software from Agilent Technologies were used. Detector: Mass Selective Detector 5977A from Agilent Technologies (EI(+)MS, 70 eV). Method: 50 °C (0.5 min), then 20 °C/min to 290 °C (2 min).The m/z values of the molecular ions and the selected fragments indicated are based in each case on the mass numbers of the isotopes with the greatest natural relative abundance.

The *ReactIR 700* from *Mettler Toledo* was used. The probe, equipped with a *DiComp* (diamond-composite) sample head, is coupled to an AgX 6 mm x 1.5 m glass fiber (silver halide). The measurement was carried out in a wavenumber range from 3000 cm^{-1} to 650 cm^{-1} with a resolution of 4 wavenumbers. The scan option was set to autoselect in each case. The digital evaluation was carried out using the device's own *iC IR 7.1* software.

Suitable crystals of compounds **3** and **4** were covered with an inert oil (perfluoroalkylether) at –80 °C using the *X-TEMP 2*[1] device in combination with a *SMZ1279* stereomicroscope from *Nikon Metrology GmbH* and mounted on a *MicroMount* from *MiTeGen*. Crystal structure determination was accomplished on a *Bruker D8 Venture* four-circle diffractometer using a *PHOTON II CPAD* detector by *Bruker AXS GmbH*. X-ray radiation was generated by microfocus source IμS Mo (λ = 0.71073 Å) by *Incoatec GmbH* with HELIOS mirror optics and a single hole collimator by *Bruker AXS GmbH*. For the data collection, the program APEX4 Suite (v2021.10-0)[2] with the integrated programs SAINT (integration) and SADABS (adsorption correction) by *Bruker AXS GmbH* were used. The processing and finalization of the crystal structure was done with the program Olex2.[3] The crystal structure was solved with the ShelXT[4] structure solution program using Intrinsic Phasing and refined with the ShelXL refinement package using Least Squares minimization.[5] The non-hydrogen atoms were refined anisotropically. U_{eq} is defined as one third of the trace of the orthogonalized tensor U_{ij}. For the hydrogen atoms the standard values of the SHELXL program were used with U_{iso}(H) = –1.2 U_{eq}(C) for CH_2 and CH and with U_{iso}(H) = –1.5 U_{eq}(C) for CH_3.

The quantum chemical calculations were carried out using the following programs: The molecules, if they could not be transferred from molecular structures in the solid state, were modeled with the inferface *GaussView 6.0*[6] and a Gaussian Job File (gjf, input files) was created. The calculations were carried out with the programs *Gaussian 16.*[7] All basic state structures were initially optimized without symmetry restrictions. A subsequent frequency calculation did not provide any imaginary frequencies for the minimum structures. The visualization of the energy-optimized structures was carried out with the program *Molekel 4.32*.[8]

Synthesis

Synthesis of protonated addition product of *tert*-butyl lithium and styrene 2

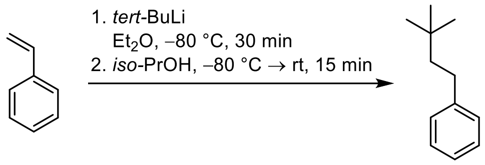

1 C_8H_8 104.06 g/mol — **2** $C_{12}H_{18}$ 162.14 g/mol

Following the procedure of Taylor *et al.*,[9] styrene (1.0 mmol, 104 mg, 1.0 eq.) was dissolved in an evacuated Schlenk flask in diethyl ether (1.00 mL). The reaction solution was cooled to –80 °C by an *iso*-propanol cooling bath. At this temperature *tert*-butyl lithium (1.9 M in pentane, 1.0 mmol, 0.52 mL, 1.0 eq.) was added and the reaction solution stirred for 30 minutes at –80 °C. *Iso*-propanol was added for quenching and warmed to room temperature. Water (5 mL) was added and the mixture was extracted with diethyl ether (3 x 5 mL), the organic phases were collected, dried with $MgSO_4$ and the organic solvents were evaporated rotary evaporation. **2** was obtained as a colorless oil (117 mg, 0.72 mmol, 72 %).

^{1}H-NMR: (500 MHz, C_6D_6): δ = 0.97 (s, 9H; C*H*$_3$), 1.52 (m, 2H; C*H*$_2$-CH$_2$-Ph), 2.56 (m, 2H; CH$_2$-C*H*$_2$-Ph), 7.17 (m, 3H; *H*$_{Ar}$), 7.27 (m, 2H; *H*$_{Ar}$) ppm.
^{13}C{^{1}H}-NMR: (500 MHz, C_6D_6): δ = 29.4 (s, 3C; *C*H$_3$), 30.5 (s, 1C; *C*CH$_3$), 31.7 (s, 1C; *C*H$_2$-CH$_2$-Ph), 46.7 (s, 1C; CH$_2$-*C*H$_2$-Ph), 125.9 (s, 1C; *C*$_{Ar}$), 128.6 (s, 2C; *C*$_{Ar}$), 128.7 (s, 2C; *C*$_{Ar}$), 143.6 (s, 1C; *C*$_{Ar}$) ppm.
GC/MS: (70 eV, t_R = 6.249 min): m/z = 162 [(M–H)$^+$], 105 [(M–C_4H_9)$^+$], 91 [(PhCH$_2$)$^+$], 77 [(Ph)$^+$], 57 [(Me$_3$C)$^+$].

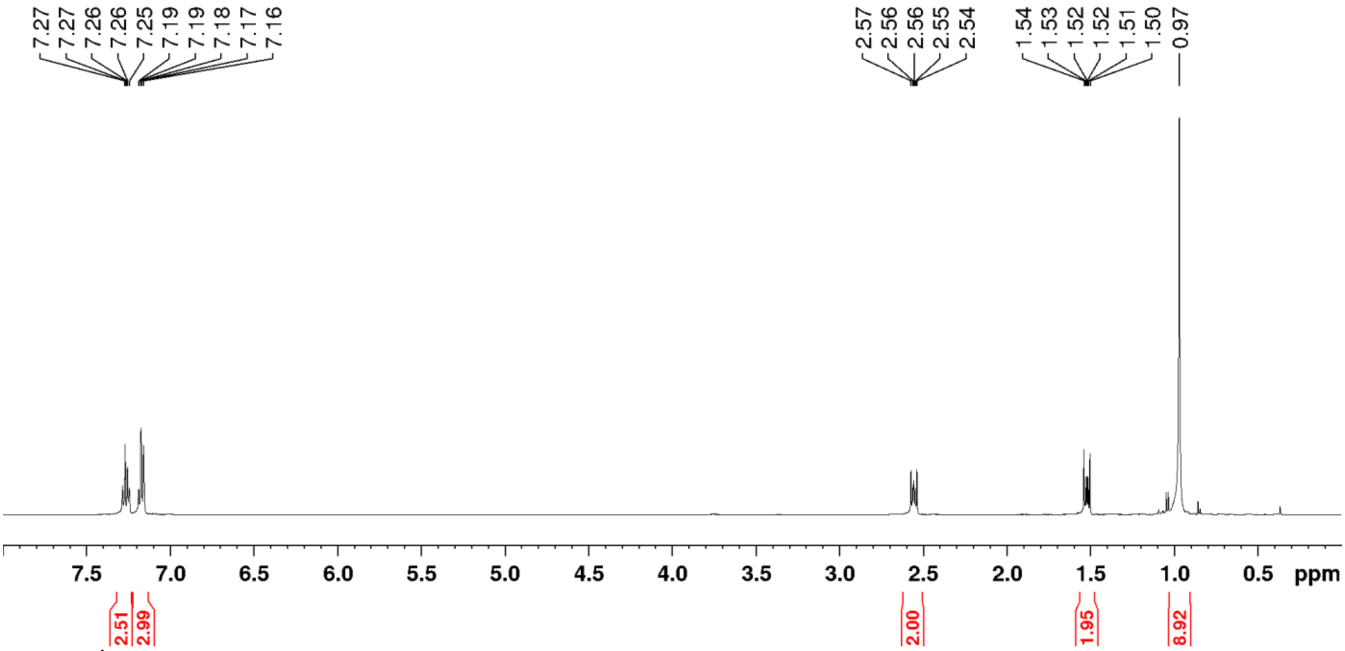

Figure S1: ^{1}H-NMR spectrum of **2.**

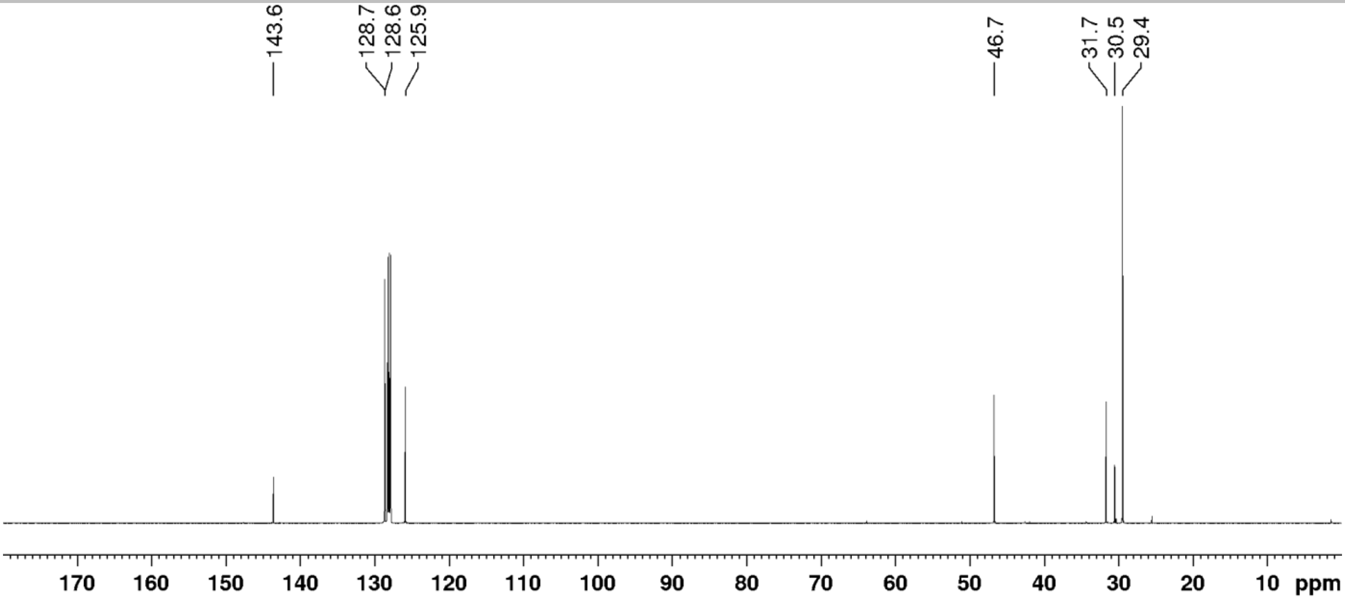

Figure S2: ^{13}C-NMR spectrum of **2**.

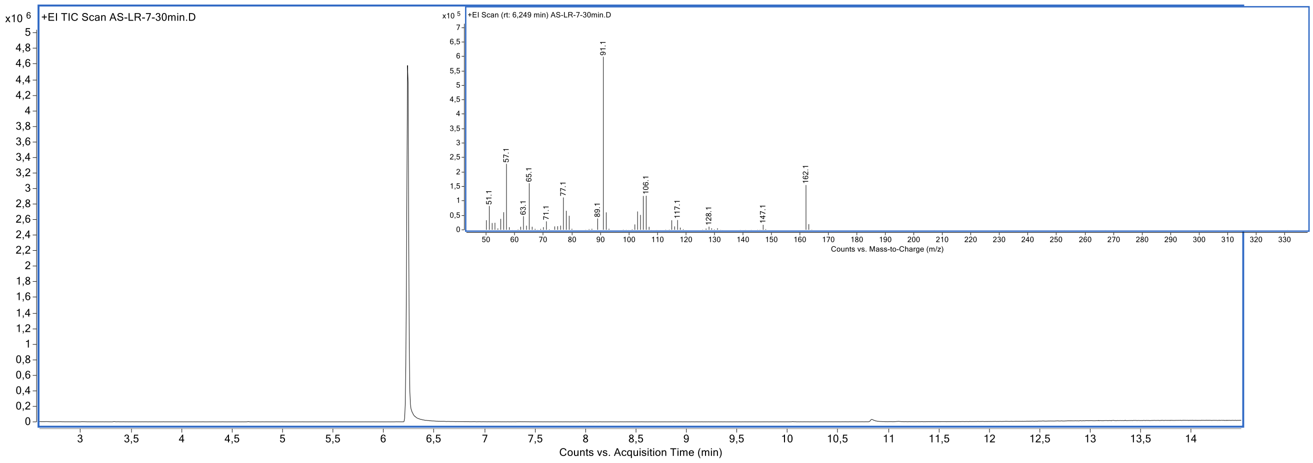

Figure S3: GC/MS chromatogram and mass spectra of **2**.

Deprotonation of 2
Variant A:

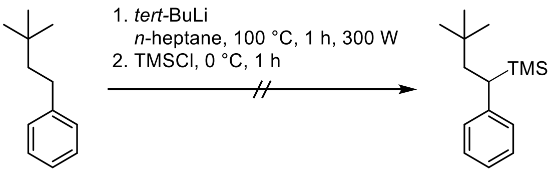

2
$C_{12}H_{18}$
162.14 g/mol

11
$C_{15}H_{26}Si$
234.18 g/mol

2 (162 mg, 1.0 mmol, 1.0 eq.) was added to a pre-heated microwave vial in an argon atmosphere. *n*-Heptane (1.00 mL) and *tert*-butyllithium (0.52 mL, 1.9 M in pentane, 1.0 mmol, 1.0 eq.) were added. The vial was capped and heated for 1 h at 100 °C with 300 W in a laboratory microwave. The reaction solution turned orange, however GC/MS analysis after trapping with TMSCl (108 mg, 1.0 mmol, 1.0 eq.) revealed no product formation.

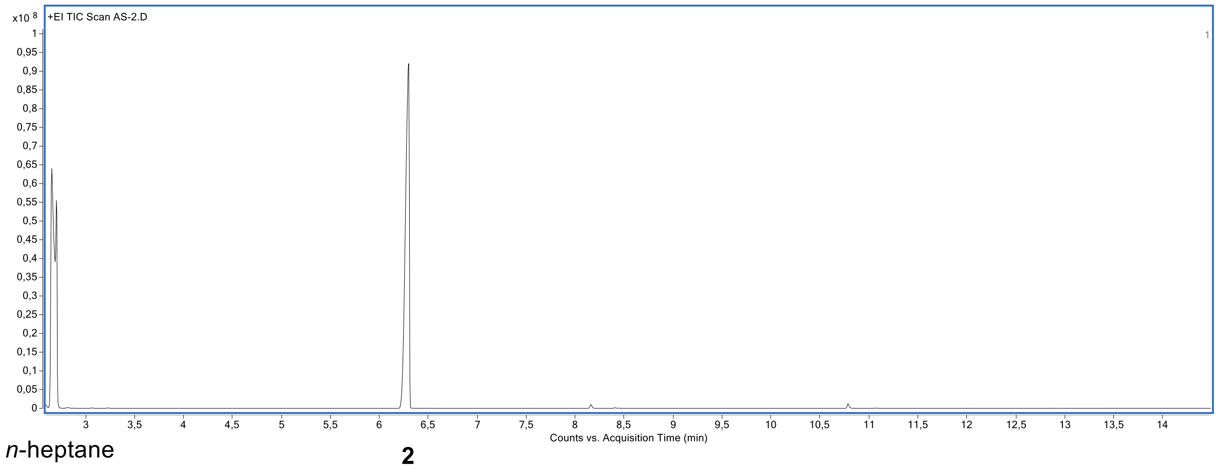

Figure S4: GC/MS chromatogram after attempted re-lithiation of **2** *via* microwave radiation.

Variant B:

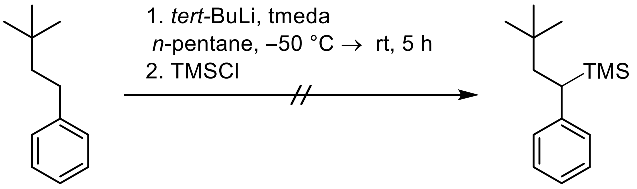

2
$C_{12}H_{18}$
162.14 g/mol

11
$C_{15}H_{26}Si$
234.18 g/mol

Dry *n*-pentane (1.0 mL) was added to an evacuated Schlenk flask. **2** (162 mg, 1.0 mmol, 1.0 eq.) and TMEDA (116 mg, 1.0 eq., 1.0 mmol) were added. The reaction solution was cooled to –50 °C and *tert*-butyllithium (0.52 mL, 1.9 M in pentane, 1.0 mmol, 1.0 eq.) was added. The cooling bath was removed and the reaction solution was stirred for five hours. No coloration of the reaction solution to red was observed. TMSCl (108 mg, 1.0 mmol, 1.0 eq.) was added and a GC/MS sample was taken. No trapping product **11** was found.

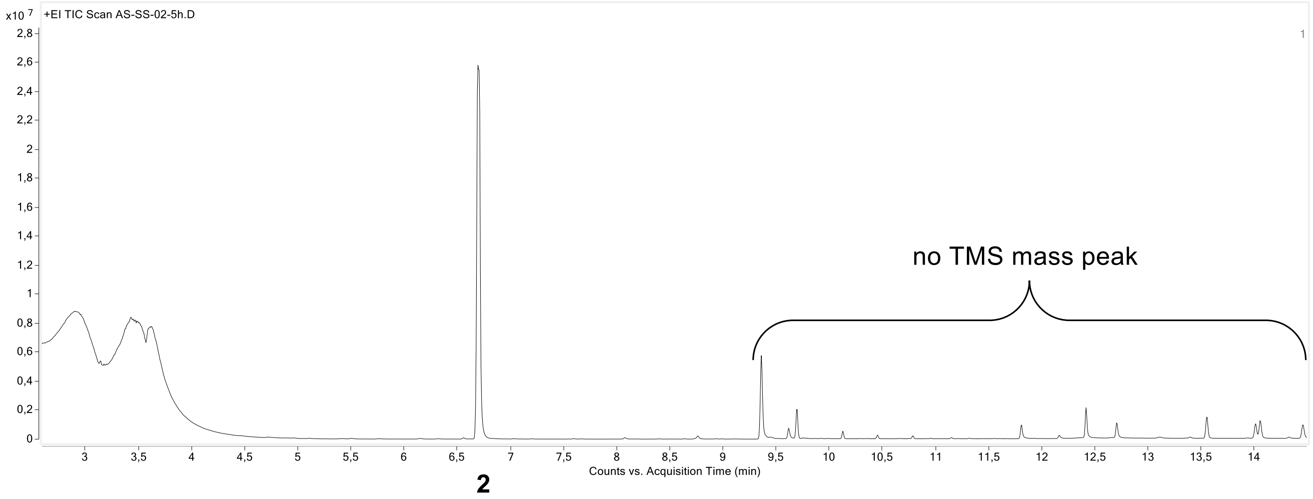

Figure S5: GC/MS chromatogram after attempted re-lithiation of **2** *via tert*-BuLi and tmeda.

Variant C:

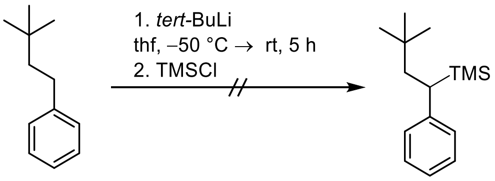

2
$C_{12}H_{18}$
162.14 g/mol

11
$C_{15}H_{26}Si$
234.18 g/mol

Dry thf (1.0 mL) was added to an evacuated Schlenk flask. **2** (162 mg, 1.0 mmol, 1.0 eq.) was added. The reaction solution was cooled to –50 °C and *tert*-butyllithium (0.52 mL, 1.9 M in pentane, 1.0 mmol, 1.0 eq.) was added. The cooling bath was removed and the reaction solution was stirred for five hours. No coloration of the reaction solution to red was observed. TMSCl (108 mg, 1.0 mmol, 1.0 eq.) was added and a GC/MS sample was taken. No trapping product **11** was found.

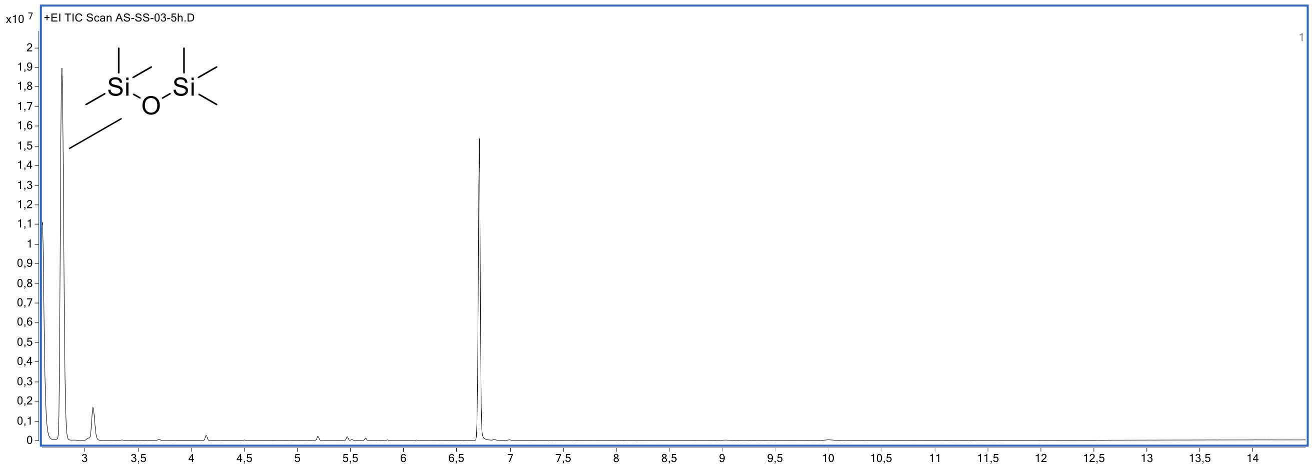

Figure S6: GC/MS chromatogram after attempted re-lithiation of **2** *via tert*-BuLi in thf.

Synthesis of addition product of *tert*-butyl lithium and styrene with diethyl ether 3

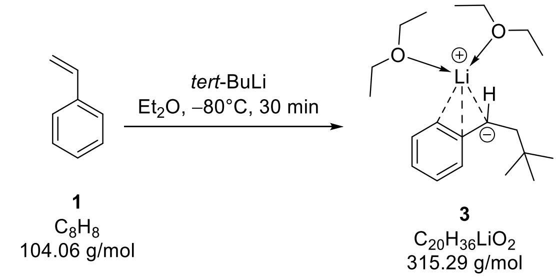

Diethyl ether (0.3 mL) was added to an evaporated Schlenk flask. Styrene (104 mg, 1.0 mmol, 1.0 eq.) was added and the reaction solution was cooled to –80 °C. *tert*-Butyllithium (0.52 mL, 1.9 M in pentane, 1.0 mmol, 1.0 eq.) was added and the reaction solution turned red. The reaction solution was stirred for 30 min at –80 °C and overlayered with dry *n*-pentane. After storage at –80 °C solid crystals could be observed. Further analytics can be obtained in Results and Discussion.

Unsuccessful synthesis of 4 from direct carbolithiation

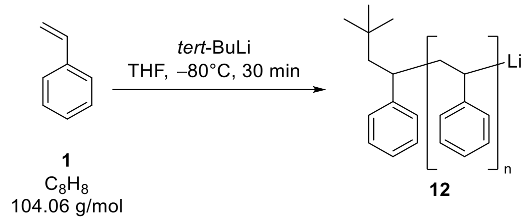

Tetrahydrofuran (1.0 mL) was added to an evaporated Schlenk flask. Styrene (104 mg, 1.0 mmol, 1.0 eq.) was added and the reaction solution was cooled to –80 °C. *tert*-Butyllithium (0.52 mL, 1.9 M in pentane, 1.0 mmol, 1.0 eq.) was added and the reaction solution turned red. The reaction solution was stirred for 30 min at –80 °C. From a GC/MS sample no monoaddition product could be observed. The reaction solution was quenched with distilled water. The aqueous phase was extracted with 3 x diethyl ether and the combined organic phases were dried with $MgSO_4$ and the solvent was evaporated in vacuo. The NMR sample reveals unselective polymer formation as products.

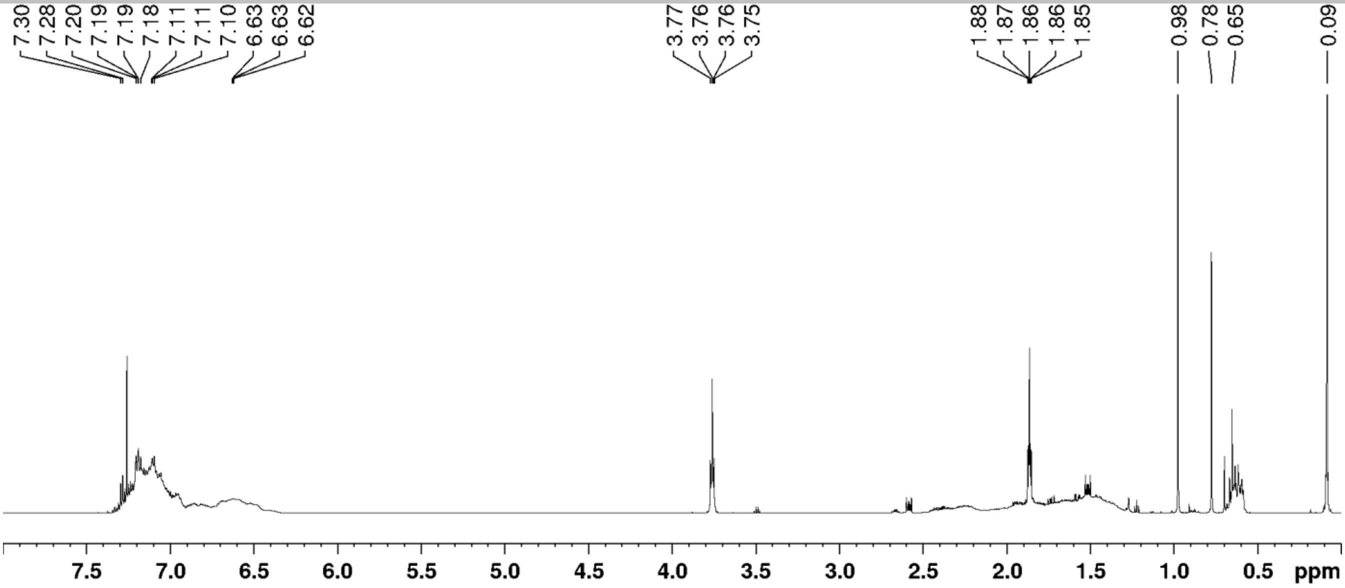

Figure S7: ^{1}H-NMR spectrum of unsuccessful synthesis of **4** from direct carbolithiation.

Synthesis of addition product of *tert*-butyl lithium and styrene with thf (4)

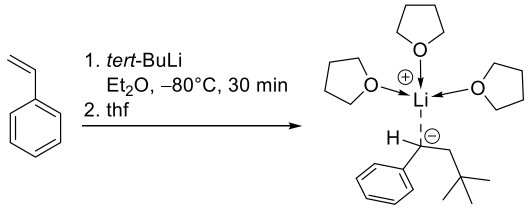

1
C_8H_8
104.06 g/mol

4
$C_{24}H_{41}LiO_3$
384.32 g/mol

Diethyl ether (0.5 mL) was added to an evaporated Schlenk flask. Styrene (104 mg, 1.0 mmol, 1.0 eq.) was added and the reaction solution was cooled to –80 °C. *tert*-Butyllithium (0.52 mL, 1.9 M in pentane, 1.0 mmol, 1.0 eq.) was added and the reaction solution turned red. The reaction solution was stirred for 30 min at –80 °C, tetrahydrofuran (0.5 mL) was added and the reaction solution was overlayered with dry *n*-pentane. After storage at –80 °C solid crystals could be observed. Further analytics can be obtained in Results and Discussion.

***In situ* FTIR studies**

a) 1,1-diphenylethylene, thf

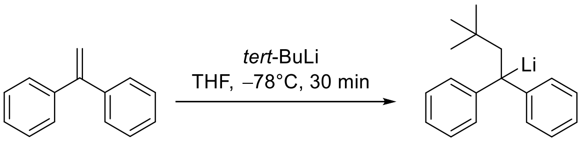

13
$C_{14}H_{12}$
180.09 g/mol

14
$C_{18}H_{21}Li$
244.18 g/mol

Two evaporated Schlenk flasks were prepared. Dry thf (2.0 mL) and 1,1-diphenylethylene (180 mg, 1.0 mmol, 1.0 eq.) were added to flask B, which was subsequently cooled at –78 °C in a dry ice bath. An *in situ* FTIR probe was added to identify the IR bands of 1,1-diphenylethylene at the given conditions. The probe was removed and cleaned. Dry thf (3.0 mL) was added to flask A and was cooled at –78 °C in a dry ice bath. An *in situ* FTIR probe was inserted under argon atmosphere and the temperature was tracked until constancy was achieved. Subsequently, *tert*-butyllithium was added (0.52 mL, 1.9 M in pentane, 1.0 mmol, 1.0 eq.) and after constant intensity of the newly arose frequency bands, the solution from flask B was added. The reaction solution was stirred until completeness was reached.

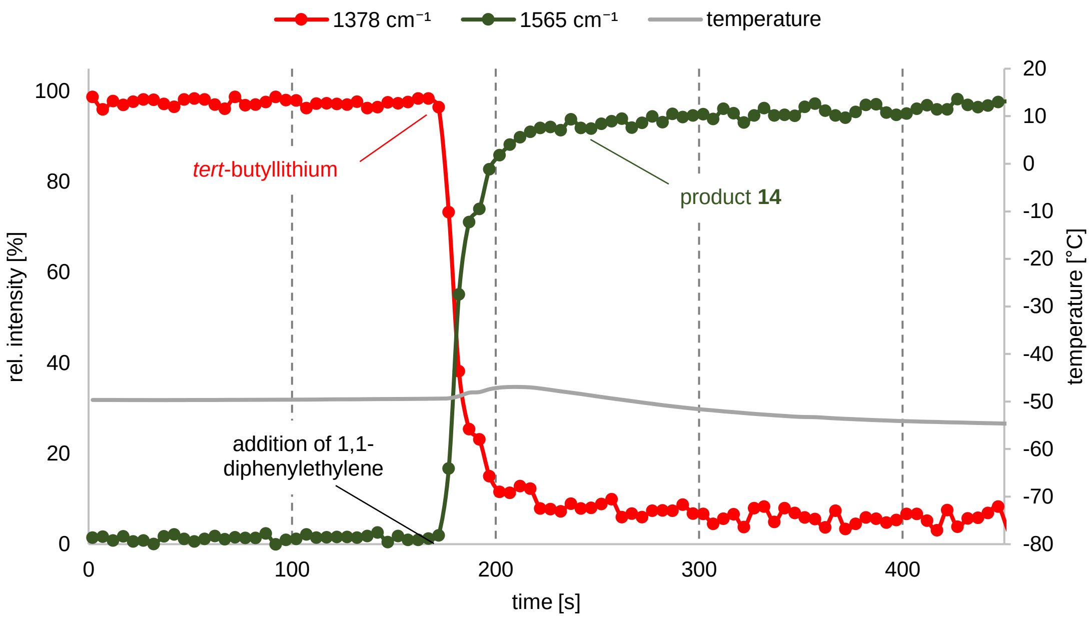

Figure S8: Plot of the normalized intensities of IR bands 1378 cm^{-1} and 1565 cm^{-1} against time for *in situ* FTIR experiment of addition of *tert*-butyllithium to 1,1-diphenylethylene in thf at –78 °C.

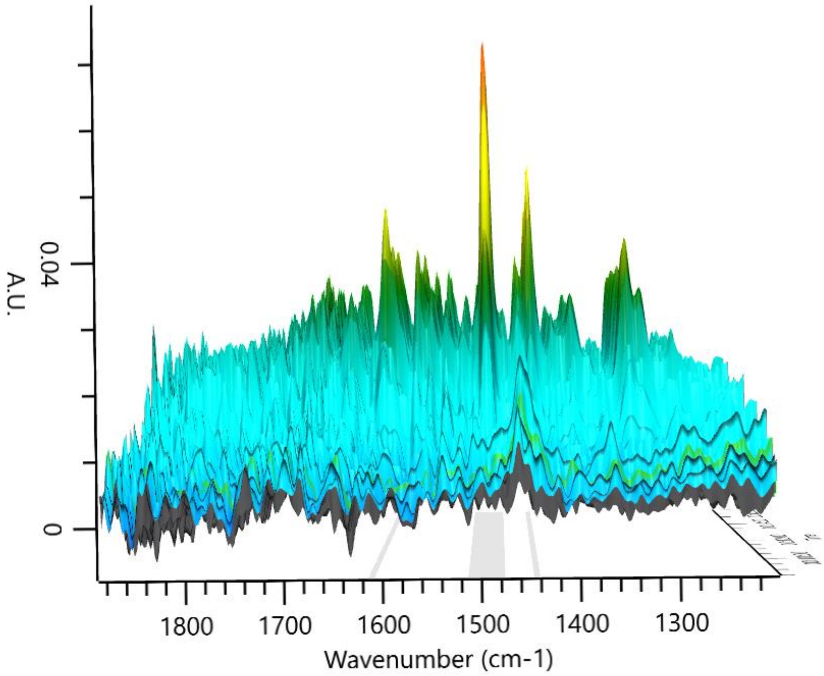

Figure S9: Excerpt from 3D surface of *in situ* FTIR experiment of 1,1-dipehnylethylene in thf at –78 °C.

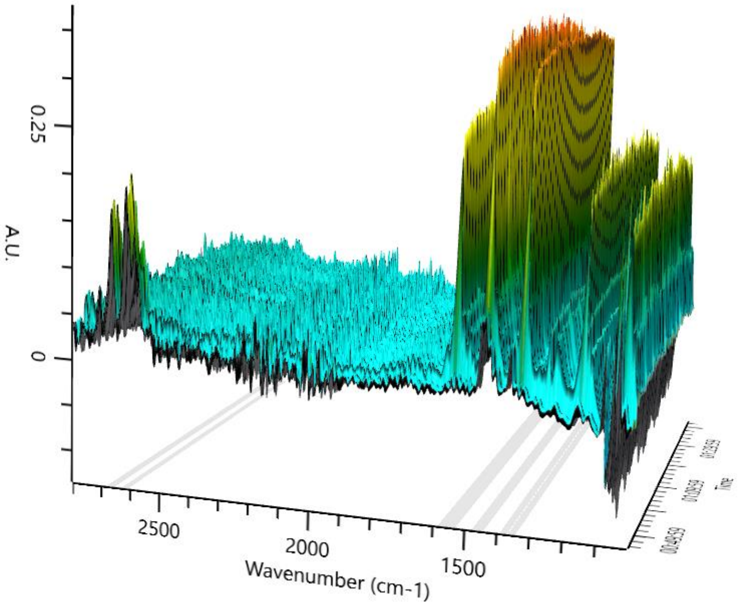

Figure S10: Excerpt from 3D surface of *in situ* FTIR experiment of addition of *tert*-butyllithium to 1,1-diphenylethylene in thf at –78 °C.

b) 1,1-diphenylethylene, diethyl ether

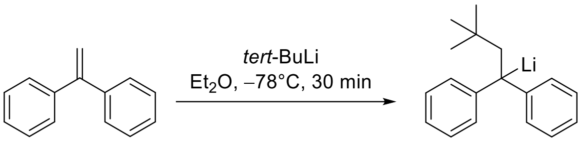

13
$C_{14}H_{12}$
180.09 g/mol

14
$C_{18}H_{21}Li$
244.18 g/mol

Two evaporated Schlenk flasks were prepared. Dry diethyl ether (2.0 mL) and 1,1-diphenylethylene (180 mg, 1.0 mmol, 1.0 eq.) were added to flask B, which was subsequently cooled at –78 °C in a dry ice bath. Dry diethyl ether (3.0 mL) was added to flask A and was cooled at –78 °C in a dry ice bath. An *in situ* FTIR probe was inserted under argon atmosphere and the temperature was tracked until constancy was achieved. Subsequently, *tert*-butyllithium was added (0.52 mL, 1.9 M in pentane, 1.0 mmol, 1.0 eq.) and after constant intensity of the newly arose frequency bands, the solution from flask B was added. The reaction solution was stirred until completeness was reached.

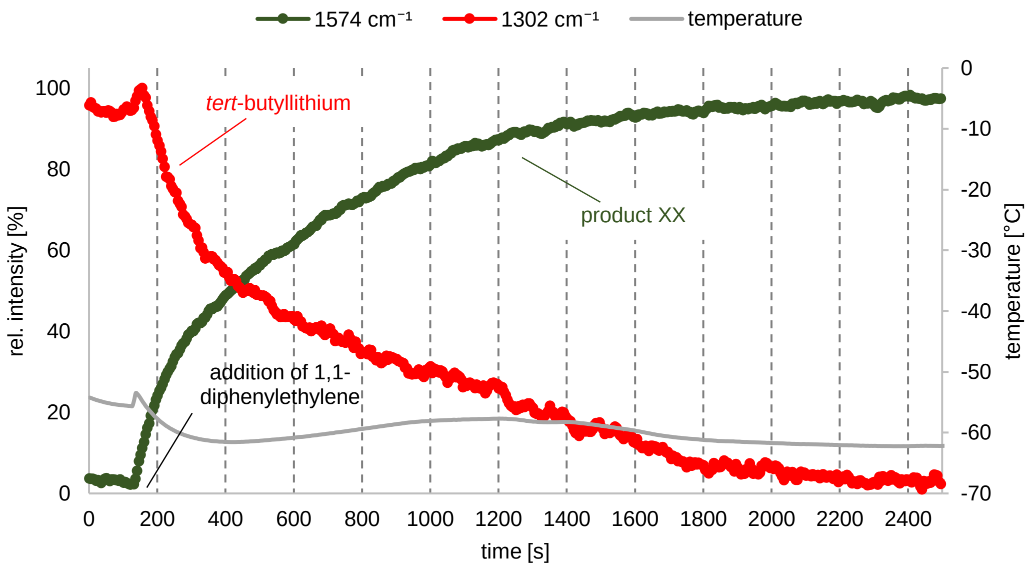

Figure S11: Plot of the normalized intensities of IR bands 1302 cm^{-1} and 1574 cm^{-1} against time for *in situ* FTIR experiment of addition of *tert*-butyllithium to 1,1-diphenylethylene in diethyl ether at –78 °C.

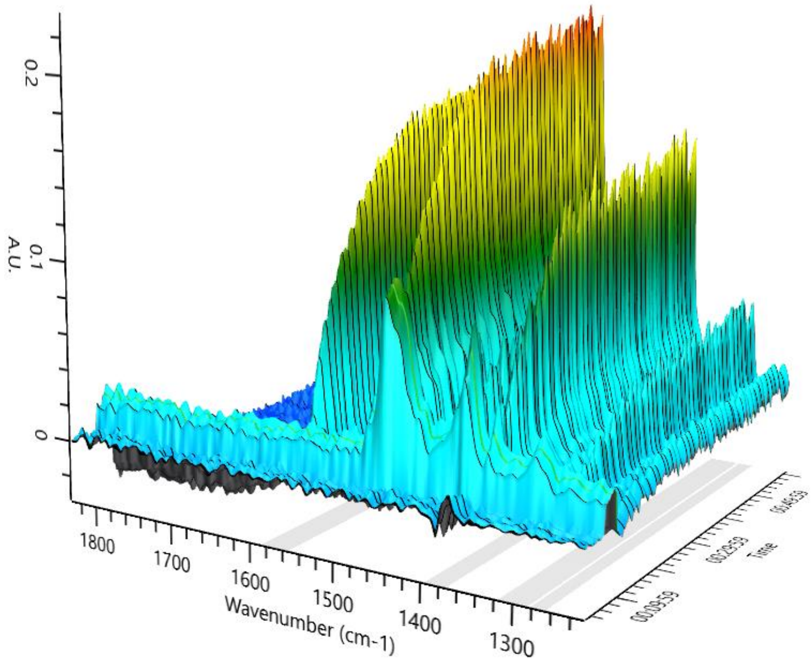

Figure S12: Excerpt from 3D surface of *in situ* FTIR experiment of addition of *tert*-butyllithium to 1,1-diphenylethylene in diethyl ether at –78 °C.

c) styrene, diethyl ether

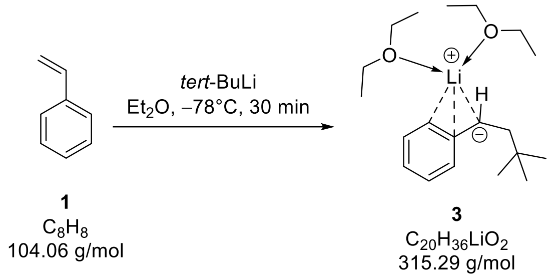

Two evaporated Schlenk flasks were prepared. Dry diethyl ether (2.0 mL) and styrene (104 mg, 1.0 mmol, 1.0 eq.) were added to flask B, which was subsequently cooled at –78 °C in a dry ice bath. Dry diethyl ether (3.0 mL) was added to flask A and was cooled at –78 °C in a dry ice bath. An *in situ* FTIR probe was inserted under argon atmosphere and the temperature was tracked until constancy was achieved. Subsequently, *tert*-butyllithium was added (0.52 mL, 1.9 M in pentane, 1.0 mmol, 1.0 eq.) and after constant intensity of the newly arose frequency bands, the solution from flask B was added. The reaction solution was stirred until completeness was reached. The plot of the normalized intensity of the relevant IR bands against the time can be seen in the manuscript.

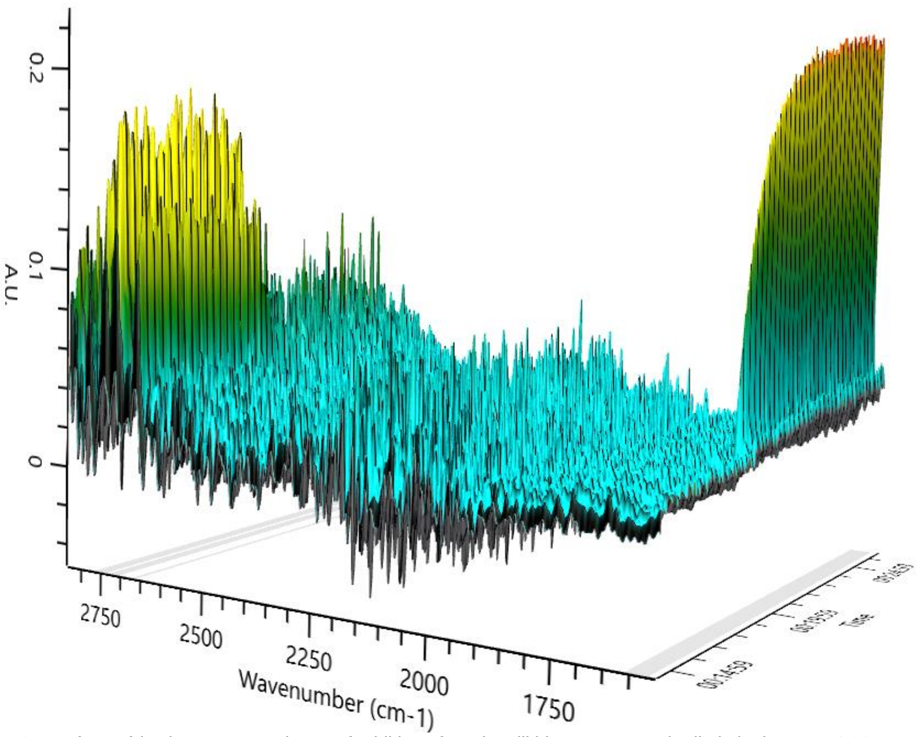

Figure S13: Excerpt from 3D surface of *in situ* FTIR experiment of addition of *tert*-butyllithium to styrene in diethyl ether at –78 °C.

Results and Discussion

Single crystal X-ray diffraction studies

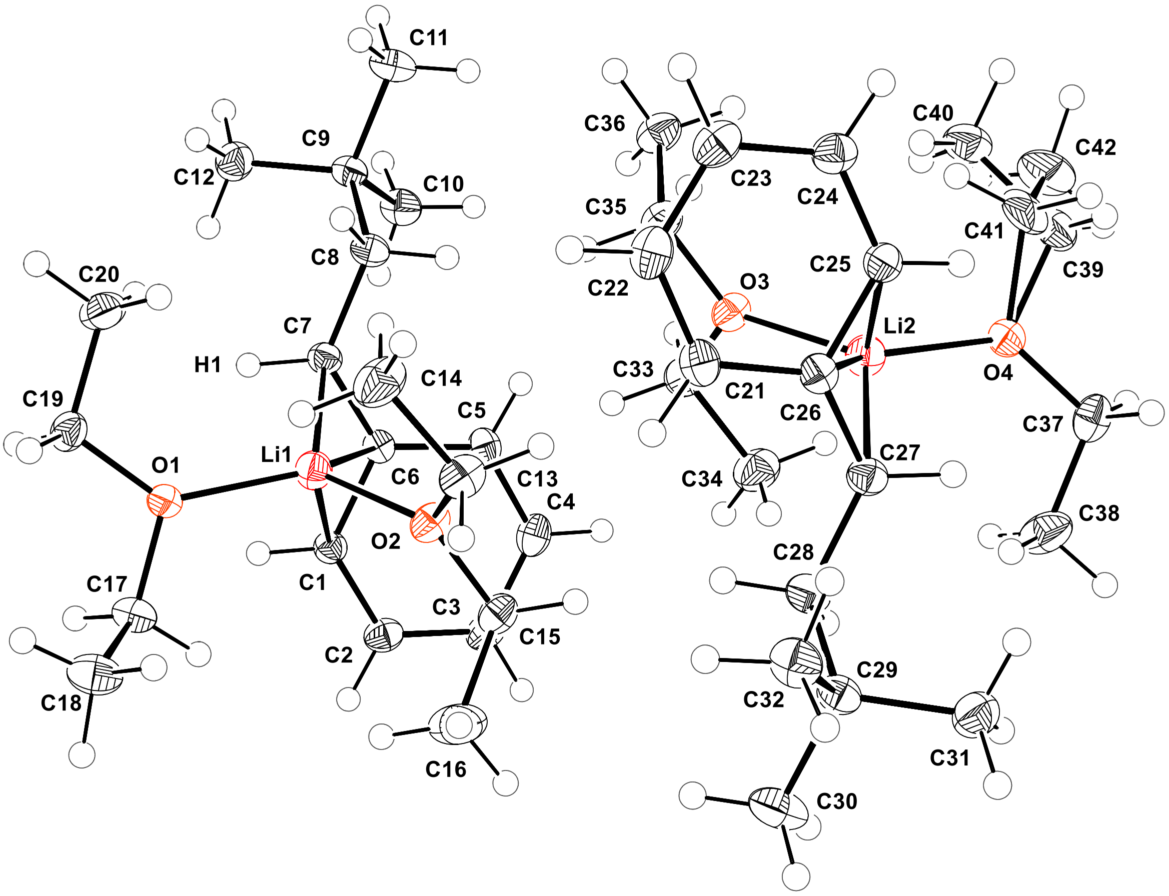

Figure S14: ORTEP plot of **3**. Selected bond lengths [Å] and angles [°]: O1–Li1 1.9431(12); O2–Li1 1.9345(12); C7–Li1 2.1927(12); C6–Li1 2.3181(12); C1–Li1 2.4215(13); C6–C7 1.4063(8); C7–C8 1.5072(8); C6–C7–C8 126.24(5); O1–Li1–O2 114.49(6); O1–Li1–C7 115.24(5); O2–Li1–C7 128.70(6). Disorder of the ethyl group C39–C40 (93%): C41–C42 (7%). CCDC deposition number: 2444479.[10]

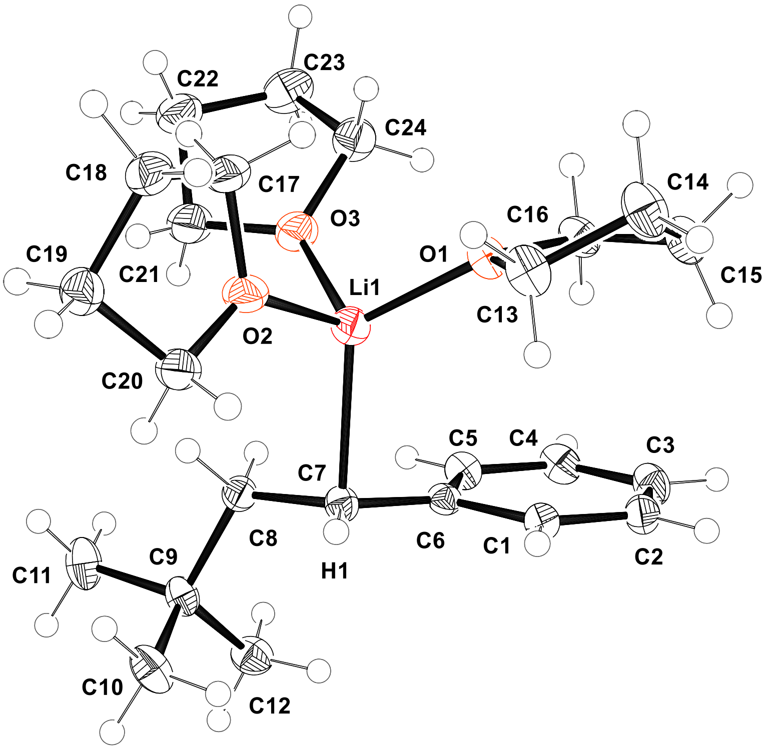

Figure S15: ORTEP plot of **4**. Selected bond lengths [Å] and angles [°]: O1–Li1 1.9493(17); O2–Li1 1.9581(18); O3–Li1 1.9754(18); C7–Li1 2.2363(18); C6–C7 1.4116(11); C7–C8 1.5094(12); C6–C7–C8 125.20(7); O1–Li1–O2 101.23(8); O1-Li1-O3 106.12(8); O2–Li1–O3 101.53(8); O1–Li1–C7 121.02(8); O2–Li1–C7 106.76(8); O3–Li1–C7 117.21(8). CCDC deposition number: 2444480.[10]

Table S1: Crystallographic data and structure refinement of the compounds **3** and **4**.

Identification code	3	4
Empirical formula	$C_{20}H_{37}LiO_2$	$C_{24}H_{41}LiO_3$
Formula weight	316.43	384.51
Temperature/K	100.00	100.00
Crystal system	monoclinic	monoclinic
Space group	$P2_1/c$	$P2_1/n$
a/Å	18.331(2)	8.797(3)
b/Å	9.9214(11)	11.526(4)
c/Å	24.160(3)	24.013(8)
α/°	90	90
β/°	109.979(4)	94.895(4)
γ/°	90	90
Volume/Å^3	4129.4(8)	2426.1(14)
Z	8	4
ρ_{calc}g/cm^3	1.018	1.053
μ/mm^{-1}	0.062	0.066
F(000)	1408.0	848.0
Crystal size/mm^3	0.704 × 0.57 × 0.392	0.999 × 0.608 × 0.27
Radiation	MoKα (λ = 0.71073)	MoKα (λ = 0.71073)
2Θ range for data collection/°	3.558 to 72.802	3.922 to 67.52
Index ranges	$-30 \le h \le 30$, $-16 \le k \le 16$, $-40 \le l \le 40$	$-11 \le h \le 13$, $-16 \le k \le 17$, $-37 \le l \le 37$
Reflections collected	574035	102984
Independent reflections	20085 [R_{int} = 0.0458, R_{sigma} = 0.0131]	9682 [R_{int} = 0.0682, R_{sigma} = 0.0289]
Data/restraints/parameters	20085/0/457	9682/0/260
Goodness-of-fit on F^2	1.047	1.037
Final R indexes [I>=2σ (I)]	R_1 = 0.0417, wR_2 = 0.1213	R_1 = 0.0495, wR_2 = 0.1313
Final R indexes [all data]	R_1 = 0.0556, wR_2 = 0.1321	R_1 = 0.0682, wR_2 = 0.1458
Largest diff. peak/hole / e Å^{-3}	0.49/-0.25	0.55/-0.19

NMR studies

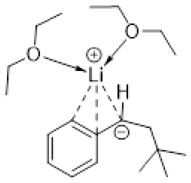

3
$C_{20}H_{38}LiO_2$
315.29 g/mol

^{1}H-NMR: (600 MHz, toluene-d_8): δ = 0.96 (t, $^3J_{HH}$ = 7.0 Hz, 12H; ether-CH_3), 1.01 (s, 9H; c(CH_3)$_3$), 1.88 (d, $^3J_{HH}$ = 6.6 Hz, 2H; CH^--CH_2-C(CH_3)$_3$), 2.76 (t, $^3J_{HH}$ = 6.6 Hz, 1H; CH^--CH_2-C(CH_3)$_3$), 3.20 (q, $^3J_{HH}$ = 7.0 Hz, 8H; ether-CH_2), 5.19 (t, $^3J_{HH}$ = 6.6 Hz, 1H; para-*H*), 5.80 (m, 2H; ortho-*H*), 6.44 (m, 2H; meta-*H*) ppm.
^{13}C{^{1}H}-NMR: (400 MHz, toluene-d_8): δ = 15.0 (s, 4C; ether-CH_3), 30.0 (s, 3C; CCH_3), 34.1 (s, 1C; CCH$_3$),) 43.2 (s, 1C; CH_2-CH$^-$-Ph), 59.5 (s, 1C; CH_2-CH^--Ph), 65.9 (s, 4C, ether-CH_2), 97.8 (s, 1C; para-C_{Ar}), 109.6 (d, 2C; ortho-C_{Ar}), 130.4 (s, 2C; meta-C_{Ar}), 149.8 (s, 1C; iso-C_{Ar}) ppm.

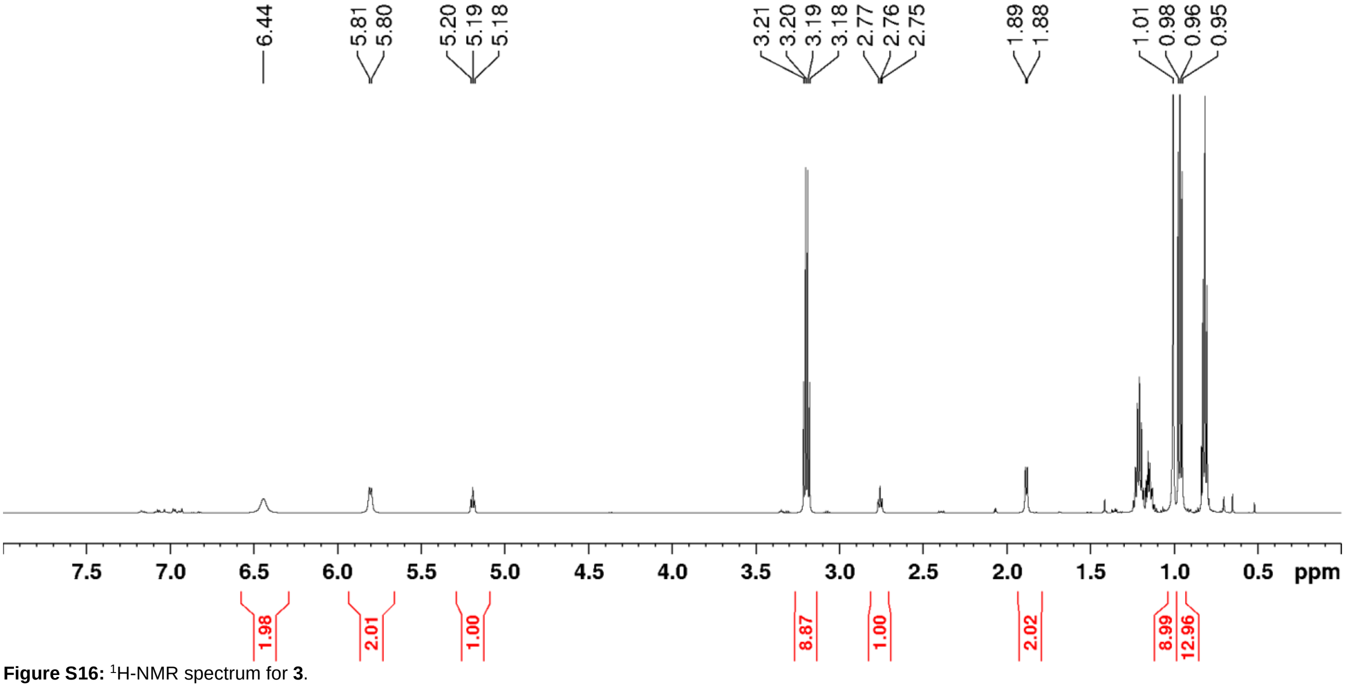

Figure S16: ^{1}H-NMR spectrum for **3**.

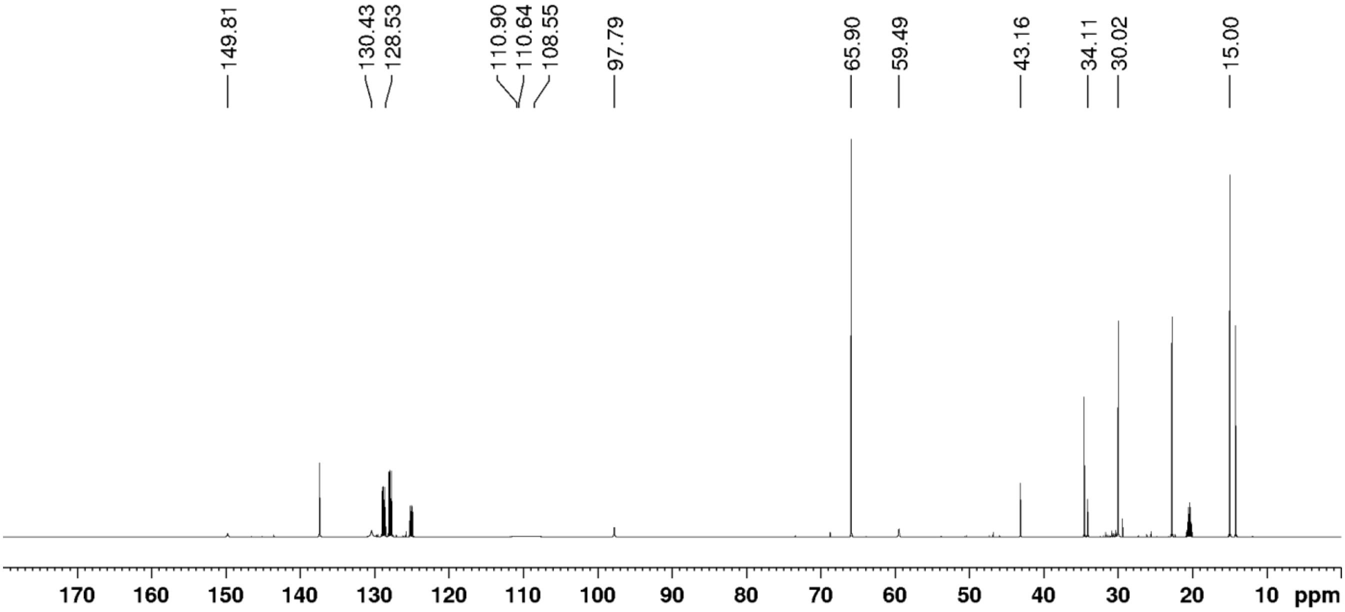

Figure S17: ^{13}C-NMR spectrum of **4**.

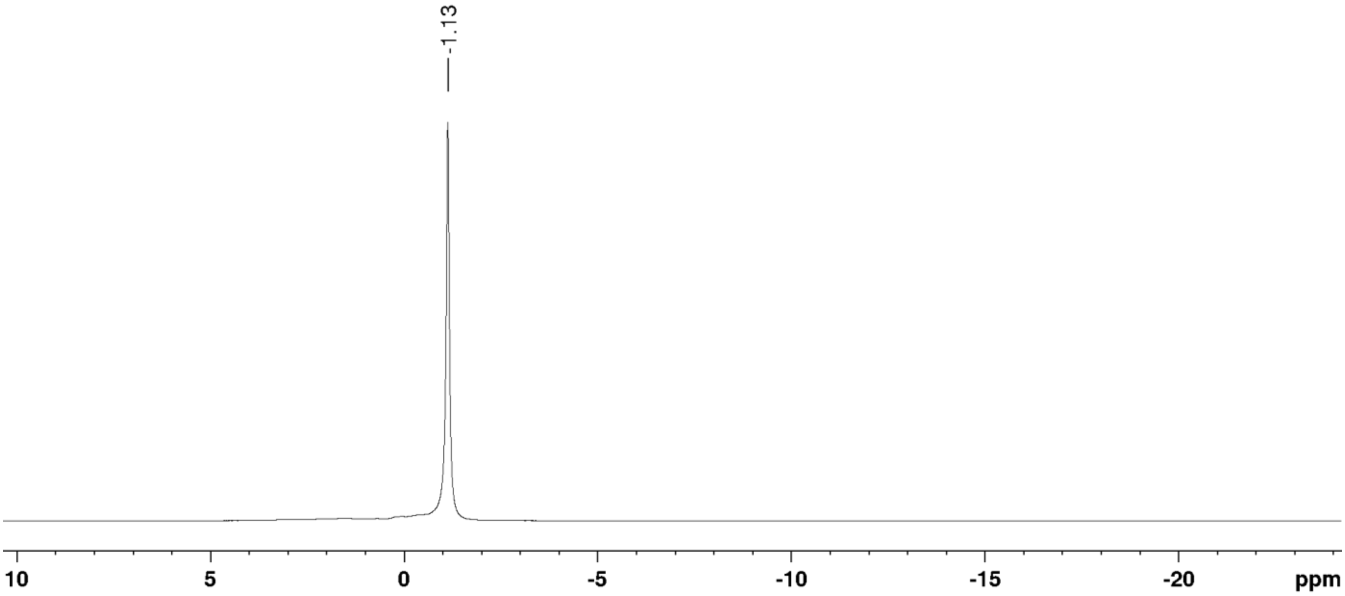

Figure S18: ^{7}Li-NMR of **3**.

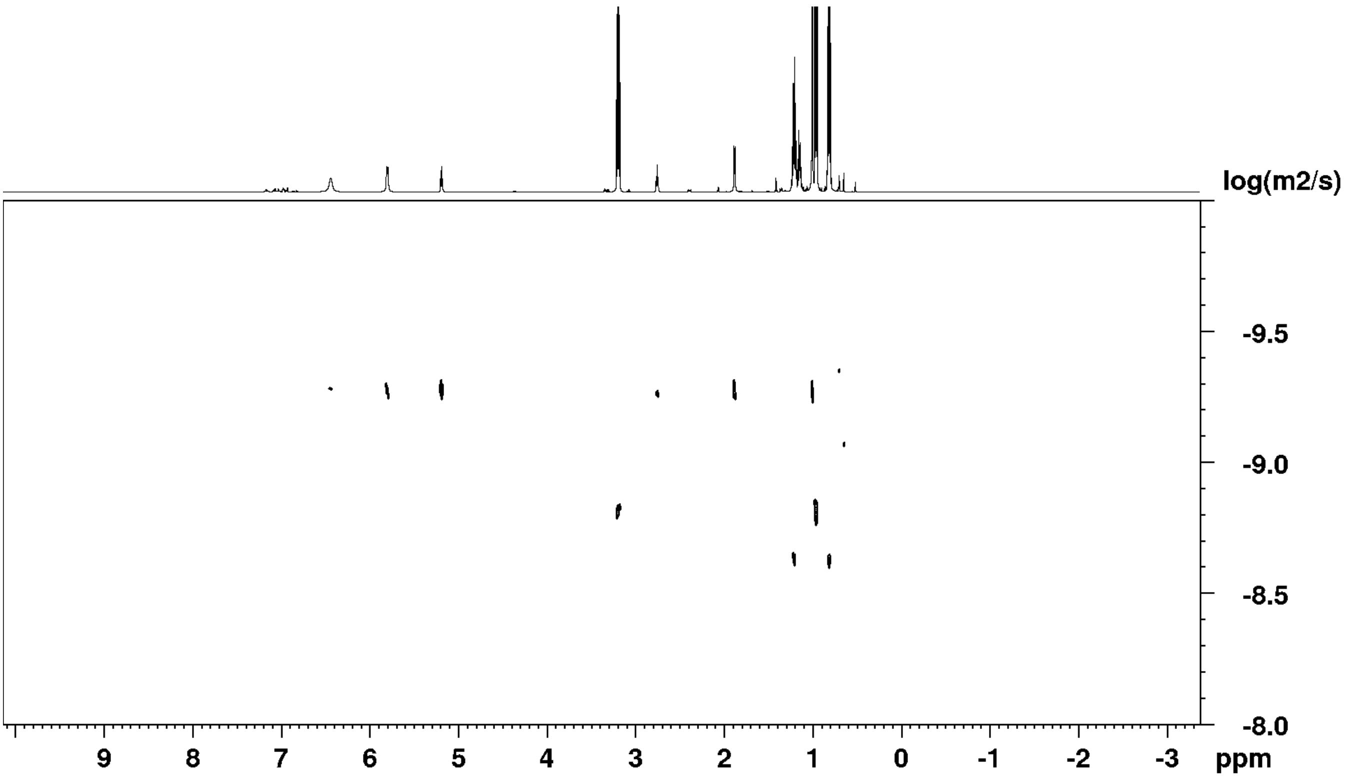

Figure S19: ^{1}H-DOSY-NMR of **3**.

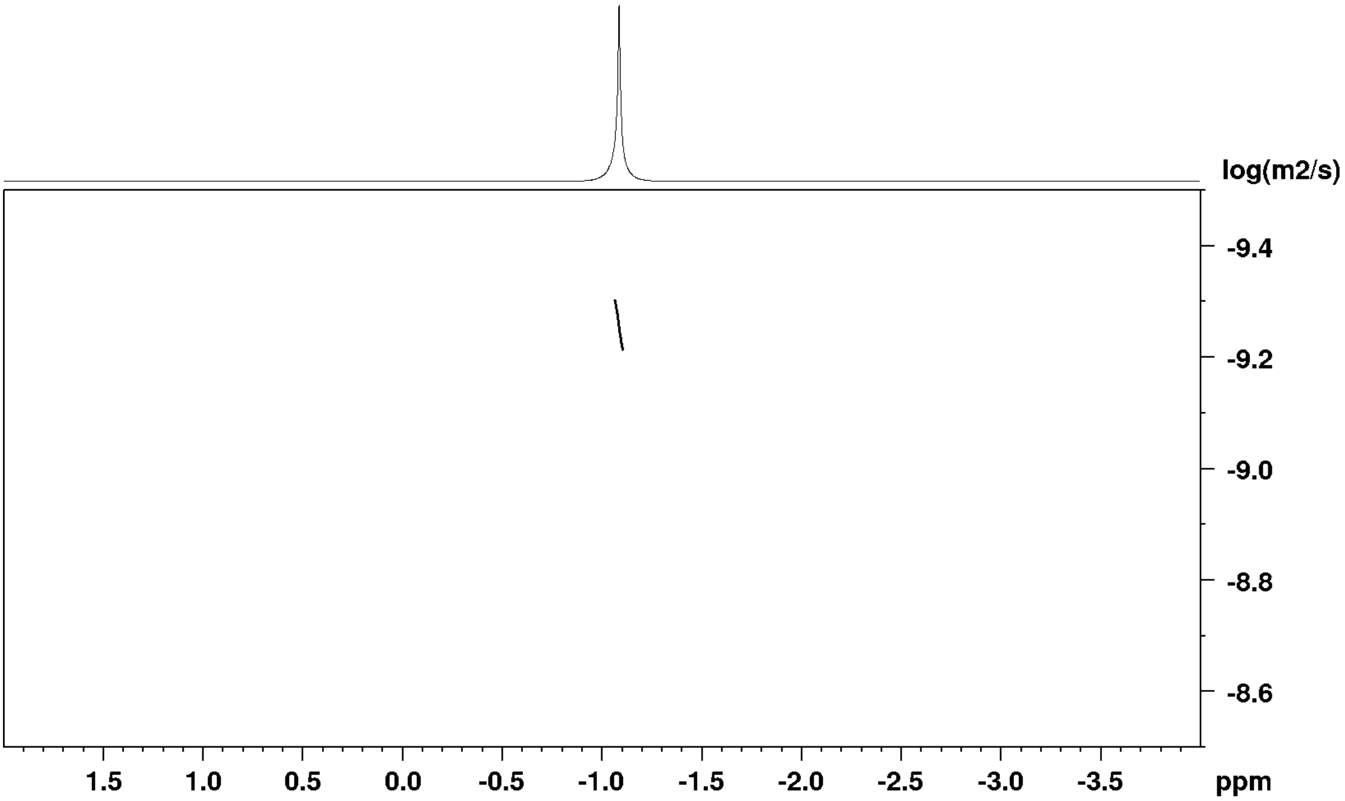

Figure S20: ^{7}Li-DOSY NMR of **3**.

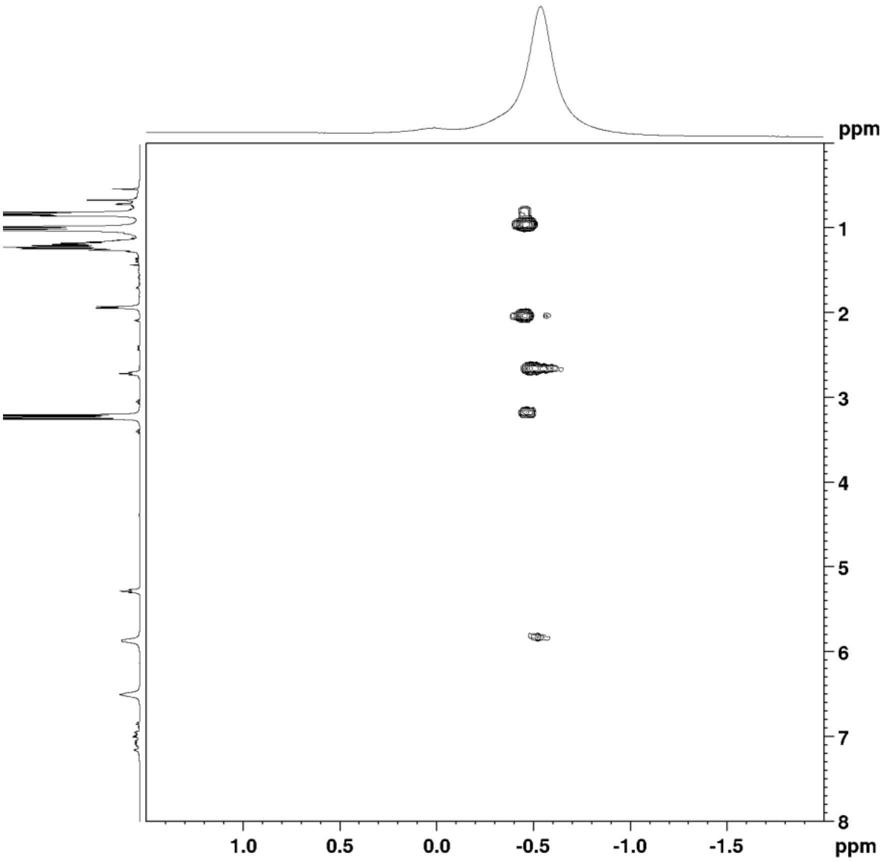

Figure S21: ^{1}H-^{7}Li-HOESY-NMR spectrum of **3**.

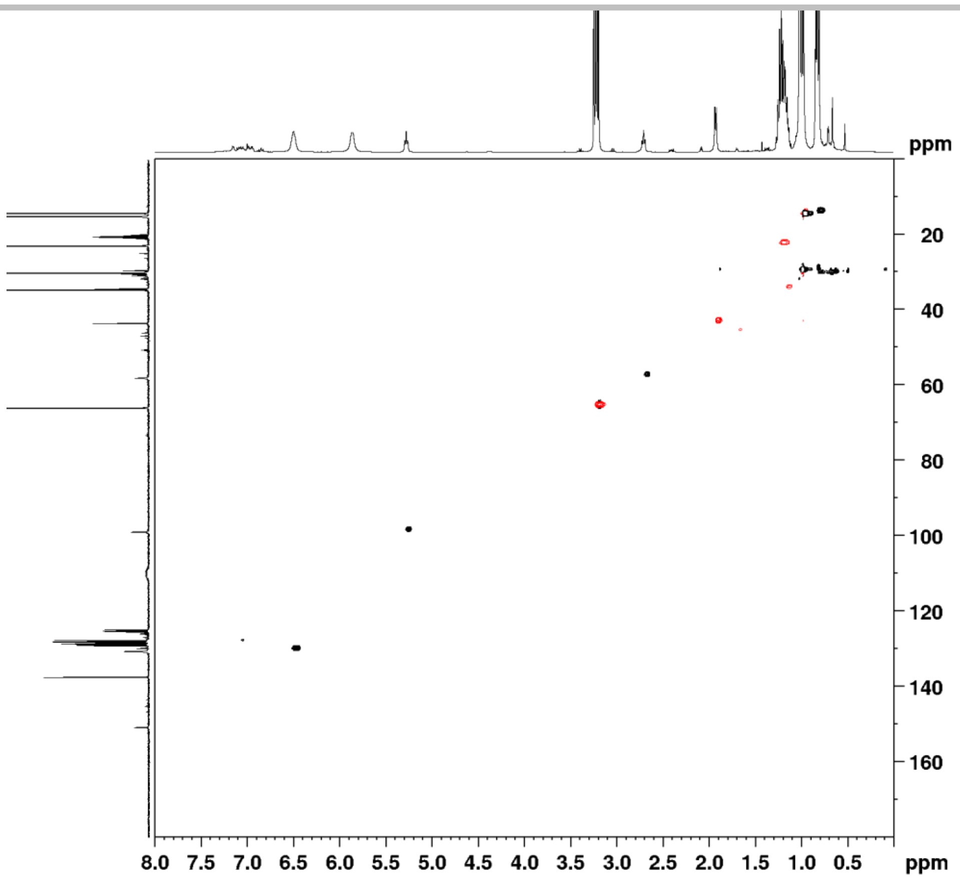

Figure S22: HSQC-NMR spectrum of **3**.

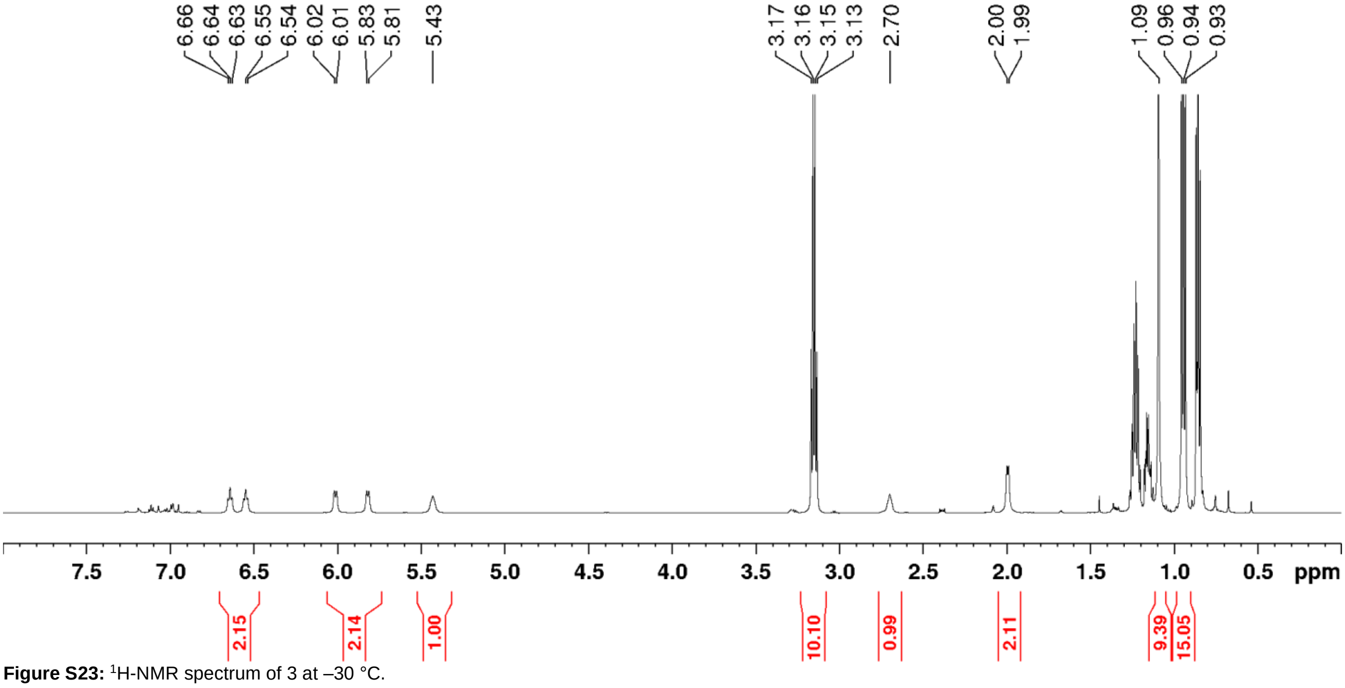

Figure S23: ^{1}H-NMR spectrum of 3 at –30 °C.

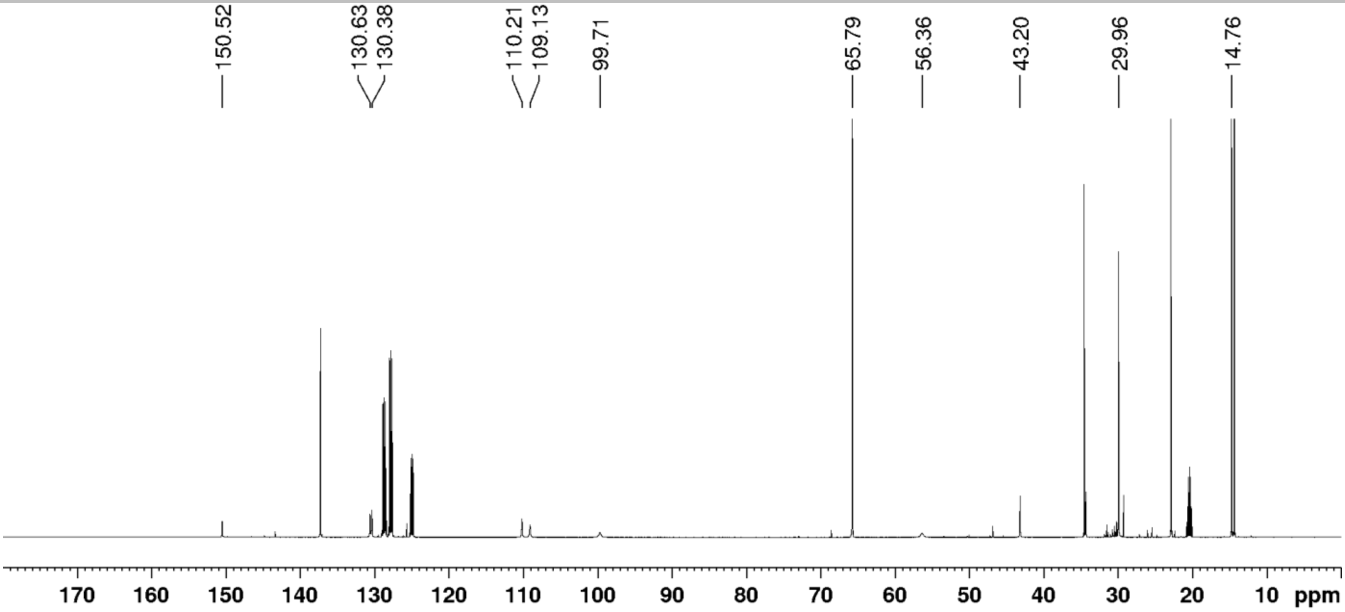

Figure S24: ^{13}C-NMR spectrum of **3** at –30 °C.

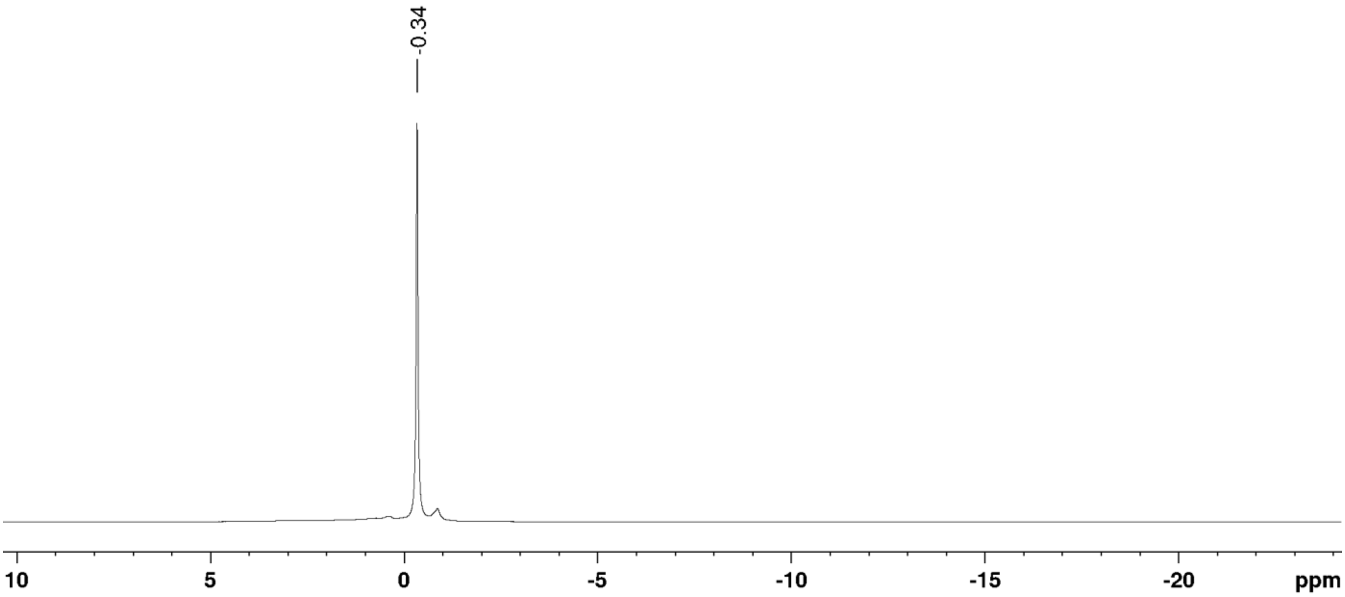

Figure S25: ^{7}Li-NMR spectrum of **3** at –30 °C.

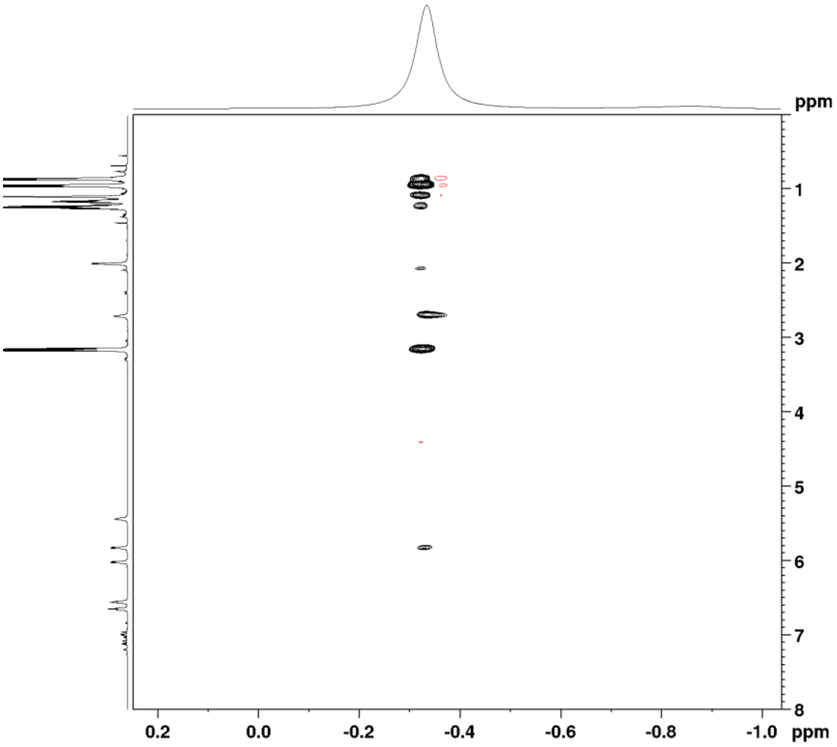

Figure S26: ^{1}H-^{7}Li-HOESY NMR spectrum of **3** at –30 °C.

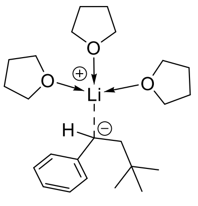

4
$C_{24}H_{41}LiO_3$
384.32 g/mol

^{1}H-NMR: (600 MHz, toluene-*d*$_8$): δ = 0.99 (s, 9H; c(C*H*$_3$)$_3$), 1.52 (m, 12H; thf-OCH$_2$-C*H*$_2$), 1.94 (d, $^{3}J_{HH}$ = 6.7 Hz, 2H; CH$^-$-C*H*$_2$-C(CH$_3$)$_3$), 2.51 (t, $^{3}J_{HH}$ = 6.7 Hz, 1H; C*H*$^-$-CH$_2$-C(CH$_3$)$_3$), 3.46 (m, 12H; thf-OC*H*$_2$-CH$_2$), 5.32 (t, $^{3}J_{HH}$ = 7.0 Hz, 1H; para-*H*), 5.88 (s, 1H; ortho-*H*), 5.93 (s, 1H; ortho-*H*), 6.43 (s, 1H; meta-*H*), 6.54 (s, 1H; meta-*H*) ppm.
^{13}C{^{1}H}-NMR: (400 MHz, toluene-*d*$_8$): δ = 25.8 (s, 6C; thf-OCH$_2$-*C*H$_2$), 30.1 (s, 3C; C*C*H$_3$), 34.6 (s, 1C; *C*CH$_3$),) 44.1 (s, 1C; *C*H$_2$-CH$^-$-Ph), 53.9 (s, 1C; CH$_2$-*C*H$^-$-Ph), 68.3 (s, 6C, thf-O*C*H$_2$-CH$_2$), 100.3 (s, 1C; para-*C*$_{Ar}$), 109.4 (s, 1C; ortho-*C*$_{Ar}$), 112.7 (s, 1C; ortho-*C*$_{Ar}$), 129.5 (s, 1C; meta-*C*$_{Ar}$), 130.0 (s, 1C; meta-*C*$_{Ar}$), 152.9 (s, 1C; iso-*C*$_{Ar}$) ppm.

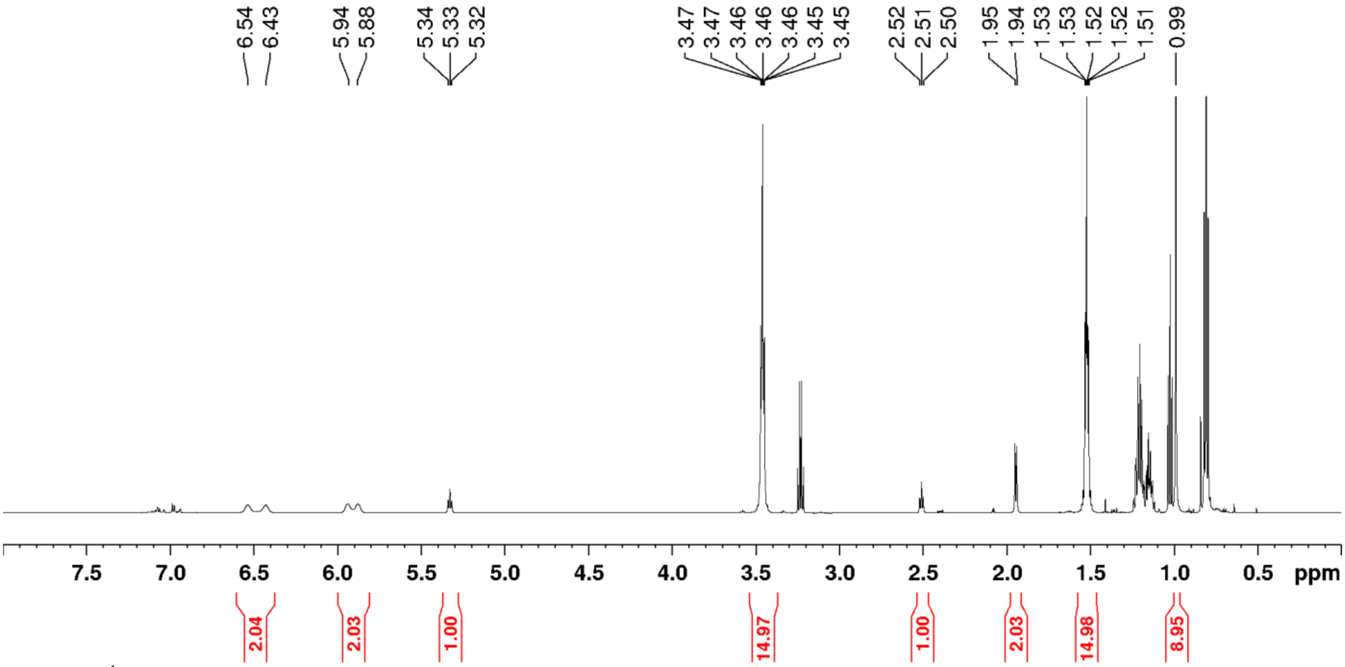

Figure S27: ^{1}H-NMR spectrum of **4**.

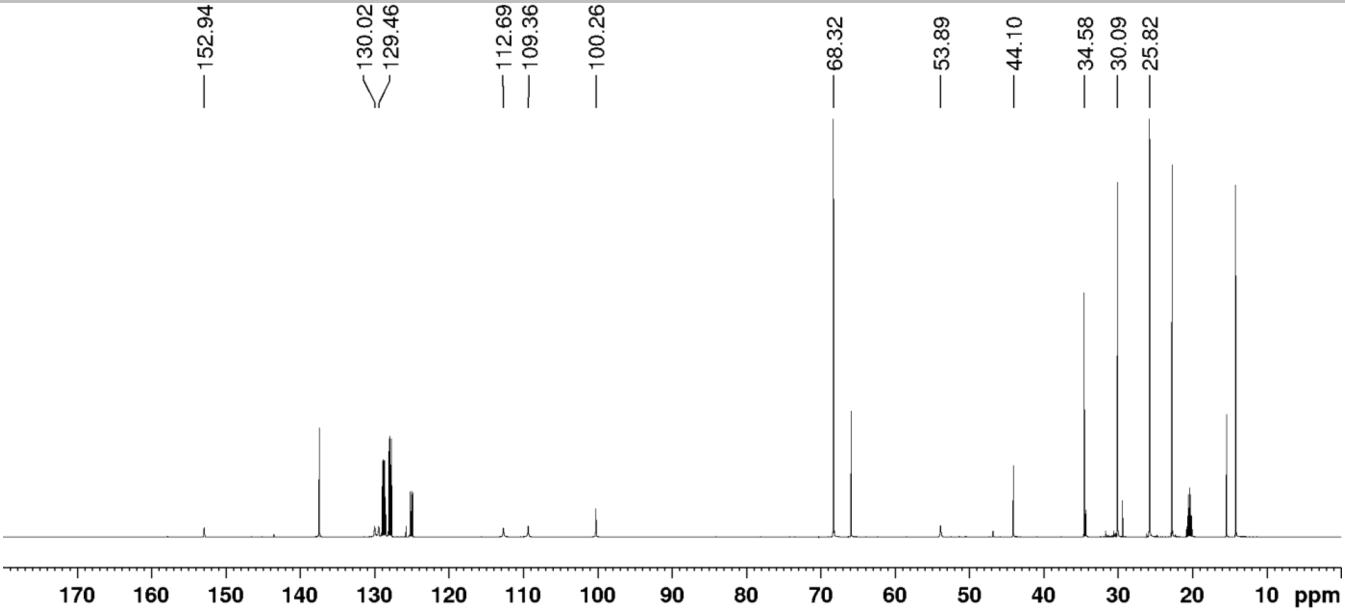

Figure S28: ^{13}C-NMR spectrum of **4**.

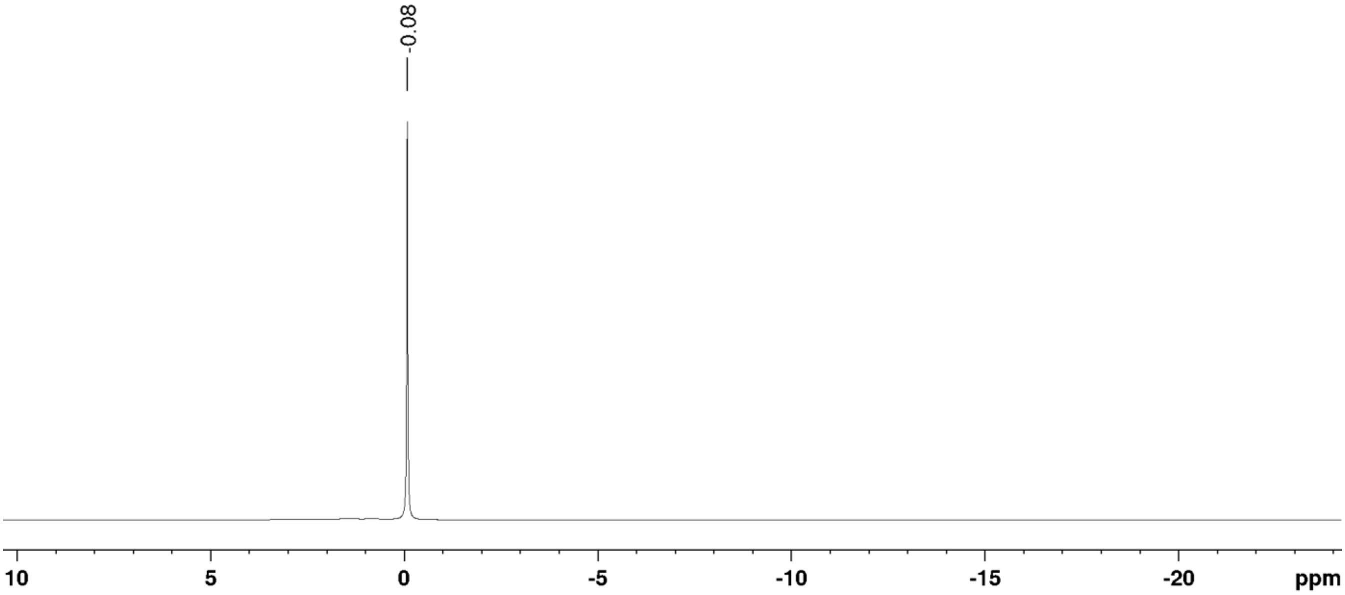

Figure S29: ^{7}Li-NMR spectrum of **4**.

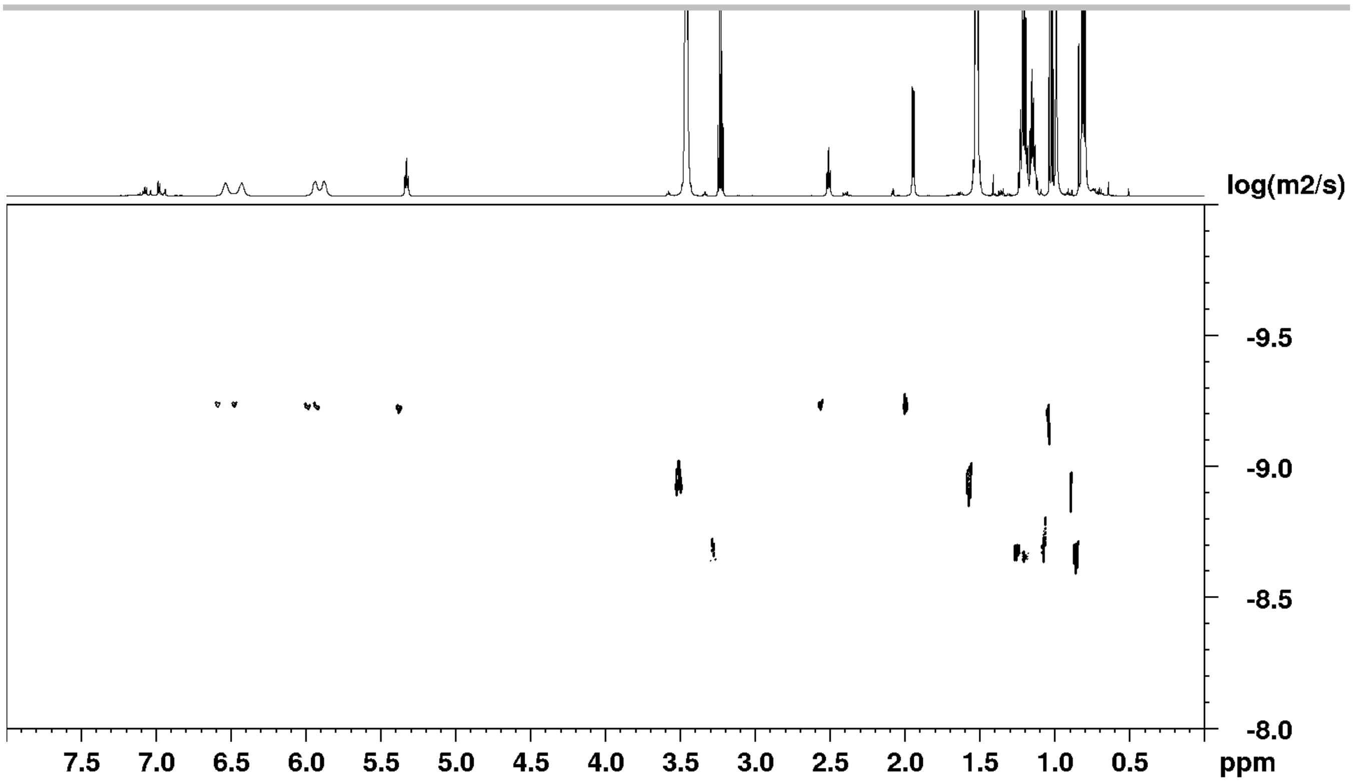

Figure S30: ^{1}H-DOSY-NMR spectrum of **4**.

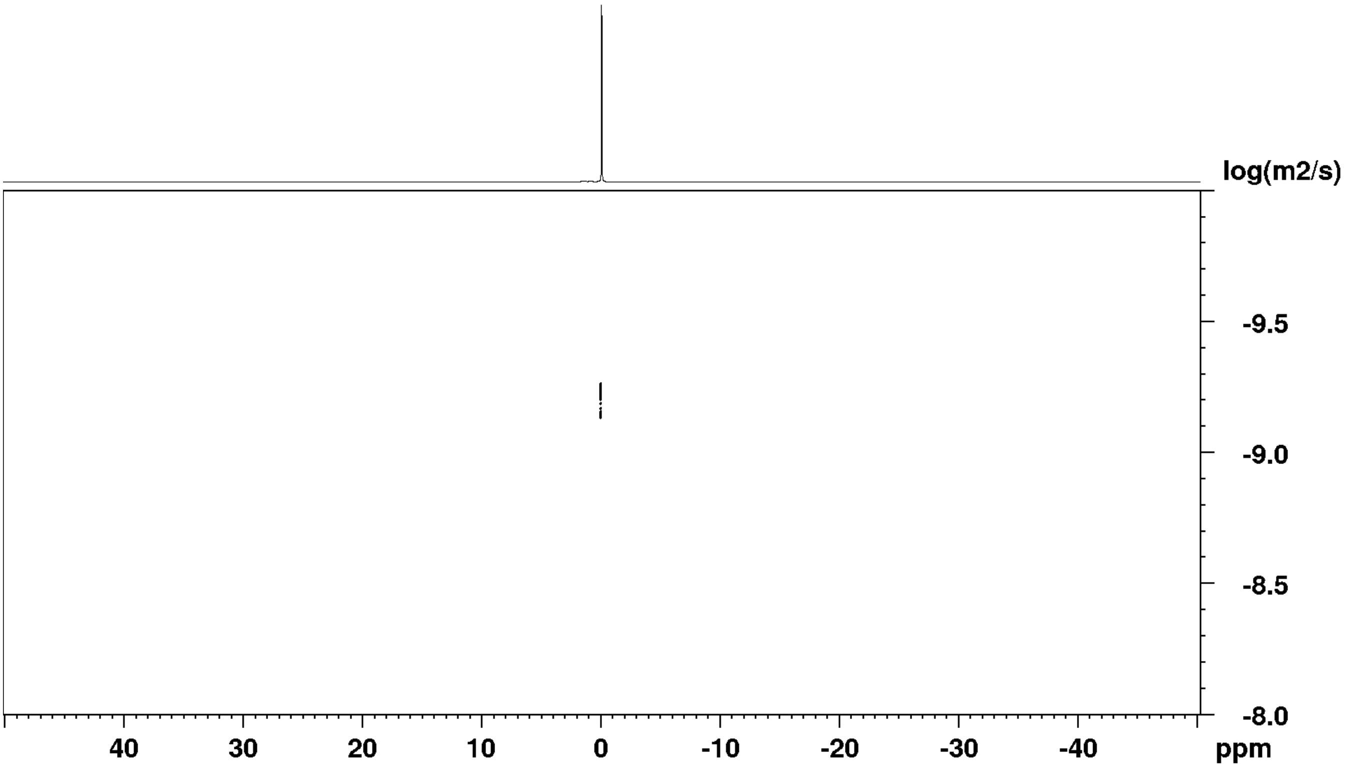

Figure S31: ^{7}Li-DOSY NMR spectrum of **4**.

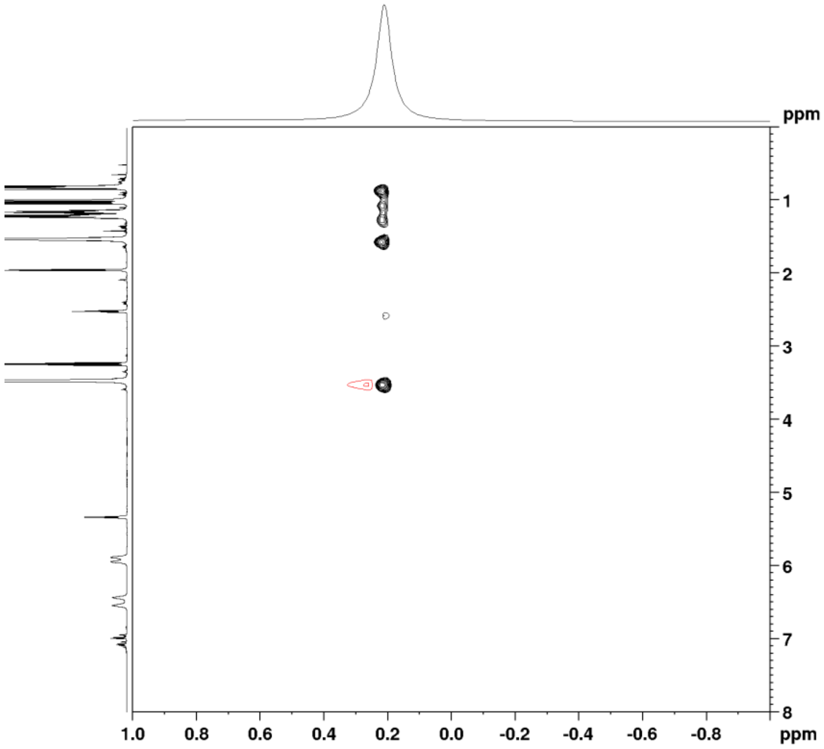

Figure S32: ^{1}H-^{7}Li-HOESY NMR spectrum of **4**.

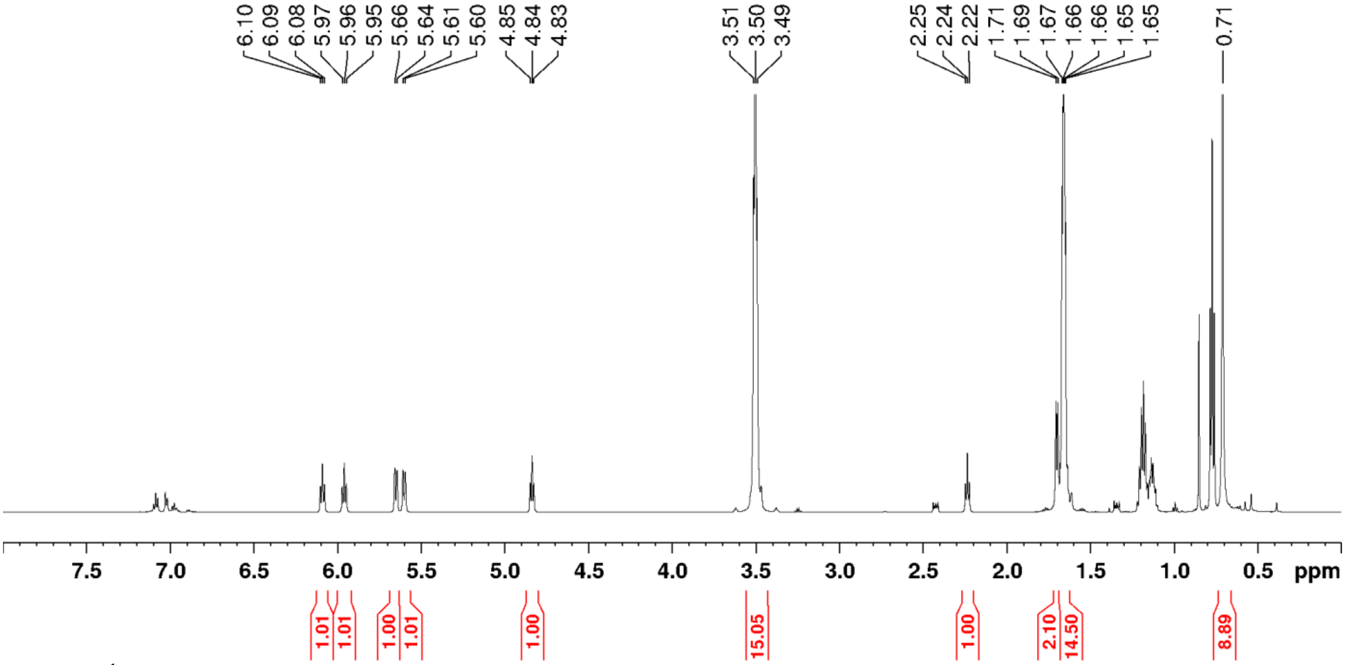

Figure S33: ^{1}H-NMr spectrum of **4** at –30°C.

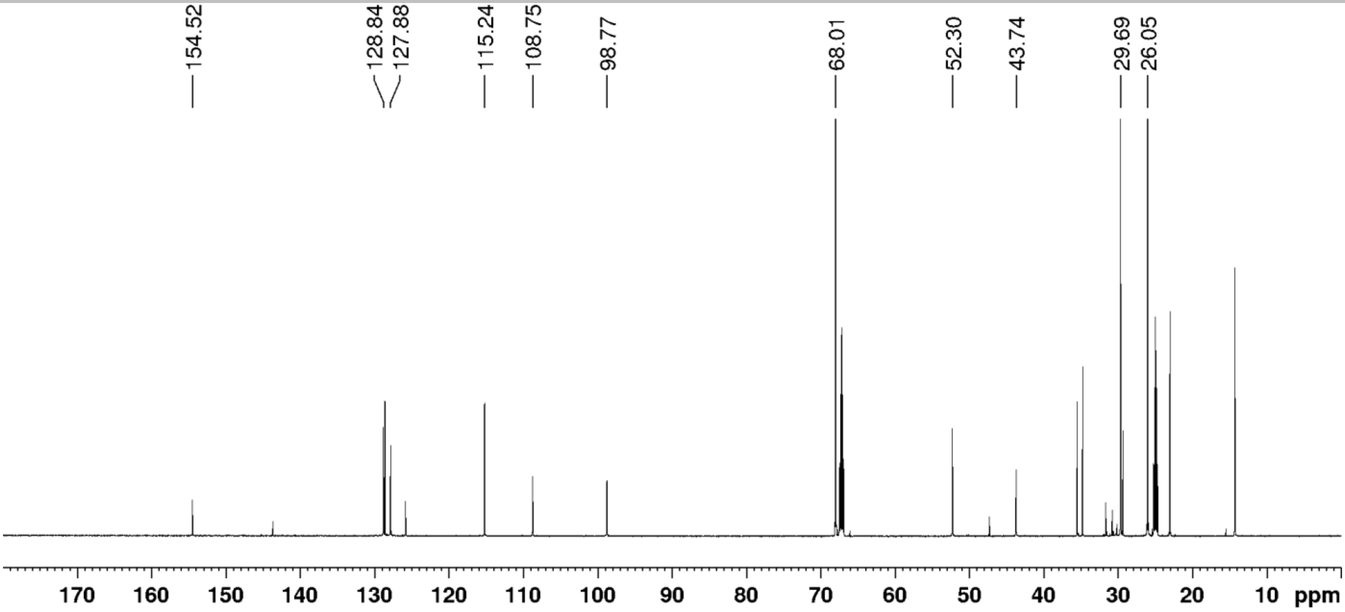

Figure S34: ^{13}C-NMR spectrum of **4** at –30 °C.

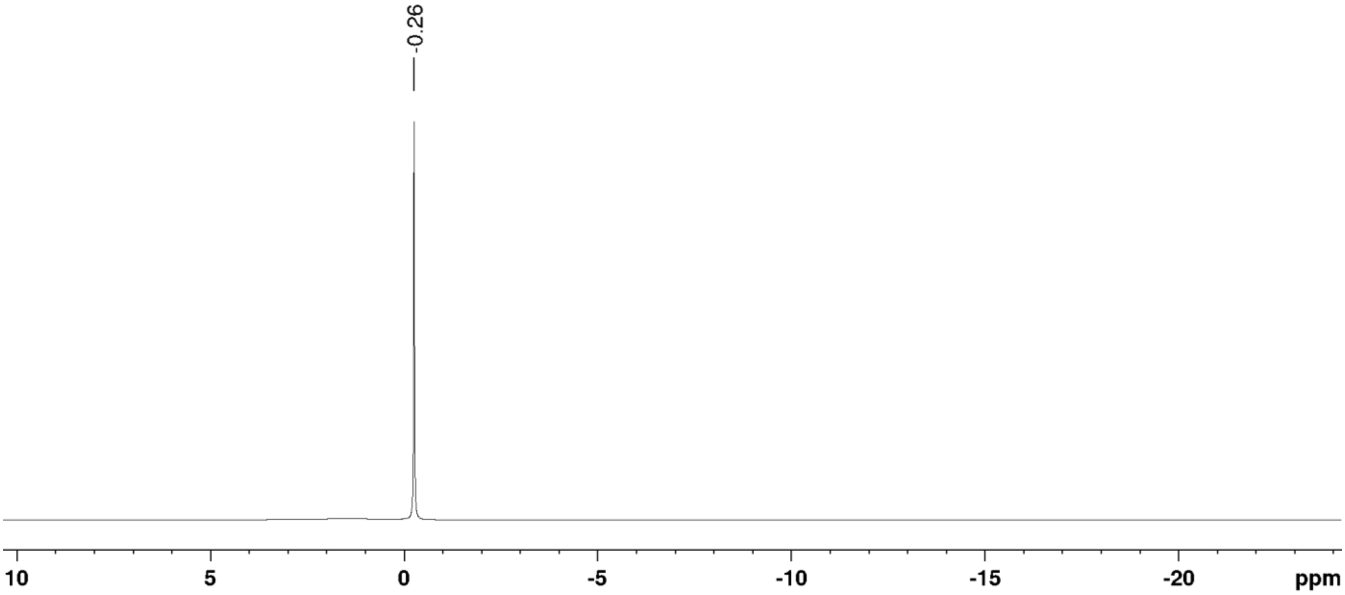

Figure S35: ^{7}Li-NMR spectrum of **4**.

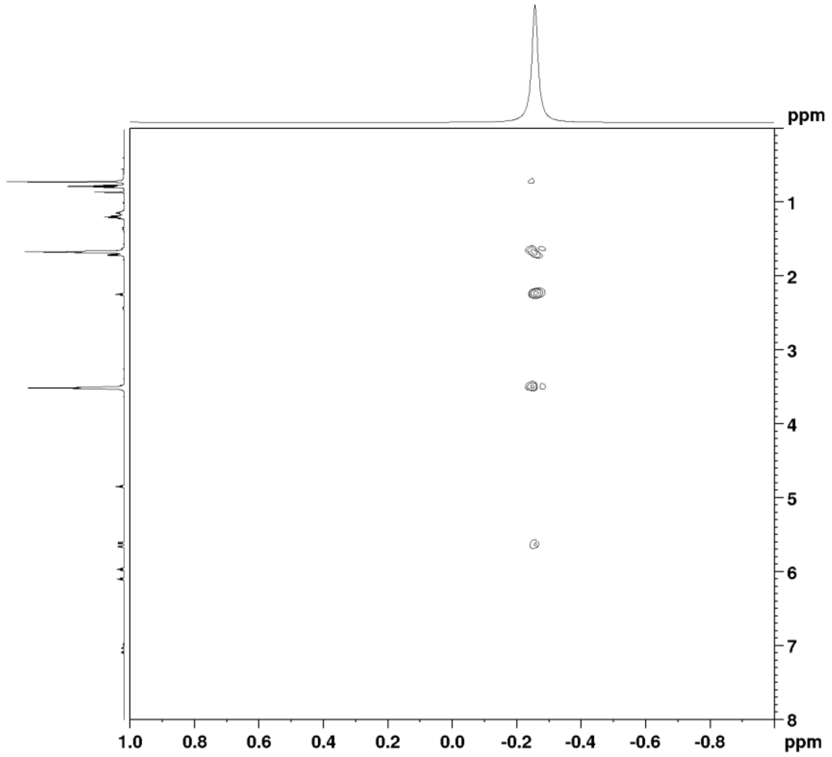

Figure S36: ^{1}H-^{7}Li-HOESY NMR spectrum of **4** at –30 °C:

Table S2: Comparison of NMR characteristics of **3** and **4**, obtained from experimental and calculated data.

	3	4
$\delta(C_{carb})$ ^{1}H [ppm]	2.76	2.51
$\delta(C_{carb})$ ^{13}C [ppm]	59.5	53.9
Diffusion coefficient (^{1}H-DOSY) [m^2/s][a]	$5.27 \cdot 10^{-10}$	$6.12 \cdot 10^{-10}$
Diffusion coefficient (^{7}Li-DOSY) [m^2/s][a]	$5.55 \cdot 10^{-10}$	$6.23 \cdot 10^{-10}$
Relative contact-integral of carbanion (^{1}H-^{7}Li-HOESY)[b]	0.51	0.18

[a] determined from t1 relaxation curves; for ^{1}H diffusion coefficients, the mean value from all proton signals is given [b] calculated from ratio of HOESY integral = carbanionic proton contact to lithium cation : sum of both ether proton contacts to lithium cation.

Quantum chemical calculations

Mechanism calculations

As method and basis set for our DFT calculations, we chose M062X/6-311+G(d), as Minnesota Functionals have already been benchmarked for lithium alkyl compounds regarding the calculation of energies.[11] However, one is aware of the fact that quantum chemical calculations in the gas phase may not be fully in accordance with solid-state structural data.[12] In a first instance, one can assume that solid-state specific influences are not relevant for the regarded energies and a possible error in the calculated energies is comparable for ground as well as for transition states.

Additional calculations on the mechanism hypothesis presented in the manuscript were performed with different ligands, pcm-based solvent models and at different temperatures. The following table gives an overview of these results. In the cases, when **TS1-m$_L$** could not be obtained, **TS2-m$_L$** and **H** were referenced on **F**.

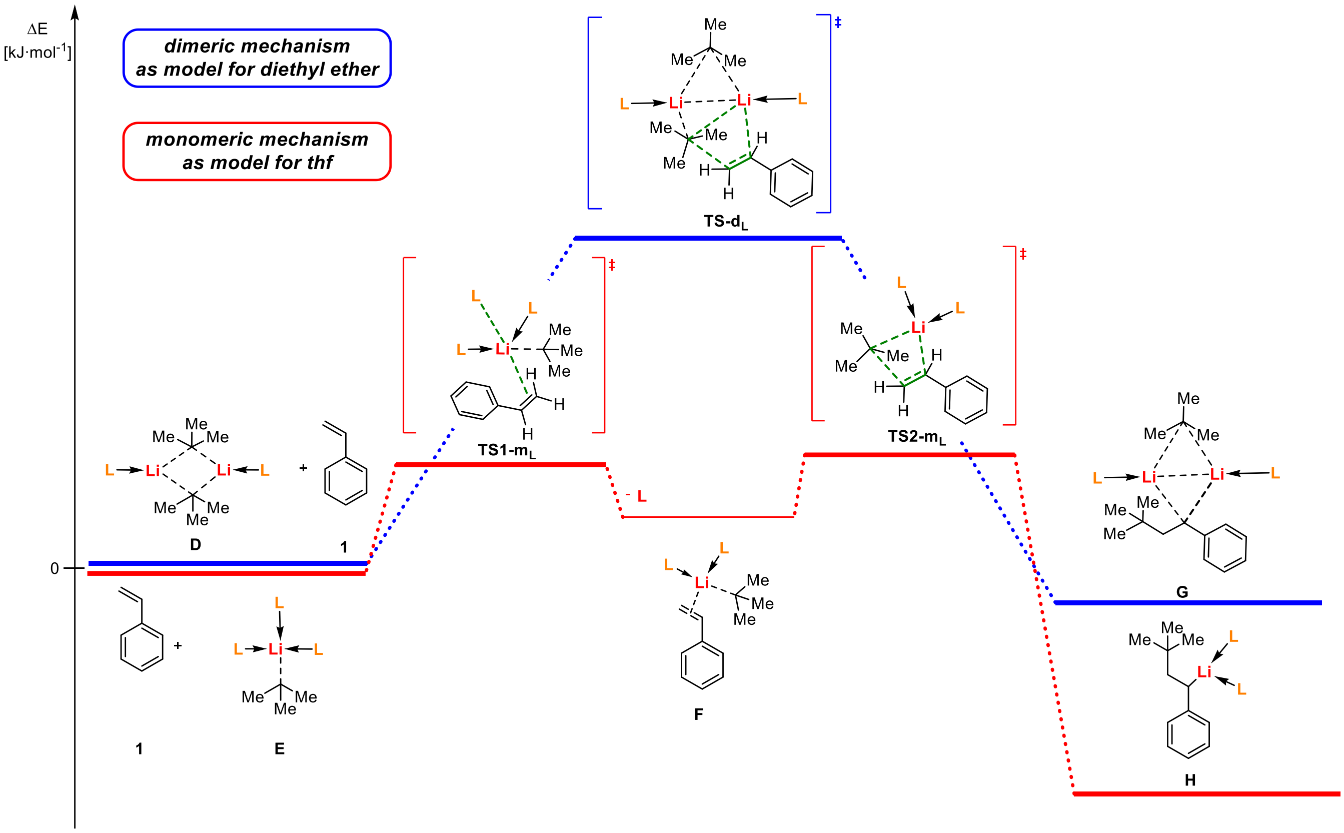

Figure S37: Mechanism hypothesis for addition of *tert*-butyllithium to styrene from dimeric and monomeric *tert*-butyllithium.

Table S3: Overview on results of DFT-calculations on mechanism hypothesis of addition of *tert*-butyllithium to styrene from dimeric and monomeric *tert*-butyllithium with variation on used ligands, temperature and solvent model (pcm); m062X/6-311+G(d).

ligand L	temperature [K]	solvent model		TS1-m$_L$	F	TS2-m$_L$	H	TS-d$_L$	G
DME	298	-	ΔG [kJ mol^{-1}]	22	16	21	-133	40	-148
			ΔH [kJ mol^{-1}]	27	11	27	-129	70	-124
DME	298	water	ΔG [kJ mol^{-1}]	-	0	12[a]	-151[a]	-	-
			ΔH [kJ mol^{-1}]	-	0	50[a]	-105[a]	-	-
DME	298	thf	ΔG [kJ mol^{-1}]	-	0	8[a]	-152[a]	-	-
			ΔH [kJ mol^{-1}]	-	0	20[a]	-139[a]	-	-
DME	193	-	ΔG [kJ mol^{-1}]	23	16	22	-132	41	-146
			ΔH [kJ mol^{-1}]	25	13	25	-131	60	-131
THF	298	-	ΔG [kJ mol^{-1}]	-	0	8[a]	-147[a]	-	-
			ΔH [kJ mol^{-1}]	-	0	20[a]	-129[a]	-	-
THF	193	-	ΔG [kJ mol^{-1}]	-	0	9[a]	-146[a]	-	-
			ΔH [kJ mol^{-1}]	-	0	16[a]	-135[a]	-	-
DME	298	Et_2O	ΔG [kJ mol^{-1}]	-	-	-	-	38	-153
			ΔH [kJ mol^{-1}]	-	-	-	-	49	-148
Et_2O	298	-	ΔG [kJ mol^{-1}]	-	-	-	-	62	-133
			ΔH [kJ mol^{-1}]	-	-	-	-	79	-118
Et_2O	193	-	ΔG [kJ mol^{-1}]	-	-	-	-	63	-131
			ΔH [kJ mol^{-1}]	-	-	-	-	73	-128

Values referenced to **1**+**D** or **1**+**E**. [a] Due to absence of TS1-m$_L$, values were referenced to **F**.

As can be seen, neither temperature, nor the solvent model or the ligands used have a noticeable effect on the transition state barriers. Only, diethyl ether as ligand results in higher energetic barrier of **TS-d$_L$** further strengthening our hypothesis.

Different "activation" modes for the monomeric *tert*-butyllithium in the context of carbolithiation to styrene were equally considered.

1) Direct carbolithiation of *tert*-butyllithium to styrene (m062X/6-311+G(d)/pcm=water)

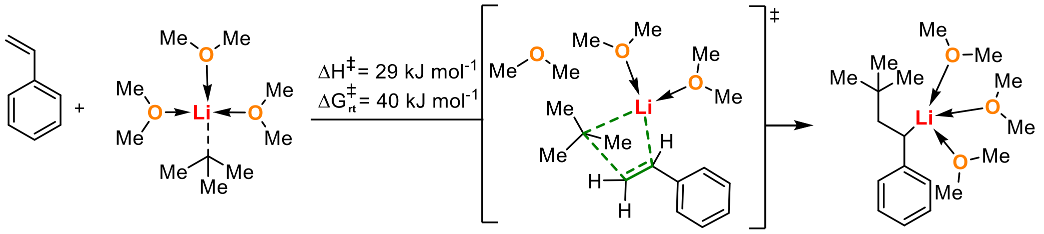

1 **7** **15** **16**

In the found transition state, one contact of dimethyl ether and lithium is already lost. Also, the energetic barrier of ΔH = 29 kJ mol^{-1} indicates no stabilizing effect of the additional dimethyl ether in the transition state.

2) Loss of dimethyl ether in first transition state without styrene coordination (m062X/6-311+G(d)/pcm=water)

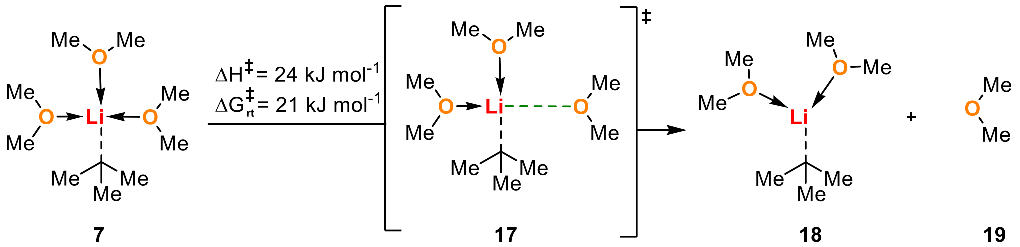

7 **17** **18** **19**

The activation barrier for the cleavage of one dimethyl ether is in the range as for a concerted exchange with styrene and may also be a probable option.

3) Bridged dimer as "activated" *tert*-butyllithium (m062X/6-311+G(d))

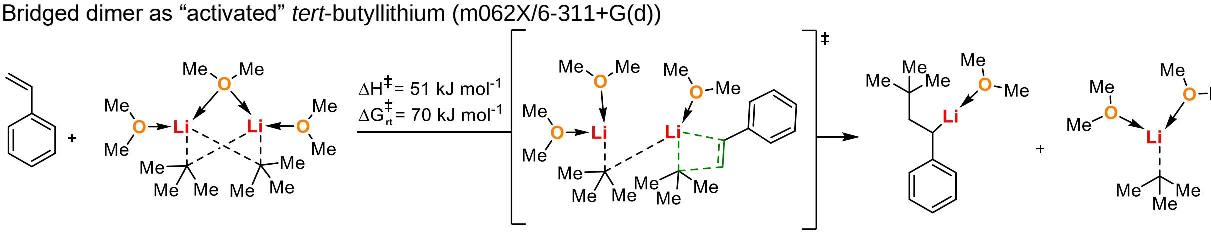

1 **20** **21** **30** **18**

Recently, we reported a dimeric under-saturated *tert*-butyllithium with bridged thf, that was more reactive than the monomeric form.[13] However, as the elevated energy barriers for addition of styrene to the bridged *tert*-butyllithium show, this structural motif may not be relevant for our investigations.

4) Direct carbolithiation of *iso*-propyllithium to styrene (m062X/6-311+G(d))

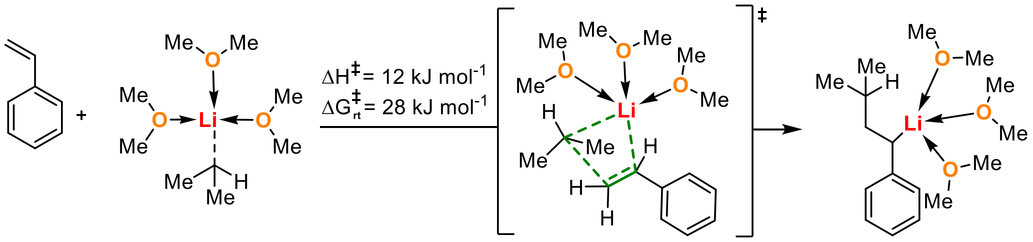

1 **29** **22** **23**

For comparison, the alkyl substituent was also varied. With a less sterically hindered lithium alkyl like *iso*-propyl lithium, the direct carbolithiation to styrene was possible without a loss of ligand in the transition state. This may be the reason for elevated reactivity of *sec*-butyllithium or *iso*-propyllithium in the initiation of anionic polymerization. However, the presence of a monomeric *iso*-propylithium or *sec*-butyllithium has not been reported so far.

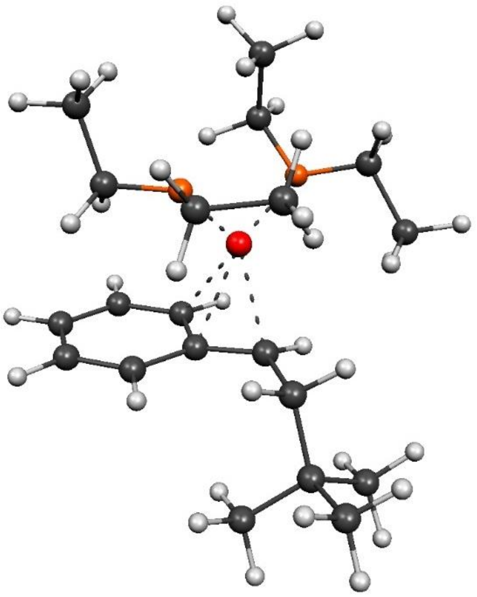

Figure S38: Molekel plot of **3**.

Table S4: Standard orientation of **3** [M062x/6-311+G(d)], T=298 K, E_{SCF} = – 942.189061521 H, E_{ZPE} = – 941.648480 H, no imaginary frequency.

Atomic symbol	x	y	z
O	1.956488	-1.663852	-1.037679
O	1.721257	0.443122	1.093062
C	-0.900949	1.012248	-0.773608
C	-0.034374	1.251903	-1.900126
H	0.117744	0.443166	-2.615484
C	-3.822249	-0.605283	0.306671
C	-1.113078	2.164046	0.061381
H	-1.776177	2.079321	0.915516
C	-0.556873	3.392843	-0.228728
H	-0.779643	4.231166	0.426346
C	1.811002	-3.077085	-0.937756
H	2.140972	-3.408102	0.05302
H	2.460672	-3.547536	-1.686562
C	0.269219	3.595577	-1.345439
H	0.682054	4.571763	-1.567609
C	-1.351608	-0.289614	-0.482303
C	-4.255555	0.816433	-0.054008
H	-4.118997	1.497149	0.792013
H	-5.314591	0.840419	-0.33043
H	-3.673519	1.203634	-0.893498
C	-2.303396	-0.649696	0.625657
H	-2.128464	-0.012734	1.504967
H	-2.095724	-1.674854	0.971604
C	3.304068	-1.209683	-1.152145
H	3.231275	-0.123492	-1.254538
H	3.724812	-1.60544	-2.08443
C	0.506812	2.503478	-2.174573
H	1.118173	2.623968	-3.064969
C	2.200255	1.79069	1.002165
H	1.517594	2.448442	1.547007
H	2.133803	2.076602	-0.049409
C	1.040047	0.157082	2.313118
H	0.09257	0.709422	2.31759
H	1.648645	0.505148	3.155465
C	-4.594089	-1.074754	1.542276
H	-4.378192	-0.437124	2.405547
H	-4.325234	-2.10184	1.809341
H	-5.674364	-1.046934	1.36974
C	-4.130388	-1.540792	-0.864584
H	-5.207097	-1.585872	-1.054827
H	-3.784227	-2.559219	-0.656173
H	-3.641073	-1.197278	-1.778803
C	0.361758	-3.444218	-1.169313
H	-0.284723	-2.984069	-0.419417
H	0.241107	-4.527791	-1.112191
H	0.030601	-3.108742	-2.153515
C	4.171395	-1.57597	0.039386
H	3.706345	-1.226852	0.962643
H	5.147305	-1.094796	-0.05816
H	4.343087	-2.651729	0.107564

C	0.805743	-1.336069	2.398347
H	0.267435	-1.581686	3.31563
H	1.753565	-1.879067	2.396559
H	0.197349	-1.674303	1.555747
C	3.622936	1.892227	1.52038
H	3.696078	1.569687	2.561854
H	3.972085	2.925437	1.463373
H	4.295682	1.272038	0.924019
Li	0.788674	-0.283676	-0.420808
H	-1.308968	-0.991214	-1.316965

Table S5: Standard orientation of **3** [M062x/6-311+G(d), scrf=(pcm,water)], T=298 K, E_{SCF} = – 941.989418043 H, E_{ZPE} = – 941.448431 H, no imaginary frequency.

Atomic symbol	x	y	z
O	1.582189	-1.60769	-1.103768
O	1.888905	0.356002	1.382167
C	-0.997301	1.094652	-0.516598
C	-0.02102	1.347561	-1.553498
H	0.238492	0.527466	-2.224469
C	-4.007387	-0.604671	0.268924
C	-1.330551	2.258494	0.271266
H	-2.077299	2.166344	1.055421
C	-0.754648	3.496257	0.040298
H	-1.058847	4.334924	0.665257
C	1.520705	-3.034556	-1.123291
H	2.333333	-3.44674	-0.511996
H	1.670356	-3.371762	-2.157536
C	0.202868	3.701571	-0.970193
H	0.646117	4.678017	-1.137368
C	-1.485151	-0.201418	-0.283924
C	-4.447267	0.790405	-0.1825
H	-4.409895	1.501982	0.651926
H	-5.476575	0.77072	-0.563182
H	-3.790503	1.164889	-0.975893
C	-2.525059	-0.581244	0.734617
H	-2.463773	0.086006	1.609247
H	-2.30817	-1.592179	1.120577
C	2.739061	-1.069748	-1.751518
H	2.537646	0.000565	-1.858043
H	2.806897	-1.503199	-2.758143
C	0.547725	2.598226	-1.760243
H	1.274542	2.713975	-2.563531
C	2.202456	1.753235	1.319206
H	2.25435	2.141994	2.344802
H	1.358509	2.226624	0.813413
C	2.574873	-0.347044	2.416433
H	2.335146	0.126788	3.377231
H	3.658617	-0.26854	2.259824
C	-4.884089	-1.059427	1.440227
H	-4.776973	-0.380307	2.295595
H	-4.603305	-2.067016	1.77218
H	-5.943931	-1.081004	1.157084
C	-4.170282	-1.590448	-0.892063
H	-5.221787	-1.665143	-1.196453
H	-3.828562	-2.593406	-0.603734
H	-3.585257	-1.27125	-1.761761
C	0.176243	-3.491675	-0.596194
H	0.010409	-3.138481	0.427458
H	0.144252	-4.585208	-0.58775
H	-0.638065	-3.124887	-1.227036
C	4.021282	-1.294335	-0.967282
H	3.917764	-0.897685	0.047434
H	4.844327	-0.76496	-1.457972
H	4.290711	-2.352715	-0.908994
C	2.131572	-1.794225	2.401465
H	2.62031	-2.344205	3.210298
H	2.395341	-2.272122	1.452474
H	1.047375	-1.864893	2.542397
C	3.49083	2.022116	0.560958
H	4.356258	1.543535	1.030125
H	3.6766	3.100654	0.530712
H	3.404904	1.663674	-0.470083
Li	0.653341	-0.39203	0.10071
H	-1.277243	-0.935219	-1.06539

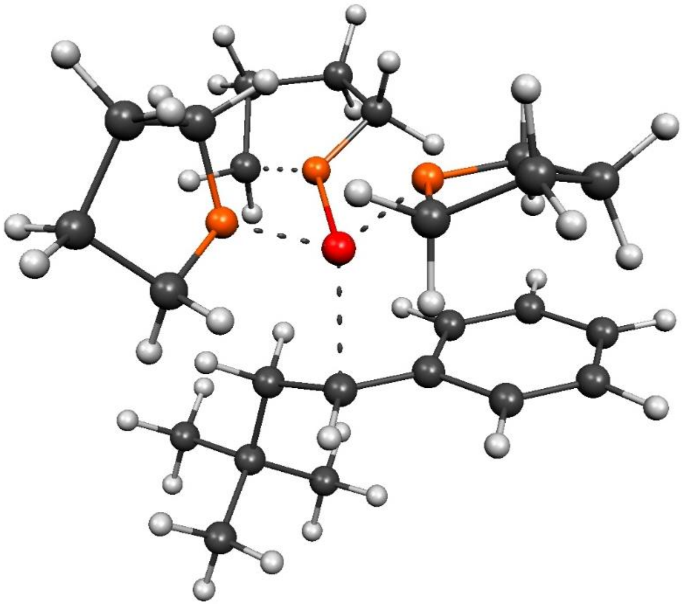

Figure S39: Molekel plot of **4**.

Table S6: Standard orientation of **4** [M062x/6-311+G(d)], T=298 K, E_{SCF} = – 1172.21111957 H, E_{ZPE} = – 1171.590871 H, no imaginary frequency.

Atomic symbol	x	y	z
O	-2.205563	-0.358208	-0.218472
O	-0.636092	2.074517	-0.70992
O	-0.299908	0.797536	1.82218
C	0.842305	-1.729638	-0.544239
C	2.28969	0.388977	-0.381036
H	2.1638	0.320884	0.707278
H	2.099272	1.449292	-0.618103
C	3.796733	0.141998	-0.667615
C	1.296605	-0.504597	-1.079595
C	-0.326085	-3.887172	-0.860167
H	-0.797674	-4.589205	-1.5439
C	0.952883	-2.106858	0.83835
H	1.488916	-1.460856	1.527019
C	0.159144	-2.696027	-1.360696
H	0.077543	-2.494478	-2.426743
C	-2.70191	-1.593636	0.34739
H	-3.347376	-1.322971	1.187599
H	-1.85779	-2.184932	0.704261
C	-0.213117	-4.21744	0.497707
H	-0.584782	-5.159444	0.881726
C	0.444562	-3.303612	1.323184
H	0.581002	-3.537261	2.376533
C	-0.277999	2.484878	-2.035287
H	-0.720463	1.78948	-2.757028
H	0.80688	2.429349	-2.122517
C	-2.15373	3.761795	-1.370481
H	-2.551888	4.719286	-1.03415
H	-2.923399	3.265447	-1.967939
C	-0.858371	3.885771	-2.178753
H	-1.021899	4.168658	-3.218725
H	-0.190851	4.620692	-1.721119
C	4.188412	-1.272454	-0.238263
H	4.038675	-1.413207	0.836738
H	5.242717	-1.468892	-0.459363
H	3.581615	-2.018571	-0.755823
C	-1.719375	2.868821	-0.207875
H	-1.348421	3.458578	0.637217
H	-2.499795	2.193925	0.150637
C	-2.542054	-0.297063	-1.609169
H	-1.704716	-0.693383	-2.196695
H	-2.698293	0.75309	-1.864724
C	4.077614	0.318824	-2.160591
H	3.536026	-0.425214	-2.748861
H	5.145167	0.207142	-2.374579
H	3.770061	1.313307	-2.503612
C	-3.786686	-1.160353	-1.747308
H	-3.945576	-1.50523	-2.769471

H	-4.669382	-0.598621	-1.429078
C	-3.477614	-2.297495	-0.768715
H	-4.372874	-2.795451	-0.395374
H	-2.838363	-3.042165	-1.243313
C	-1.114521	0.165034	2.822436
H	-0.943436	-0.912517	2.773719
H	-2.159955	0.373393	2.576229
C	4.615692	1.163815	0.124835
H	4.361066	2.187575	-0.16926
H	5.689478	1.027719	-0.037396
H	4.427354	1.06746	1.199513
C	0.538342	1.792851	2.413822
H	0.647628	2.60348	1.691843
H	1.527532	1.365379	2.612471
C	-0.702646	0.798384	4.152334
H	0.096423	0.217604	4.619519
H	-1.533013	0.862228	4.855511
C	-0.167976	2.164334	3.709482
H	-0.993645	2.852824	3.507677
H	0.500024	2.62542	4.437218
Li	-0.383007	0.278585	-0.009898
H	1.244552	-0.411163	-2.164051

Table S7: Standard orientation of **4** [M062x/6-311+G(d),scrf=(pcm,water)], T=298 K, E_{SCF} = – 1172.22678891 H, E_{ZPE} = – 1171.607709 H, no imaginary frequency.

Atomic symbol	x	y	z
O	-2.116315	-0.54731	-0.155009
O	-0.677552	1.903911	-0.909259
O	-0.306796	0.878471	1.796814
C	1.003119	-1.835831	-0.433335
C	2.276195	0.399273	-0.241298
H	2.129678	0.326629	0.844597
H	1.969356	1.425354	-0.501615
C	3.807067	0.327968	-0.493132
C	1.403532	-0.598469	-0.956725
C	-0.137719	-4.014707	-0.74374
H	-0.652211	-4.699008	-1.414844
C	1.207312	-2.253398	0.93162
H	1.751077	-1.603392	1.609206
C	0.299781	-2.804057	-1.239874
H	0.122739	-2.563519	-2.286276
C	-2.541056	-1.751891	0.520704
H	-3.134886	-1.4471	1.386382
H	-1.655097	-2.296265	0.853051
C	0.05909	-4.379998	0.598698
H	-0.288529	-5.331892	0.982657
C	0.743417	-3.46867	1.411722
H	0.929504	-3.718324	2.454051
C	-0.325183	2.204342	-2.268908
H	-0.744969	1.431576	-2.921675
H	0.762468	2.180993	-2.348309
C	-2.228745	3.494165	-1.71157
H	-2.6481	4.471342	-1.473637
H	-2.989836	2.912181	-2.23784
C	-0.946792	3.566483	-2.546244
H	-1.130227	3.730994	-3.607705
H	-0.295808	4.362766	-2.176721
C	4.353815	-1.022164	-0.027673
H	4.212679	-1.149658	1.050268
H	5.424965	-1.105514	-0.239507
H	3.8356	-1.842012	-0.53139
C	-1.750881	2.752928	-0.466125
H	-1.35527	3.44517	0.283076
H	-2.512937	2.123539	-0.000968
C	-2.612907	-0.541697	-1.501072
H	-1.836144	-0.932694	-2.16911
H	-2.83343	0.49161	-1.772039
C	4.100058	0.514784	-1.982472
H	3.653075	-0.289245	-2.572432
H	5.178109	0.517134	-2.172635
H	3.692698	1.465114	-2.344569
C	-3.83197	-1.449812	-1.46968
H	-4.089488	-1.836588	-2.4557
H	-4.692802	-0.90646	-1.071636
C	-3.374444	-2.536766	-0.492899
H	-4.202476	-3.065262	-0.020196
H	-2.744078	-3.263733	-1.007531
C	-1.367452	0.580448	2.723495
H	-1.312139	-0.47992	2.975633

H	-2.321635	0.778725	2.226029
C	4.481888	1.450854	0.298733
H	4.117875	2.431787	-0.023736
H	5.568218	1.436187	0.164587
H	4.274941	1.35261	1.369523
C	0.401448	2.060263	2.209197
H	0.595636	2.661817	1.319959
H	1.357704	1.760848	2.649756
C	-1.144477	1.509225	3.911505
H	-0.450034	1.057702	4.624157
H	-2.072921	1.741832	4.432301
C	-0.500008	2.724802	3.240623
H	-1.262283	3.333709	2.747382
H	0.056919	3.357617	3.93123
Li	-0.370582	0.24423	-0.005732
H	1.25858	-0.448364	-2.026233

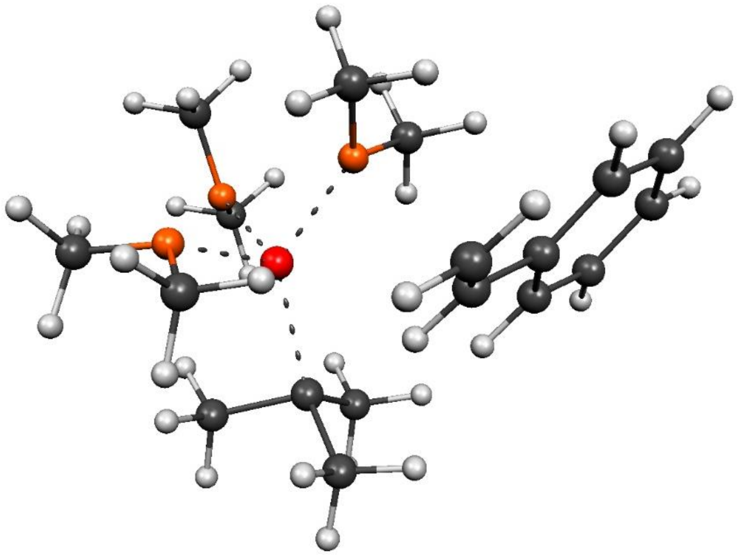

Figure S40: Molekel plot of **1+7**.

Table S8: Standard orientation of **1+7** [M062x/6-311+G(d)], T=298 K, E_{SCF} = –939.910579896 H, E_{ZPE} = –939.405429 H, no imaginary frequency.

Atomic symbol	x	y	z
C	-1.21068	1.511423	-1.497927
C	-0.62416	1.240407	-2.881962
H	0.469621	1.324214	-2.913684
H	-0.874367	0.239896	-3.261159
H	-1.000747	1.960975	-3.639228
C	-0.824486	2.944005	-1.130779
H	-1.153645	3.674671	-1.901015
H	-1.266406	3.282768	-0.184981
H	0.259954	3.079683	-1.028332
C	-2.732827	1.490719	-1.679991
H	-3.076886	0.542545	-2.114376
H	-3.294886	1.630076	-0.744329
H	-3.084135	2.285225	-2.372905
Li	-1.283512	0.090312	0.052545
O	-2.33785	-1.552804	-0.363627
O	0.126082	-0.734733	1.166293
O	-2.379811	0.526066	1.658543
C	-1.835668	-2.199649	-1.519057
H	-2.154431	-3.248075	-1.539647
H	-2.183956	-1.690766	-2.423419
H	-0.748892	-2.148591	-1.473568
C	-3.751145	-1.589487	-0.306249
H	-4.063437	-0.947123	0.514592
H	-4.186457	-1.21115	-1.235547
H	-4.096219	-2.615173	-0.134492
C	0.747901	0.198085	2.02998
H	0.50282	1.19436	1.660624
H	0.373819	0.075104	3.053977
H	1.834773	0.069734	2.02247
C	0.452647	-2.0697	1.47634
H	0.180596	-2.301756	2.514087
H	-0.11195	-2.711076	0.800916

H	1.524817	-2.244043	1.335454
C	-2.702765	1.819184	2.128889
H	-2.578718	2.506555	1.295776
H	-3.73917	1.851555	2.482285
H	-2.033886	2.107542	2.947964
C	-2.522839	-0.469688	2.647577
H	-3.559831	-0.515466	3.000668
H	-2.246587	-1.420579	2.193805
H	-1.869168	-0.266926	3.503666
C	2.418482	0.991404	-0.565832
C	3.20777	1.8415	0.20074
C	4.279623	1.332139	0.927013
C	4.556432	-0.033726	0.879342
C	3.774052	-0.880081	0.103247
C	2.697607	-0.37671	-0.637155
H	1.551976	1.374031	-1.094313
H	2.975289	2.900408	0.238014
H	4.895387	1.990618	1.529569
H	5.386291	-0.43895	1.448044
H	4.003577	-1.94045	0.078977
C	1.828269	-1.218633	-1.477965
H	0.950388	-0.702884	-1.863841
C	2.034089	-2.495437	-1.801415
H	2.902903	-3.053901	-1.467866
H	1.340925	-3.026748	-2.444259

Table S9: Standard orientation of **1+7** [M062x/6-311+G(d)], T=193 K, E_{SCF} = – 939.910596774 H, E_{ZPE} = –939.405506 H, no imaginary frequency.

Atomic symbol	x	y	z
C	-1.208876	1.453118	-1.554413
C	-0.622095	1.130403	-2.92722
H	0.471722	1.212987	-2.961773
H	-0.872156	0.116352	-3.268675
H	-0.998528	1.822064	-3.711025
C	-0.822628	2.898347	-1.241045
H	-1.15124	3.59968	-2.038297
H	-1.265558	3.271993	-0.308958
H	0.261772	3.037883	-1.142912
C	-2.731052	1.425902	-1.735558
H	-3.075176	0.462597	-2.135164
H	-3.293017	1.599276	-0.805511
H	-3.082288	2.194641	-2.45696
Li	-1.282191	0.090163	0.04733
O	-2.338403	-1.565057	-0.306784
O	0.124248	-0.689798	1.194892
O	-2.381036	0.5885	1.636594
C	-1.834935	-2.257721	-1.434811
H	-2.153461	-3.306194	-1.413326
H	-2.18263	-1.785924	-2.359295
H	-0.748162	-2.204852	-1.390839
C	-3.751775	-1.599454	-0.249787
H	-4.065035	-0.925868	0.545245
H	-4.185863	-1.257294	-1.193548
H	-4.097101	-2.617737	-0.038929
C	0.744754	0.277491	2.020862
H	0.499767	1.257882	1.611178
H	0.369499	0.195962	3.048545
H	1.831677	0.149304	2.019683
C	0.450366	-2.011079	1.559055
H	0.176356	-2.201314	2.604764
H	-0.112824	-2.679222	0.90886
H	1.522842	-2.190721	1.427331
C	-2.704117	1.898524	2.057366
H	-2.578461	2.553974	1.199128
H	-3.740974	1.944718	2.407915
H	-2.036197	2.217336	2.865895
C	-2.527784	-0.369354	2.661757
H	-3.565821	-0.401183	3.013344
H	-2.251015	-1.336809	2.244776
H	-1.876501	-0.135196	3.511662
C	2.418966	0.969144	-0.60062
C	3.207735	1.846992	0.134566
C	4.279549	1.364918	0.879332
C	4.556968	-0.001627	0.881646
C	3.775148	-0.876117	0.136778
C	2.69877	-0.400578	-0.621843
H	1.552714	1.332061	-1.143351
H	2.974903	2.906482	0.133015
H	4.894836	2.045195	1.457676
H	5.386855	-0.385417	1.464995
H	4.00502	-1.936597	0.151335

C	1.830132	-1.273082	-1.431646
H	0.950506	-0.772763	-1.833553
C	2.0386	-2.559824	-1.710951
H	2.909463	-3.104117	-1.359774
H	1.345818	-3.114843	-2.333873

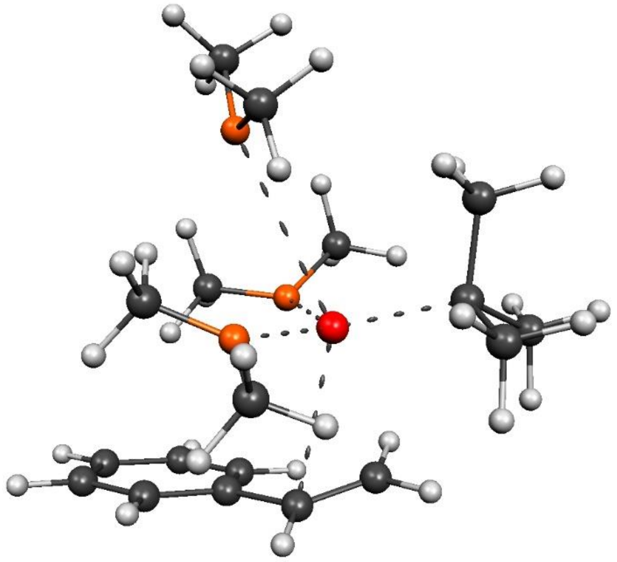

Figure S41: Molekel plot of **TS1-m$_{DME}$**.

Table S10: Standard orientation of **TS1-m$_{DME}$** [M062x/6-311+G(d)], T=298 K, E_{SCF} = –939.901415660H, E_{ZPE} = – 939.396383 H, one imaginary frequency**.**

Atomic symbol	x	y	z
C	-2.447438	1.780346	-0.416034
C	-2.372102	2.685148	-1.645412
H	-1.593863	3.45439	-1.569792
H	-2.182245	2.134588	-2.57694
H	-3.324722	3.23136	-1.808445
C	-2.809974	2.678266	0.767477
H	-3.740713	3.256251	0.584693
H	-2.983728	2.104509	1.688031
H	-2.033367	3.423206	0.993287
C	-3.645517	0.851818	-0.630579
H	-3.487046	0.112033	-1.428148
H	-3.898104	0.294366	0.277735
H	-4.561644	1.416073	-0.907189
Li	-0.738461	0.593003	0.012091
O	0.080998	-0.56481	-1.29503
O	-0.300845	-0.068327	1.787041
O	-1.929264	-2.603694	0.055297
C	0.924929	-1.68148	-1.091963
H	0.324821	-2.570748	-0.876515
H	1.542325	-1.854294	-1.979441
H	1.577545	-1.45862	-0.248778
C	-0.772654	-0.7386	-2.414858
H	-1.377261	0.161638	-2.502271
H	-0.175031	-0.881819	-3.321823
H	-1.42721	-1.601189	-2.259011
C	-0.403532	0.895634	2.818013
H	-0.695288	1.838098	2.356706
H	-1.168267	0.596758	3.542841
H	0.557223	1.010216	3.333634
C	0.083201	-1.344595	2.260996
H	-0.562324	-1.650253	3.092284
H	-0.03711	-2.04868	1.440588
H	1.124898	-1.328989	2.599723
C	-2.868294	-2.188531	1.023239
H	-2.520057	-1.240547	1.43249
H	-3.852924	-2.032134	0.57084
H	-2.955069	-2.934744	1.823987
C	-2.369857	-3.739848	-0.647046
H	-3.290423	-3.525068	-1.203313

H	-1.586271	-4.022352	-1.351
H	-2.556812	-4.580211	0.033135
C	3.180146	0.303447	1.151304
C	4.093993	-0.707953	0.87385
C	4.484193	-0.949861	-0.439695
C	3.958624	-0.168618	-1.467592
C	3.03869	0.832732	-1.189121
C	2.626899	1.078109	0.126323
H	2.886205	0.500451	2.17863
H	4.507931	-1.300191	1.682656
H	5.203685	-1.730116	-0.660626
H	4.272872	-0.339618	-2.491547
H	2.644874	1.441983	-1.995103
C	1.64055	2.116983	0.46281
H	1.660614	2.456786	1.496469
C	0.731694	2.639203	-0.363981
H	0.632302	2.326345	-1.399366
H	0.041153	3.402961	-0.029548

Table S11: Standard orientation of **TS1-m$_{DME}$** [M062x/6-311+G(d)], T=193 K, E_{SCF} = – 939.901415667 H, E_{ZPE} = –939.396385 H, one imaginary frequency**.**

Atomic symbol	x	y	z
C	2.447676	-1.780466	-0.416343
C	2.372467	-2.684585	-1.646231
H	1.594367	-3.454012	-1.570999
H	2.18248	-2.133559	-2.577458
H	3.325169	-3.230557	-1.809595
C	2.810054	-2.679074	0.766698
H	3.740896	-3.256844	0.583739
H	2.983583	-2.105868	1.687638
H	2.033515	-3.424268	0.991929
C	3.64583	-0.851877	-0.630139
H	3.48763	-0.111712	-1.427376
H	3.898238	-0.294967	0.278597
H	4.562006	-1.416029	-0.906795
Li	0.738372	-0.593684	0.01191
O	-0.080665	0.565066	-1.294724
O	0.30081	0.067138	1.787164
O	1.928831	2.60473	0.055662
C	-0.924709	1.68162	-1.091208
H	-0.324658	2.57083	-0.875356
H	-1.542068	1.854757	-1.978648
H	-1.577361	1.458411	-0.248148
C	0.773404	0.739695	-2.414149
H	1.377647	-0.160712	-2.502376
H	0.176128	0.884146	-3.321142
H	1.428269	1.601803	-2.257058
C	0.403218	-0.89728	2.817734
H	0.694953	-1.839578	2.356072
H	1.167866	-0.598803	3.542814
H	-0.557636	-1.011984	3.333145
C	-0.082901	1.343329	2.261626
H	0.562789	1.648527	3.092955
H	0.037497	2.047712	1.441474
H	-1.12456	1.327845	2.600473
C	2.867989	2.188576	1.023035
H	2.519658	1.240334	1.431662
H	3.852455	2.032283	0.570243
H	2.955153	2.934129	1.824358
C	2.36917	3.741674	-0.645559
H	3.290015	3.527754	-1.2017
H	1.585652	4.024307	-1.34952
H	2.555514	4.581575	0.035353
C	-3.180493	-0.303991	1.151053
C	-4.09422	0.707591	0.873887
C	-4.484144	0.95013	-0.439627
C	-3.95847	0.169296	-1.467779
C	-3.038684	-0.832266	-1.189588
C	-2.627123	-1.078216	0.125813
H	-2.886728	-0.501458	2.17834
H	-4.508239	1.299517	1.68288
H	-5.203525	1.730549	-0.660338
H	-4.272516	0.340781	-2.491715
H	-2.64474	-1.441182	-1.995763
C	-1.640844	-2.117254	0.46197
H	-1.661355	-2.457799	1.495378
C	-0.731819	-2.63894	-0.364959
H	-0.632156	-2.325267	-1.400079
H	-0.041258	-3.402851	-0.030933

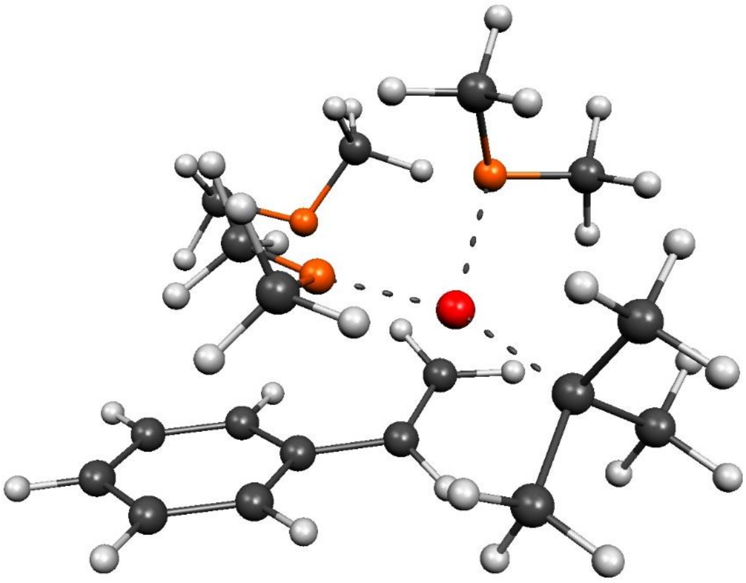

Figure S42: Molekel plot of **8** and dimethyl ether (**20**).

Table S12: Standard orientation of **8** and dimethyl ether (**19**) [M062x/6-311+G(d)], T=298 K, E_{SCF} = – 939.904109248 H, E_{ZPE} = – 939.399678 H, no imaginary frequency**.**

Atomic symbol	x	y	z
C	-3.008353	1.087384	-0.350243
C	-3.504563	0.810518	-1.770936
H	-2.754332	1.055947	-2.536907
H	-3.785503	-0.239488	-1.927685
H	-4.405111	1.4089	-2.027043
C	-2.773102	2.595787	-0.265039
H	-3.694885	3.173998	-0.489258
H	-2.443698	2.926267	0.729549
H	-2.020004	2.949759	-0.982893
C	-4.164018	0.769536	0.598434
H	-4.477764	-0.282462	0.546774
H	-3.916844	0.974803	1.649626
H	-5.07269	1.368386	0.372931
Li	-1.322796	-0.081882	-0.002273
O	-1.580583	-2.02405	0.289272
O	2.254182	-2.52991	-0.646169
O	-0.171955	0.244	1.485873
C	-2.518473	-2.557692	-0.630511
H	-2.590716	-3.644064	-0.508337
H	-3.501755	-2.101739	-0.485055
H	-2.164808	-2.327034	-1.634082
C	-1.988525	-2.240095	1.630042
H	-1.182894	-1.898783	2.276668
H	-2.898227	-1.670447	1.848366
H	-2.171558	-3.306231	1.801892
C	3.423175	-2.566641	0.134567
H	4.077974	-1.769382	-0.21432
H	3.194947	-2.40228	1.196256
H	3.936938	-3.529979	0.031776
C	1.325825	-3.517006	-0.260584
H	1.018661	-3.381949	0.784714
H	0.446897	-3.408536	-0.89406
H	1.749212	-4.521208	-0.382521
C	-0.529138	1.180253	2.482782
H	-1.528278	1.53756	2.239299
H	-0.532033	0.70318	3.470003
H	0.176976	2.017662	2.487161
C	1.10715	-0.325633	1.674428
H	1.12722	-0.919907	2.597611
H	1.305462	-0.962697	0.812163
H	1.874378	0.454476	1.72921
C	1.254367	2.512888	-0.224374
C	2.324935	3.031319	0.496128
C	3.577096	2.427744	0.414869
C	3.750914	1.305322	-0.392572
C	2.679679	0.782729	-1.107057
C	1.416293	1.377856	-1.025155

H	0.272769	2.973901	-0.155705
H	2.183696	3.910284	1.115711
H	4.416087	2.836533	0.966815
H	4.730276	0.845703	-0.47391
H	2.819913	-0.088316	-1.738709
C	0.243914	0.833034	-1.733273
H	-0.569939	1.533344	-1.908429
C	0.096385	-0.435529	-2.125266
H	0.857191	-1.189531	-1.944945
H	-0.803689	-0.731976	-2.655092

Table S13: Standard orientation of **8** and dimethyl ether (**19**) [M062x/6-311+G(d)], T=193 K, E_{SCF} = – 939.904109258 H, E_{ZPE} = – 939.399677 H, no imaginary frequency**.**

Atomic symbol	x	y	z
C	3.008372	-1.087219	-0.350289
C	3.50462	-0.810189	-1.77093
H	2.754431	-1.055653	-2.53693
H	3.78545	0.239857	-1.927604
H	4.405242	-1.408457	-2.027055
C	2.773261	-2.595645	-0.26522
H	3.695101	-3.173766	-0.489447
H	2.443837	-2.926217	0.729331
H	2.020216	-2.949615	-0.983123
C	4.163933	-0.769327	0.5985
H	4.477584	0.282703	0.546949
H	3.916706	-0.974698	1.64966
H	5.072684	-1.36807	0.373016
Li	1.322663	0.081781	-0.002315
O	1.580112	2.024159	0.288903
O	-2.254178	2.529814	-0.646211
O	0.171957	-0.244065	1.485928
C	2.518198	2.557874	-0.630636
H	2.590158	3.644278	-0.508584
H	3.501536	2.102155	-0.484799
H	2.164941	2.326969	-1.634291
C	1.987642	2.240446	1.629768
H	1.18188	1.899102	2.276213
H	2.897359	1.670957	1.848429
H	2.170465	3.306634	1.801513
C	-3.423274	2.566375	0.134372
H	-4.077894	1.76907	-0.214752
H	-3.195215	2.401921	1.196089
H	-3.937123	3.529666	0.031591
C	-1.325932	3.51695	-0.260471
H	-1.018955	3.38192	0.784892
H	-0.446885	3.408479	-0.893778
H	-1.749344	4.52113	-0.382485
C	0.529182	-1.180313	2.482825
H	1.528229	-1.537742	2.239174
H	0.53229	-0.703207	3.470028
H	-0.177025	-2.017645	2.487339
C	-1.107136	0.325568	1.674527
H	-1.127188	0.919839	2.597713
H	-1.305446	0.962622	0.812249
H	-1.874352	-0.454552	1.729323
C	-1.254051	-2.513112	-0.22452
C	-2.324523	-3.031669	0.49603
C	-3.576689	-2.428065	0.415059
C	-3.750622	-1.305499	-0.392154
C	-2.679483	-0.782775	-1.106697
C	-1.416079	-1.377915	-1.025054
H	-0.272468	-2.974215	-0.156159
H	-2.183216	-3.910756	1.115422
H	-4.415599	-2.836939	0.967065
H	-4.729998	-0.845846	-0.473189
H	-2.819796	0.0884	-1.738151
C	-0.243757	-0.83297	-1.733173
H	0.570139	-1.533222	-1.908401
C	-0.096261	0.435601	-2.125166
H	-0.857073	1.189612	-1.944867
H	0.80382	0.731991	-2.655016

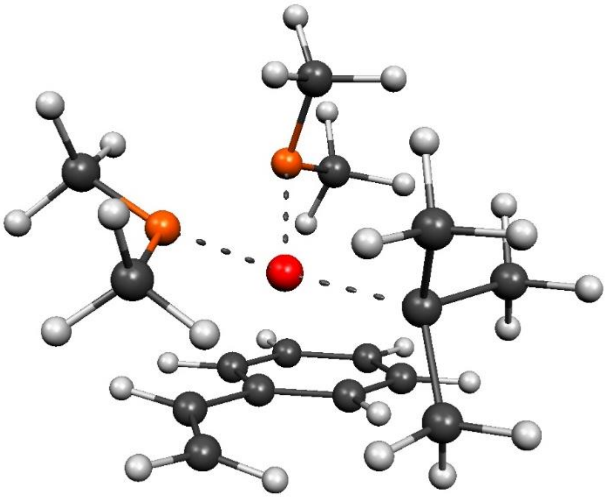

Figure S43: Molekel plot of **8**.

Table S14: Standard orientation of **8** [M062x/6-311+G(d)], T=298 K, E_{SCF} = – 784.904285492 H, E_{ZPE} = – 784.481760 H, no imaginary frequency

Atomic symbol	x	y	z
C	1.499385	-1.823121	0.270684
C	1.560948	-2.37444	1.692842
H	2.174816	-1.754229	2.3611
H	0.570807	-2.464994	2.163636
H	1.999732	-3.393353	1.726666
C	2.9284	-1.87384	-0.278843
H	3.345873	-2.902299	-0.245936
H	2.994042	-1.555364	-1.32685
H	3.62999	-1.247374	0.285951
C	0.687869	-2.821284	-0.558146
H	1.117066	-3.844617	-0.514045
H	-0.353349	-2.912803	-0.22271
H	0.653291	-2.555144	-1.623303
Li	0.820348	0.144366	-0.049315
O	2.154965	1.560112	0.074882
O	0.216067	0.624244	-1.860987
C	3.135785	1.443557	1.087866
H	4.139439	1.535521	0.66033
H	2.993939	2.219621	1.848531
H	3.016357	0.458949	1.537511
C	2.088882	2.850597	-0.492363
H	1.833983	3.594255	0.271275
H	3.047675	3.121569	-0.948189
H	1.310999	2.828725	-1.2542
C	-1.023493	0.144318	-2.36228
H	-1.813025	0.52651	-1.719739
H	-1.176442	0.50576	-3.384596
H	-1.038856	-0.949851	-2.351275
C	1.289571	0.303831	-2.732937
H	2.211495	0.633905	-2.256375
H	1.340595	-0.77626	-2.897142
H	1.158418	0.816552	-3.69183
C	-1.921955	0.643889	1.046185
C	-3.005796	1.372075	0.54075
C	-4.046798	0.733883	-0.122889
C	-4.012236	-0.646532	-0.305908
C	-2.933091	-1.379773	0.180819
C	-1.896162	-0.744227	0.853531
H	-3.032086	2.44878	0.678597
H	-4.882754	1.312651	-0.499584
H	-4.820525	-1.147135	-0.826974
H	-2.892478	-2.453162	0.031957
H	-1.052177	-1.327193	1.205776
C	-0.837127	1.355105	1.744292
H	-0.853419	2.439372	1.649904
C	0.129998	0.786303	2.469341
H	0.887352	1.395439	2.949498
H	0.194857	-0.283818	2.631133

Table S15: Standard orientation of **8** [M062x/6-311+G(d)], T=193 K, E_{SCF} = – 784.904285570 H, E_{ZPE} = – 784.481764 H, no imaginary frequency

Atomic symbol	x	y	z
C	1.499564	1.823348	-0.271439

C	1.561345	2.373323	-1.694061
H	2.175595	1.752577	-2.361464
H	0.571262	2.462407	-2.16527
H	1.999455	3.392452	-1.728901
C	2.928255	1.875223	0.278749
H	3.345398	2.903786	0.244796
H	2.99336	1.558077	1.327181
H	3.63036	1.248286	-0.28489
C	0.687274	2.821983	0.556067
H	1.116462	3.845313	0.511594
H	-0.353628	2.913237	0.219668
H	0.651883	2.556561	1.621385
Li	0.820535	-0.144173	0.049778
O	2.154709	-1.56046	-0.073783
O	0.215772	-0.623273	1.861209
C	3.13632	-1.444632	-1.086103
H	4.139597	-1.537488	-0.657878
H	2.994361	-2.220517	-1.84692
H	3.017975	-0.459892	-1.535737
C	2.087214	-2.850881	0.493463
H	1.832465	-3.594381	-0.270376
H	3.045419	-3.122496	0.950147
H	1.308683	-2.828368	1.254621
C	-1.02397	-0.143365	2.362023
H	-1.813297	-0.526105	1.719556
H	-1.177031	-0.504309	3.3845
H	-1.039632	0.950794	2.350437
C	1.289093	-0.302227	2.73318
H	2.211146	-0.632234	2.25682
H	1.339754	0.777944	2.896989
H	1.157953	-0.814635	3.69224
C	-1.9219	-0.644433	-1.046376
C	-3.005753	-1.371979	-0.540041
C	-4.046339	-0.733054	0.123539
C	-4.011331	0.647473	0.305641
C	-2.932156	1.380081	-0.181984
C	-1.895672	0.743777	-0.854674
H	-3.032347	-2.448791	-0.67703
H	-4.882291	-1.311358	0.50096
H	-4.819281	1.148625	0.826703
H	-2.89114	2.453551	-0.033823
H	-1.051619	1.326226	-1.207537
C	-0.837392	-1.356551	-1.744006
H	-0.854757	-2.440784	-1.649335
C	0.1309	-0.789216	-2.468671
H	0.887626	-1.399992	-2.947811
H	0.198103	0.280684	-2.631214

Table S16: Standard orientation of **8** [M062x/6-311+G(d)/scrf=(pcm,water)], T=298 K, E_{SCF} = – 784.917425477 H, E_{ZPE} = –784.496369 H, no imaginary frequency

Atomic symbol	x	y	z
C	2.15142	0.754473	1.000842
C	2.197822	2.219685	1.403603
H	2.157139	2.902437	0.544298
H	1.369632	2.497974	2.07034
H	3.133772	2.472895	1.956083
C	3.383488	0.464749	0.150041
H	4.333945	0.71594	0.679205
H	3.458925	-0.596524	-0.124784
H	3.394559	1.036785	-0.787844
C	2.27995	-0.065266	2.278845
H	3.214784	0.173211	2.840317
H	1.452066	0.116138	2.978231
H	2.305982	-1.146674	2.088689
Li	0.645646	-0.110738	-0.171439
O	-0.008693	0.734698	-1.751295
O	0.976137	-1.898351	-0.892798
C	0.552133	1.947289	-2.221835
H	0.992551	1.799651	-3.213514
H	-0.214323	2.726361	-2.27476
H	1.32408	2.238994	-1.511229
C	-1.097076	0.288455	-2.537467
H	-1.932616	0.992302	-2.461372
H	-0.797005	0.19095	-3.586203
H	-1.403055	-0.683053	-2.15094
C	1.35705	-2.971372	-0.048201
H	0.848378	-2.83925	0.904384
H	1.060577	-3.92687	-0.491497
H	2.440281	-2.965462	0.11135
C	1.581865	-1.996279	-2.172229

H	1.277292	-1.125215	-2.748126
H	2.672321	-2.008722	-2.077734
H	1.252643	-2.909481	-2.676828
C	-2.274811	0.627872	0.643756
C	-3.17296	-0.316238	0.134282
C	-3.058925	-1.66353	0.463724
C	-2.048892	-2.088607	1.322769
C	-1.164621	-1.153916	1.860038
C	-1.27817	0.193224	1.530085
H	-3.957657	0.009385	-0.542006
H	-3.75841	-2.380791	0.049189
H	-1.957917	-3.137345	1.582863
H	-0.387976	-1.468859	2.549411
H	-0.591323	0.909696	1.968668
C	-2.385351	2.039696	0.233326
H	-3.363318	2.356006	-0.123518
C	-1.375051	2.909467	0.236515
H	-1.518851	3.934718	-0.086101

Table S17: Standard orientation of **8** [M062x/6-311+G(d)/scrf=(pcm,thf)], T=298 K, E_{SCF} = – 784.913769206 H, E_{ZPE} = – 784.492292 H, no imaginary frequency.

Atomic symbol	x	y	z
C	1.520306	1.846964	-0.25141
C	1.56617	2.450051	-1.649176
H	2.173164	1.854665	-2.344613
H	0.570753	2.552248	-2.104868
H	2.004016	3.474201	-1.648786
C	2.949059	1.86891	0.289987
H	3.371508	2.900085	0.301318
H	3.015916	1.501564	1.321898
H	3.642555	1.264012	-0.307077
C	0.709652	2.796874	0.626969
H	1.132752	3.828076	0.627944
H	-0.334863	2.893715	0.303886
H	0.686354	2.477251	1.67721
Li	0.850719	-0.137671	0.041882
O	2.157255	-1.566879	-0.105786
O	0.228151	-0.594144	1.848628
C	3.130815	-1.487065	-1.131146
H	4.132248	-1.645701	-0.719196
H	2.935834	-2.238151	-1.903673
H	3.062615	-0.490031	-1.564107
C	2.035678	-2.868608	0.436373
H	1.734306	-3.579265	-0.340224
H	2.986699	-3.192386	0.8712
H	1.271259	-2.827483	1.210454
C	-1.00686	-0.152695	2.393383
H	-1.796175	-0.435152	1.701008
H	-1.180152	-0.631558	3.362025
H	-1.000292	0.934524	2.519311
C	1.301723	-0.396697	2.75702
H	2.217714	-0.708349	2.257438
H	1.38049	0.65944	3.032433
H	1.146416	-0.997805	3.65829
C	-1.921082	-0.620181	-1.055991
C	-2.983987	-1.372749	-0.541374
C	-4.037245	-0.756411	0.125725
C	-4.036617	0.62544	0.302297
C	-2.977166	1.383356	-0.19294
C	-1.928446	0.769011	-0.867419
H	-2.983291	-2.450491	-0.671868
H	-4.855067	-1.354309	0.511947
H	-4.853155	1.108005	0.827311
H	-2.96394	2.458004	-0.047487
H	-1.098582	1.368877	-1.225206
C	-0.823634	-1.309256	-1.758924
H	-0.809081	-2.392244	-1.653681
C	0.122289	-0.721382	-2.496753
H	0.893434	-1.31335	-2.976069
H	0.157228	0.349431	-2.664923

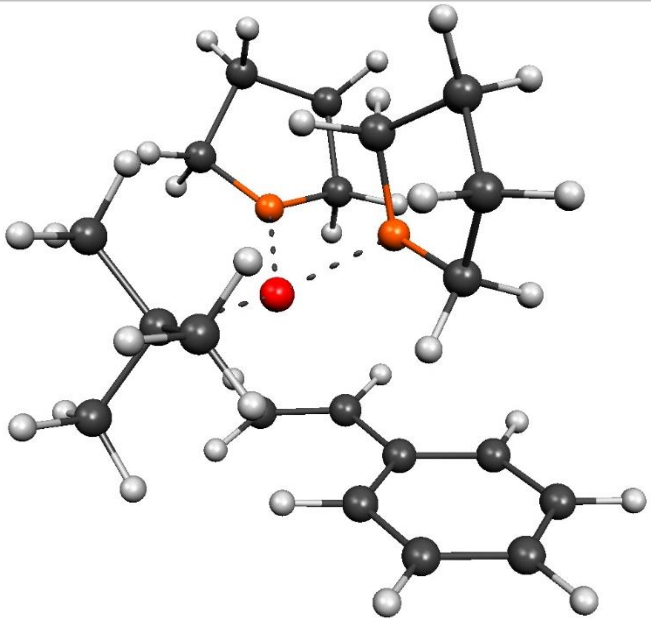

Figure S44: Molekel plot of **25**.

Table S18: Standard orientation of **24** [M062x/6-311+G(d)], T=298 K, E_{SCF} = – 939.727325663 H, E_{ZPE} = – 939.230859 H, no imaginary frequency.

Atomic symbol	x	y	z
C	0.286086	2.372522	0.93673
C	-0.26287	3.67351	0.361523
H	0.338565	4.043783	-0.478681
H	-1.297367	3.588085	0.000889
H	-0.279164	4.488115	1.11618
C	1.673538	2.702885	1.489879
H	1.630105	3.475927	2.287317
H	2.179197	1.835029	1.938788
H	2.340298	3.103935	0.716032
C	-0.585322	1.994639	2.133997
H	-0.621451	2.80072	2.898552
H	-1.626387	1.786837	1.852094
H	-0.21086	1.101425	2.653701
Li	0.483293	0.61566	-0.194422
O	2.184517	-0.004478	-0.920231
O	0.152355	-1.105524	0.720797
C	3.478156	0.585995	-0.840826
H	3.72532	1.049986	-1.804558
H	3.444822	1.351329	-0.069042
C	2.322943	-1.225508	-1.641981
H	2.202905	-1.021396	-2.712409
H	1.517471	-1.884534	-1.315388
C	-1.098624	-1.456918	1.350427
H	-1.530149	-2.292683	0.794164
H	-1.768555	-0.60086	1.283081
C	1.246808	-1.404756	1.601352
H	2.053403	-1.838427	1.007741
H	1.601614	-0.470541	2.050793
C	-2.076528	-0.053645	-1.623088
C	-2.670151	-1.314053	-1.750139
C	-3.83168	-1.630668	-1.055567
C	-4.416658	-0.68941	-0.213223
C	-3.831744	0.567317	-0.073308
C	-2.671295	0.88342	-0.767987
H	-2.213819	-2.051432	-2.404082
H	-4.279718	-2.611578	-1.170501
H	-5.321761	-0.933197	0.331676
H	-4.273532	1.302639	0.590171
H	-2.209375	1.852302	-0.621121
C	-0.839312	0.24037	-2.365675
H	-0.392521	-0.609954	-2.877806
C	-0.239524	1.432275	-2.451836
H	0.685432	1.544438	-3.006601
H	-0.633652	2.328135	-1.985493
C	4.395049	-0.594912	-0.537702
H	5.428879	-0.410691	-0.829823
H	4.377527	-0.802253	0.534073

C	3.742509	-1.748224	-1.329717

Table S19: Standard orientation of **24** [M062x/6-311+G(d)], T=193 K, E_{SCF} = – 939.727325694 H, E_{ZPE} = – 939.230858 H, no imaginary frequency.

Atomic symbol	x	y	z
C	0.286043	2.372708	0.936447
C	-0.263044	3.673617	0.361175
H	0.338301	4.043875	-0.479102
H	-1.297589	3.588204	0.000663
H	-0.27929	4.48826	1.115789
C	1.673325	2.703362	1.489879
H	1.629585	3.476555	2.287143
H	2.178939	1.835631	1.939087
H	2.340266	3.10429	0.716135
C	-0.585511	1.994779	2.13365
H	-0.621832	2.800906	2.898139
H	-1.626516	1.786834	1.851623
H	-0.211061	1.101621	2.653477
Li	0.483532	0.615709	-0.194191
O	2.184712	-0.003998	-0.920426
O	0.152525	-1.105683	0.720594
C	3.478561	0.585972	-0.840338
H	3.726222	1.050235	-1.803805
H	3.445266	1.351051	-0.06829
C	2.322949	-1.224965	-1.64235
H	2.20334	-1.020614	-2.712778
H	1.517145	-1.883779	-1.316148
C	-1.098416	-1.457138	1.350241
H	-1.529973	-2.29286	0.793927
H	-1.768393	-0.601112	1.283008
C	1.246988	-1.404711	1.601239
H	2.053772	-1.838011	1.007638
H	1.601454	-0.470444	2.050871
C	-2.076621	-0.05379	-1.622866
C	-2.670309	-1.31416	-1.749925
C	-3.831849	-1.630721	-1.055335
C	-4.416754	-0.689442	-0.21297
C	-3.831772	0.56726	-0.073054
C	-2.671328	0.883304	-0.767754
H	-2.214022	-2.051559	-2.403878
H	-4.279944	-2.611602	-1.170281
H	-5.321857	-0.933169	0.331956
H	-4.273502	1.302577	0.590469
H	-2.209283	1.852122	-0.620888
C	-0.839391	0.240197	-2.365442
H	-0.39256	-0.61016	-2.877483
C	-0.239671	1.432121	-2.451747
H	0.685288	1.544257	-3.00651
H	-0.633825	2.328024	-1.985509
C	4.39493	-0.595357	-0.537284
H	5.428892	-0.411415	-0.829125
H	4.377144	-0.802932	0.534442
C	3.742222	-1.748252	-1.329748

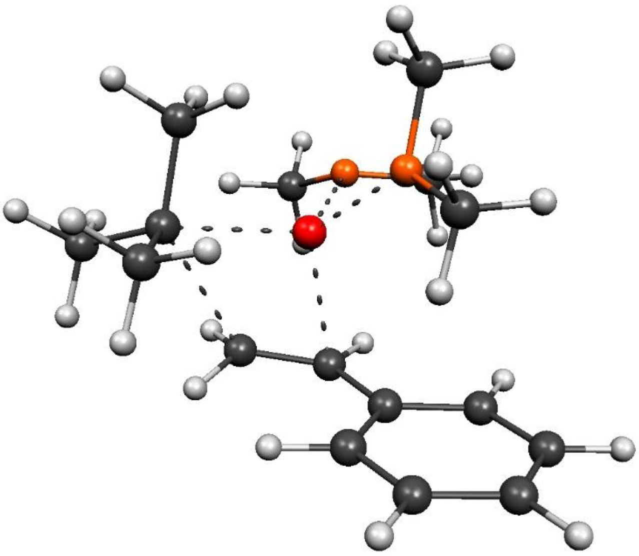

Figure S45: Molekel plot for **TS2-m$_{DME}$**.

Table S20: Standard orientation of **TSm-2$_{DME}$** [M062x/6-311+G(d)], T=298 K, E_{SCF} = – 784.902250803 H, E_{ZPE} = – 784.478355 H, one imaginary frequency.

Atomic symbol	x	y	z
C	-2.024454	-1.552685	0.365028
C	-3.021303	-2.387489	-0.408926
H	-3.700416	-1.771449	-1.011777
H	-2.53458	-3.094165	-1.091011
H	-3.656499	-2.988168	0.264664
C	-2.79653	-0.604763	1.287385
H	-3.487208	-1.173913	1.937053
H	-2.142347	-0.051451	1.970711
H	-3.411555	0.127863	0.753137
C	-1.157873	-2.455299	1.231985
H	-1.764375	-3.013913	1.96838
H	-0.623702	-3.207714	0.640927
H	-0.405847	-1.895507	1.803327
Li	-0.788536	0.166267	0.064664
O	-1.752057	1.720279	-0.677824
O	0.070023	1.154872	1.53225
C	-2.699689	1.413771	-1.68496
H	-3.420029	2.231166	-1.792108
H	-2.195674	1.245777	-2.64355
H	-3.211952	0.501849	-1.38493
C	-0.995891	2.874848	-0.989799
H	-0.535048	2.779727	-1.978866
H	-1.634827	3.764139	-0.975818
H	-0.211116	2.966433	-0.240607
C	1.32691	0.883563	2.138225
H	1.835124	0.133879	1.538222
H	1.932305	1.794772	2.170659
H	1.17715	0.507993	3.155873
C	-0.68076	2.101088	2.270609
H	-1.619179	2.264859	1.743096
H	-0.889538	1.724219	3.277421
H	-0.128983	3.044516	2.349967
C	1.647365	-0.415748	-1.110069
C	2.537393	0.675336	-1.195674
C	3.786232	0.646634	-0.593707
C	4.204066	-0.476963	0.120325
C	3.344501	-1.570176	0.210191
C	2.088858	-1.541422	-0.383276
H	2.229664	1.557208	-1.751388
H	4.442577	1.50642	-0.682268
H	5.180709	-0.501874	0.589317
H	3.652277	-2.455802	0.756665
H	1.439563	-2.403157	-0.279256
C	0.333059	-0.33684	-1.72296
H	0.124544	0.570001	-2.285661
C	-0.664128	-1.282295	-1.589282
H	-1.558185	-1.19232	-2.195606
H	-0.427505	-2.300758	-1.306537

Table S21: Standard orientation of **TSm-2$_{DME}$** [M062x/6-311+G(d)], T=193 K, E_{SCF} = – 784.902250803 H, E_{ZPE} = – 784.478355 H, one imaginary frequency.

Atomic symbol	x	y	z
C	-2.024454	-1.552685	0.365028
C	-3.021303	-2.387489	-0.408926
H	-3.700416	-1.771449	-1.011777
H	-2.53458	-3.094165	-1.091011
H	-3.656499	-2.988168	0.264664
C	-2.79653	-0.604763	1.287385
H	-3.487208	-1.173913	1.937053
H	-2.142347	-0.051451	1.970711
H	-3.411555	0.127863	0.753137
C	-1.157873	-2.455299	1.231985
H	-1.764375	-3.013913	1.96838
H	-0.623701	-3.207714	0.640927
H	-0.405847	-1.895506	1.803327
Li	-0.788536	0.166267	0.064664
O	-1.752057	1.720279	-0.677824
O	0.070023	1.154872	1.53225
C	-2.699689	1.413771	-1.68496
H	-3.420029	2.231166	-1.792108
H	-2.195674	1.245777	-2.64355
H	-3.211952	0.501849	-1.38493
C	-0.995891	2.874848	-0.989799
H	-0.535048	2.779727	-1.978866
H	-1.634827	3.764139	-0.975818
H	-0.211117	2.966433	-0.240607
C	1.32691	0.883563	2.138225
H	1.835124	0.133879	1.538222

H	1.932305	1.794771	2.170659
H	1.17715	0.507992	3.155873
C	-0.68076	2.101088	2.27061
H	-1.619179	2.264859	1.743096
H	-0.889539	1.724218	3.277421
H	-0.128983	3.044516	2.349967
C	1.647365	-0.415748	-1.110069
C	2.537393	0.675337	-1.195674
C	3.786232	0.646634	-0.593707
C	4.204066	-0.476963	0.120325
C	3.344501	-1.570176	0.210191
C	2.088858	-1.541422	-0.383276
H	2.229664	1.557208	-1.751388
H	4.442577	1.50642	-0.682268
H	5.180709	-0.501874	0.589317
H	3.652277	-2.455802	0.756664
H	1.439563	-2.403157	-0.279256
C	0.333059	-0.33684	-1.72296
H	0.124544	0.570001	-2.285661
C	-0.664128	-1.282295	-1.589282
H	-1.558185	-1.19232	-2.195606
H	-0.427505	-2.300758	-1.306537

Table S22: Standard orientation of **TSm-2$_{\mathbf{DME}}$** [M062x/6-311+G(d), scrf=(pcm,water)], T=298 K, E_{SCF} = – 784.912881513 H, E_{ZPE} = – 784.490176 H, one imaginary frequency.

Atomic symbol	x	y	z
C	-1.993952	-1.595024	0.353656
C	-2.927666	-2.476237	-0.445493
H	-3.61737	-1.891937	-1.067158
H	-2.389838	-3.159952	-1.113608
H	-3.55276	-3.10829	0.21239
C	-2.833229	-0.685734	1.252128
H	-3.497132	-1.287526	1.904075
H	-2.225111	-0.078065	1.931693
H	-3.483548	-0.003431	0.694275
C	-1.102364	-2.453204	1.237583
H	-1.696312	-3.051062	1.956903
H	-0.509277	-3.169335	0.656944
H	-0.400559	-1.854046	1.831965
Li	-0.821622	0.177885	0.057145
O	-1.803383	1.684962	-0.697816
O	0.001148	1.132849	1.548763
C	-2.753111	1.388212	-1.708233
H	-3.504663	2.180689	-1.768928
H	-2.25658	1.286317	-2.67953
H	-3.228314	0.445225	-1.444711
C	-1.107351	2.893054	-0.961053
H	-0.647721	2.86301	-1.954348
H	-1.792309	3.744242	-0.907915
H	-0.328221	2.995927	-0.207875
C	1.226778	0.811751	2.192058
H	1.747878	0.085125	1.573026
H	1.841749	1.710468	2.298224
H	1.031823	0.387292	3.181961
C	-0.752764	2.079184	2.291489
H	-1.673827	2.271636	1.743946
H	-0.991202	1.682212	3.283161
H	-0.186806	3.009239	2.402507
C	1.700551	-0.365786	-1.110823
C	2.553322	0.757051	-1.146548
C	3.800711	0.743432	-0.537742
C	4.252354	-0.396715	0.129
C	3.429422	-1.522141	0.166689
C	2.17668	-1.508935	-0.435017
H	2.214165	1.655516	-1.655126
H	4.425354	1.629882	-0.579053
H	5.224892	-0.407697	0.607384
H	3.763121	-2.418593	0.679386
H	1.554828	-2.394876	-0.370151
C	0.383749	-0.299949	-1.727314
H	0.148064	0.627919	-2.243528
C	-0.593552	-1.264618	-1.618079
H	-1.497164	-1.172964	-2.209617
H	-0.347874	-2.27912	-1.329116

Table S23: Standard orientation of **TSm-2$_{\mathbf{DME}}$** [M062x/6-311+G(d), scrf=(pcm,thf)], T=298 K, E_{SCF} = – 784.910580246 H, E_{ZPE} = –784.487614 H, one imaginary frequency.

Atomic symbol	x	y	z

C	-2.001922	-1.585436	0.357669
C	-2.954927	-2.450117	-0.437197
H	-3.639806	-1.854026	-1.053011
H	-2.431999	-3.140258	-1.110245
H	-3.584962	-3.073451	0.223351
C	-2.820858	-0.665119	1.264556
H	-3.493077	-1.25792	1.91531
H	-2.198352	-0.073859	1.945598
H	-3.460631	0.033125	0.714207
C	-1.119909	-2.460687	1.234852
H	-1.720185	-3.051197	1.954123
H	-0.541485	-3.184621	0.649237
H	-0.405616	-1.875664	1.828307
Li	-0.812099	0.174158	0.060924
O	-1.7887	1.692652	-0.693056
O	0.02249	1.141493	1.543561
C	-2.742969	1.396876	-1.69897
H	-3.486189	2.196909	-1.766067
H	-2.249145	1.279915	-2.670038
H	-3.227657	0.46155	-1.425888
C	-1.078226	2.888342	-0.969297
H	-0.622166	2.844084	-1.963883
H	-1.75146	3.749446	-0.921495
H	-0.294715	2.986907	-0.220054
C	1.256054	0.8365	2.179746
H	1.778022	0.107471	1.564817
H	1.865024	1.741004	2.270743
H	1.072107	0.421222	3.175713
C	-0.733275	2.08727	2.283802
H	-1.658888	2.269767	1.740453
H	-0.963817	1.69599	3.279726
H	-0.172799	3.022056	2.385833
C	1.686449	-0.379383	-1.110929
C	2.551031	0.733872	-1.162231
C	3.799041	0.715389	-0.555693
C	4.240198	-0.420253	0.125413
C	3.405614	-1.53603	0.179422
C	2.151859	-1.517747	-0.419618
H	2.221439	1.627954	-1.684634
H	4.433526	1.594098	-0.611256
H	5.21401	-0.435497	0.601037
H	3.730993	-2.429332	0.702844
H	1.521097	-2.396208	-0.342129
C	0.370164	-0.309327	-1.725662
H	0.140494	0.614862	-2.250855
C	-0.612349	-1.268995	-1.610674
H	-1.514311	-1.175749	-2.204495
H	-0.369329	-2.285152	-1.325198

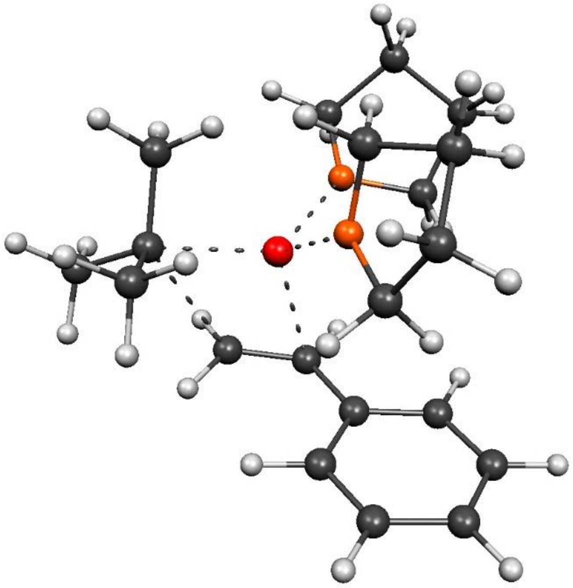

Figure S46: Molekel plot for **TS2-m$_{THF}$.**

Table S24: Standard orientation of **TSm-2$_{THF}$** [M062x/6-311+G(d)], T=298 K, E_{SCF} = – 939.723779864 H, E_{ZPE} = – 939.226487 H, one imaginary frequency.

Atomic symbol	x	y	z

C	1.241345	2.649464	0.51205
C	1.524609	4.029291	-0.038608
H	2.196684	3.9995	-0.904675
H	0.613148	4.550736	-0.355335
H	2.00615	4.680242	0.714417
C	2.565969	2.018421	0.931617
H	3.035011	2.568945	1.771952
H	2.476135	0.976201	1.272962
H	3.292492	2.032034	0.111575
C	0.328399	2.767175	1.723753
H	0.760872	3.428873	2.500631
H	-0.648026	3.194114	1.462276
H	0.14307	1.800752	2.208281
Li	0.672624	0.751302	-0.19613
O	1.841711	-0.573744	-1.045692
O	0.075204	-0.517258	1.182698
C	3.213828	-0.694571	-1.404449
H	3.316371	-0.544194	-2.486053
H	3.766592	0.081495	-0.879717
C	1.218678	-1.798574	-1.4311
H	0.982977	-1.753256	-2.500969
H	0.289308	-1.88434	-0.867264
C	-1.249523	-0.744244	1.712806
H	-1.859903	-1.157975	0.908341
H	-1.673122	0.214509	2.014261
C	1.0361	-1.216228	1.978248
H	1.868293	-1.488945	1.327836
H	1.407176	-0.554111	2.769997
C	-1.999378	0.481561	-1.379455
C	-2.418824	-0.798016	-1.790801
C	-3.579198	-1.377491	-1.296067
C	-4.368997	-0.695292	-0.371344
C	-3.977907	0.578528	0.037559
C	-2.813912	1.155956	-0.450363
H	-1.819398	-1.335342	-2.520859
H	-3.873667	-2.364674	-1.637034
H	-5.277745	-1.143173	0.013836
H	-4.585371	1.126613	0.750531
H	-2.528244	2.142079	-0.100697
C	-0.764914	1.047669	-1.910403
H	-0.238814	0.436566	-2.640111
C	-0.228984	2.255288	-1.56058
H	0.620437	2.652131	-2.101454
H	-0.789437	2.985214	-0.990455
C	3.588686	-2.12267	-1.000214
H	4.381195	-2.530433	-1.627654
H	3.942051	-2.137975	0.032211

Table S25: Standard orientation of **TSm-2$_{THF}$** [M062x/6-311+G(d)], T=193 K, E_{SCF} = – 939.723780386 H, E_{ZPE} = – 939.226489 H, one imaginary frequency.

Atomic symbol	x	y	z
C	1.242829	2.648992	0.511271
C	1.525915	4.028678	-0.039829
H	2.197566	3.998673	-0.90622
H	0.614287	4.550028	-0.356239
H	2.007832	4.679817	0.712802
C	2.567584	2.017911	0.930378
H	3.037105	2.568544	1.770383
H	2.47776	0.975763	1.271944
H	3.29373	2.031243	0.109989
C	0.330471	2.767074	1.723393
H	0.763427	3.428797	2.499993
H	-0.645998	3.194174	1.462327
H	0.145159	1.800767	2.208163
Li	0.672931	0.751139	-0.196652
O	1.84121	-0.574673	-1.046153
O	0.075294	-0.516847	1.18243
C	3.213223	-0.696237	-1.40505
H	3.315676	-0.546396	-2.486735
H	3.766409	0.079822	-0.880741
C	1.217601	-1.799396	-1.431006
H	0.982071	-1.754568	-2.500939
H	0.288131	-1.884494	-0.867239
C	-1.249358	-0.74291	1.713103
H	-1.860151	-1.156957	0.909117
H	-1.672475	0.216224	2.014056
C	1.036191	-1.215198	1.978527
H	1.868296	-1.48858	1.328281
H	1.40743	-0.552352	2.769592
C	-1.99977	0.48163	-1.379365
C	-2.419394	-0.798027	-1.790236
C	-3.579775	-1.377216	-1.295171

C	-4.369384	-0.694609	-0.370597
C	-3.978098	0.5793	0.037845
C	-2.8141	1.156434	-0.450412
H	-1.820115	-1.335658	-2.520188
H	-3.874389	-2.364482	-1.63577
H	-5.278147	-1.142246	0.014831
H	-4.585448	1.127711	0.750663
H	-2.528232	2.142618	-0.101074
C	-0.765294	1.047394	-1.910711
H	-0.239506	0.435992	-2.6404
C	-0.229	2.254896	-1.561275
H	0.620569	2.651374	-2.102176
H	-0.788891	2.984961	-0.990773
C	3.587464	-2.124328	-1.000197
H	4.379633	-2.532777	-1.627612
H	3.941025	-2.139254	0.032161
C	2.254338	-2.898549	-1.135922

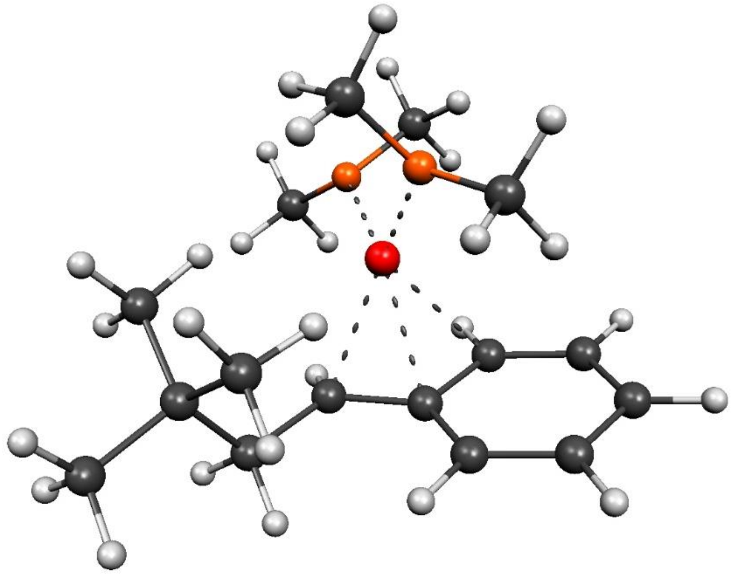

Figure S47: Molekel plot of **10**.

Table S26: Standard orientation of **10** [M062x/6-311+G(d)], T=298 K, E_{SCF} = – 784.963635850 H, E_{ZPE} = – 784.537348 H, no imaginary frequency.

Atomic symbol	x	y	z
C	-2.877862	-0.317302	-0.056683
C	-4.329084	-0.789361	-0.175497
H	-4.809301	-0.363062	-1.061497
H	-4.379109	-1.878925	-0.262224
H	-4.918336	-0.495537	0.698802
C	-2.85552	1.211023	0.015374
H	-3.463062	1.57549	0.85004
H	-1.834807	1.582518	0.151965
H	-3.250166	1.653151	-0.905754
C	-2.266021	-0.891837	1.222712
H	-2.363799	-1.981098	1.25185
H	-1.199164	-0.655228	1.281966
H	-2.762726	-0.489451	2.112504
Li	0.586486	0.715102	-0.065322
O	0.922767	2.54045	-0.531913
O	0.941909	0.75971	1.793538
C	0.34282	3.121537	-1.687393
H	0.367108	4.213059	-1.612141
H	0.881777	2.805745	-2.586985
H	-0.687471	2.775486	-1.741608
C	2.263666	2.950389	-0.323505
H	2.889751	2.661535	-1.174255
H	2.312262	4.035271	-0.187218
H	2.616703	2.448111	0.575778
C	1.444491	-0.417902	2.41513
H	2.04367	-0.948396	1.676208
H	2.060289	-0.144823	3.278019
H	0.61838	-1.058705	2.738639
C	0.151244	1.55317	2.658605
H	-0.163133	2.43172	2.095727
H	-0.73049	0.997151	2.992858
H	0.738189	1.864307	3.528774

C	0.459906	-1.165426	-1.089049
C	1.801084	-0.707767	-1.348851
C	2.926068	-1.411352	-0.931374
C	2.814315	-2.602615	-0.220816
C	1.520788	-3.079869	0.046516
C	0.390356	-2.394169	-0.345633
H	1.932849	0.173523	-1.978316
H	3.909374	-1.024212	-1.184619
H	3.691643	-3.153519	0.094554
H	1.400516	-4.017731	0.582005
H	-0.582599	-2.811917	-0.113592
C	-0.652093	-0.370761	-1.430148
H	-0.457108	0.378512	-2.199166
C	-2.088208	-0.81149	-1.299158
H	-2.64522	-0.468883	-2.181098
H	-2.168569	-1.90829	-1.319637

Table S27: Standard orientation of **10** [M062x/6-311+G(d)], T=193 K, E_{SCF} = – 784.963636253 H, E_{ZPE} = – 784.537341 H, no imaginary frequency.

Atomic symbol	x	y	z
C	-2.87715	-0.319727	-0.056752
C	-4.32808	-0.792689	-0.175523
H	-4.808606	-0.366632	-1.061472
H	-4.377418	-1.882278	-0.262321
H	-4.917474	-0.499296	0.698824
C	-2.855771	1.208614	0.015364
H	-3.463529	1.572665	0.850054
H	-1.835298	1.580768	0.151961
H	-3.25072	1.650525	-0.905741
C	-2.264879	-0.893952	1.222579
H	-2.36197	-1.983278	1.251633
H	-1.19817	-0.656676	1.281816
H	-2.761799	-0.491945	2.112425
Li	0.58602	0.715195	-0.064956
O	0.919593	2.541157	-0.531348
O	0.940734	0.760366	1.79386
C	0.338645	3.121186	-1.686856
H	0.363005	4.212777	-1.612613
H	0.876822	2.804572	-2.586631
H	-0.691694	2.775104	-1.739884
C	2.26054	2.95167	-0.32427
H	2.886065	2.662434	-1.175303
H	2.308903	4.036661	-0.188778
H	2.614434	2.450139	0.575095
C	1.444665	-0.416547	2.415681
H	2.044477	-0.946479	1.676864
H	2.060111	-0.142611	3.278548
H	0.619288	-1.058257	2.739267
C	0.148773	1.552879	2.658622
H	-0.166582	2.430954	2.095554
H	-0.732319	0.995729	2.992675
H	0.735064	1.864867	3.528925
C	0.461302	-1.16489	-1.089288
C	1.802012	-0.705653	-1.348812
C	2.927722	-1.408064	-0.931296
C	2.81723	-2.599568	-0.220948
C	1.524199	-3.078288	0.046192
C	0.393056	-2.393802	-0.345985
H	1.932882	0.175939	-1.978057
H	3.910629	-1.019775	-1.18433
H	3.695142	-3.149536	0.094426
H	1.404909	-4.016344	0.581561
H	-0.579467	-2.812635	-0.114077
C	-0.651511	-0.371439	-1.430403
H	-0.457281	0.378369	-2.199077
C	-2.087222	-0.813386	-1.299268
H	-2.644602	-0.471332	-2.181189
H	-2.166658	-1.910252	-1.319614

Table S28: Standard orientation of **10** [M062x/6-311+G(d),scrf=(pcm,water)], T=298 K, E_{SCF} = – 784.977946762 H, E_{ZPE} = –784.552403 H, no imaginary frequency.

Atomic symbol	x	y	z
C	-1.833336	-2.035652	0.109017
C	-2.647717	-3.332044	0.131138
H	-3.270991	-3.420193	-0.764373
H	-1.99058	-4.206682	0.169303
H	-3.30743	-3.370551	1.00356
C	-2.790497	-0.846377	0.01947
H	-3.5017	-0.854208	0.851749

H	-2.244272	0.099873	0.053093
H	-3.362461	-0.869691	-0.914443
C	-1.02038	-1.930841	1.400919
H	-0.40583	-2.823769	1.552505
H	-0.354286	-1.063966	1.369095
H	-1.679867	-1.829102	2.269354
Li	0.084457	1.076509	-0.365337
O	-1.284661	2.37908	-0.774801
O	0.254953	1.370755	1.506432
C	-2.317289	2.228861	-1.736519
H	-3.294034	2.388714	-1.270052
H	-2.181975	2.943349	-2.553412
H	-2.26056	1.212937	-2.121807
C	-1.243939	3.685841	-0.221567
H	-1.05743	4.424983	-1.00566
H	-2.190544	3.916641	0.276345
H	-0.43328	3.708088	0.504619
C	1.447475	1.024861	2.197626
H	2.233966	0.897808	1.454677
H	1.714787	1.819148	2.900985
H	1.30922	0.085754	2.742841
C	-0.847168	1.564538	2.377794
H	-1.717991	1.785821	1.76234
H	-1.034631	0.658639	2.961894
H	-0.65033	2.401356	3.054587
C	1.307806	-0.720527	-1.040295
C	2.109771	0.391954	-1.489953
C	3.41564	0.592852	-1.065147
C	4.041254	-0.287104	-0.178617
C	3.299225	-1.395282	0.260159
C	1.993163	-1.608794	-0.135532
H	1.687203	1.074796	-2.226978
H	3.961098	1.452083	-1.446715
H	5.060957	-0.126278	0.150079
H	3.758713	-2.107936	0.940732
H	1.467833	-2.476365	0.246577
C	-0.040998	-0.850897	-1.410358
H	-0.35879	-0.22892	-2.249372
C	-0.892082	-2.064868	-1.12561
H	-1.5333	-2.255041	-1.997083
H	-0.264772	-2.964622	-1.026642

Table S29: Standard orientation of **10** [M062x/6-311+G(d),scrf=(pcm,thf)], T=298 K, E_{SCF} = – 784.974328781 H, E_{ZPE} = – 784.548541 H, no imaginary frequency.

Atomic symbol	x	y	z
C	-1.978285	-1.918014	0.103499
C	-2.871161	-3.161674	0.122429
H	-3.501105	-3.207115	-0.771506
H	-2.269224	-4.075227	0.1541
H	-3.529665	-3.164098	0.996697
C	-2.86206	-0.672379	0.020718
H	-3.572524	-0.641656	0.853194
H	-2.259966	0.239398	0.057839
H	-3.434956	-0.65675	-0.91282
C	-1.158534	-1.868825	1.39453
H	-0.603031	-2.799942	1.543351
H	-0.435951	-1.04834	1.363036
H	-1.80821	-1.726277	2.264767
Li	0.140128	1.043201	-0.350433
O	-1.087474	2.461572	-0.767284
O	0.361475	1.349672	1.516259
C	-2.137675	2.395021	-1.719733
H	-3.092327	2.643283	-1.246194
H	-1.947372	3.088933	-2.543428
H	-2.172741	1.374973	-2.096377
C	-0.929554	3.762341	-0.221699
H	-0.684546	4.478761	-1.010824
H	-1.849492	4.077173	0.280576
H	-0.115287	3.717079	0.499513
C	1.523639	0.895122	2.197781
H	2.300823	0.73327	1.452179
H	1.845858	1.64633	2.925233
H	1.314806	-0.04789	2.712635
C	-0.722143	1.608976	2.392407
H	-1.572525	1.911767	1.782771
H	-0.981493	0.707791	2.955999
H	-0.461292	2.412369	3.088178
C	1.238298	-0.795663	-1.043021
C	2.103203	0.271728	-1.482334
C	3.422384	0.386494	-1.064134
C	3.994298	-0.538083	-0.189369

C	3.18674	-1.601924	0.243754
C	1.868363	-1.729459	-0.145896
H	1.724411	0.976309	-2.222537
H	4.020108	1.210757	-1.444326
H	5.024975	-0.447396	0.131539
H	3.605086	-2.349167	0.913423
H	1.292497	-2.568343	0.22728
C	-0.118996	-0.837048	-1.409112
H	-0.389729	-0.205031	-2.257328
C	-1.041357	-1.999626	-1.13217
H	-1.692551	-2.146327	-2.00446
H	-0.469926	-2.93612	-1.038918

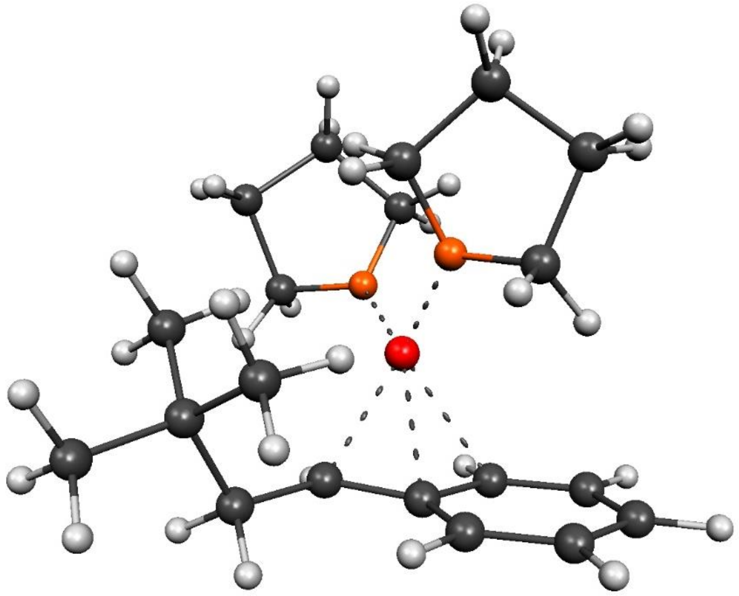

Figure S48: Molekel plot of **25**.

Table S30: Standard orientation of **25** [M062x/6-311+G(d)], T=298 K, E_{SCF} = – 939.786611243 H, E_{ZPE} = – 939.285135 H, no imaginary frequency.

Atomic symbol	x	y	z
C	0.657873	-2.859288	0.780446
C	0.964195	-4.282971	1.252135
H	0.559578	-5.024013	0.555961
H	2.043939	-4.446447	1.320369
H	0.533034	-4.479165	2.23895
C	-0.857266	-2.689727	0.650984
H	-1.361169	-2.924777	1.594789
H	-1.104908	-1.658697	0.379585
H	-1.265624	-3.349783	-0.122623
C	1.177859	-1.862724	1.818472
H	2.243286	-2.016748	2.013306
H	1.047625	-0.836237	1.464576
H	0.648084	-1.976863	2.771115
Li	-0.136419	0.341215	-0.814454
O	-1.938474	0.369988	-1.444438
O	-0.324589	1.207189	0.847448
C	-2.67196	-0.783436	-1.857511
H	-3.025721	-0.635077	-2.88495
H	-1.99329	-1.63415	-1.823747
C	-2.896651	1.386545	-1.139758
H	-3.103205	1.957674	-2.049843
H	-2.440844	2.049872	-0.404374
C	0.694437	2.032369	1.423637
H	1.266788	2.474192	0.608603
H	1.362903	1.399625	2.018282
C	-1.243416	0.924081	1.897148
H	-2.169851	0.568181	1.442708
H	-0.832331	0.124479	2.523346
C	1.904631	-0.197116	-1.269637
C	1.624693	0.955882	-2.08605
C	2.346418	2.140329	-1.981924
C	3.384468	2.280014	-1.065659
C	3.693804	1.169878	-0.263603
C	2.986973	-0.011941	-0.342441
H	0.876244	0.863711	-2.873868
H	2.097906	2.963327	-2.646512

H	3.951334	3.200014	-0.993203
H	4.517398	1.236821	0.442384
H	3.278284	-0.838767	0.294753
C	1.076041	-1.336969	-1.319342
H	0.492091	-1.43245	-2.237293
C	1.353539	-2.628548	-0.590281
H	1.052917	-3.464086	-1.236001
H	2.433443	-2.768258	-0.428272
C	-1.372594	2.24366	2.673904
H	-1.454253	2.063753	3.745902
H	-2.262174	2.794009	2.363382
C	-0.084298	3.019281	2.297736

Table S31: Standard orientation of **25** [M062x/6-311+G(d)], T= 193 K, E_{SCF} = – 939.786611221 H, E_{ZPE} = – 939.285133 H, no imaginary frequency.

Atomic symbol	x	y	z
C	-0.658389	2.859162	0.780424
C	-0.964888	4.282827	1.252054
H	-0.560349	5.023891	0.555858
H	-2.044652	4.446177	1.320267
H	-0.533764	4.479109	2.238867
C	0.856772	2.689769	0.651022
H	1.360603	2.924815	1.594866
H	1.10453	1.658783	0.379573
H	1.265095	3.349922	-0.122521
C	-1.178303	1.862582	1.818472
H	-2.243763	2.016476	2.013226
H	-1.047907	0.83609	1.464655
H	-0.648609	1.97686	2.771144
Li	0.136336	-0.341306	-0.814532
O	1.938453	-0.369615	-1.444142
O	0.324616	-1.206861	0.847571
C	2.671882	0.783803	-1.857361
H	3.025533	0.635357	-2.884828
H	1.993176	1.634483	-1.823587
C	2.896783	-1.386157	-1.139892
H	3.103339	-1.956976	-2.050171
H	2.44111	-2.049749	-0.404667
C	-0.694032	-2.032446	1.423823
H	-1.266546	-2.474092	0.608804
H	-1.362427	-1.400085	2.018962
C	1.243595	-0.923649	1.897143
H	2.169947	-0.567739	1.442546
H	0.832554	-0.123989	2.523297
C	-1.904694	0.196816	-1.269691
C	-1.624582	-0.95615	-2.086099
C	-2.346096	-2.140723	-1.98194
C	-3.384092	-2.280579	-1.06564
C	-3.693604	-1.170477	-0.263591
C	-2.986979	0.011462	-0.342457
H	-0.8762	-0.863835	-2.873966
H	-2.097468	-2.96368	-2.646536
H	-3.950798	-3.200675	-0.993157
H	-4.517171	-1.237552	0.442415
H	-3.278422	0.83825	0.294726
C	-1.076324	1.336789	-1.319421
H	-0.492301	1.43234	-2.237318
C	-1.353993	2.628312	-0.59032
H	-1.053485	3.463898	-1.236032
H	-2.43392	2.767864	-0.428321
C	1.372931	-2.243169	2.674093
H	1.45365	-2.063039	3.746127
H	2.263138	-2.792965	2.364378
C	0.0853	-3.019495	2.297203

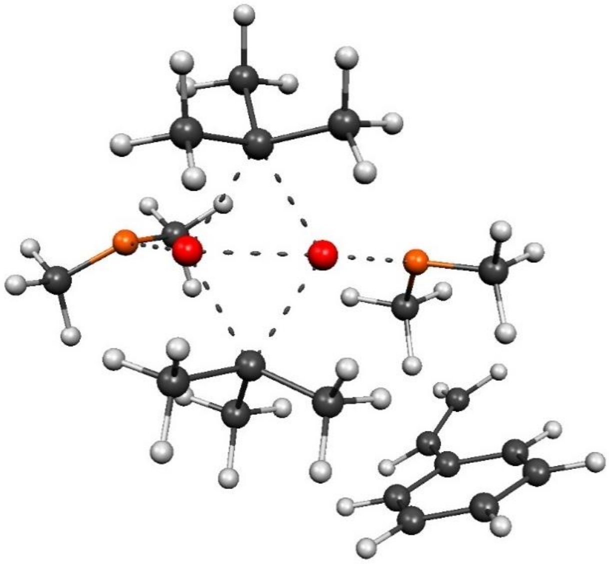

Figure S49: Molekel plot of **1+6.**

Table S32: Standard orientation of **1+6** [M062x/6-311+G(d)], T= 298 K, E_{SCF} = – 950.237783869 H, E_{ZPE} = –949.694158H, no imaginary frequency.

Atomic symbol	x	y	z
C	-1.161544	-0.485852	1.543237
C	-0.298992	0.567681	2.243749
H	-0.8584	1.483446	2.487523
H	0.113935	0.200784	3.206562
H	0.569856	0.865758	1.643338
C	-0.291005	-1.715372	1.300968
H	0.160952	-2.101587	2.239478
H	-0.865578	-2.548777	0.875666
H	0.540122	-1.516716	0.614382
C	-2.246341	-0.896384	2.549473
H	-2.878768	-0.054518	2.86026
H	-2.919692	-1.685059	2.168633
H	-1.817447	-1.320377	3.477606
Li	-2.961702	-0.469691	0.388725
O	-3.247038	-2.042215	-0.662222
C	-3.563254	-3.351955	-0.234597
H	-4.032174	-3.912893	-1.04914
H	-2.663807	-3.879426	0.099718
H	-4.26355	-3.268581	0.595015
C	-2.346145	-2.02509	-1.758314
H	-2.20845	-0.981595	-2.040424
H	-1.386562	-2.465773	-1.467546
H	-2.769102	-2.579617	-2.602032
Li	-1.388637	1.064938	0.032841
C	-3.419758	1.361456	-0.574187
C	-2.949018	2.812292	-0.418778
H	-2.163564	3.070114	-1.142011
H	-3.7649	3.542099	-0.582826
H	-2.557079	3.033081	0.588786
C	-3.921319	1.187353	-2.0049
H	-4.3393	0.186856	-2.176226
H	-4.723666	1.907573	-2.261159
H	-3.122653	1.333343	-2.74304
C	-4.622578	1.185327	0.363156
H	-5.058604	0.171843	0.323392
H	-4.37337	1.38994	1.413143
H	-5.458692	1.863201	0.10648
O	0.184669	1.709006	-0.860468
C	0.831084	2.898469	-0.445656
H	1.453451	3.29147	-1.255981
H	0.056695	3.623196	-0.197563
H	1.458673	2.707371	0.431366
C	1.11169	0.68367	-1.187128
H	1.723472	0.431425	-0.317903
H	0.537375	-0.190117	-1.493873
H	1.759964	1.006043	-2.008463
C	3.143133	-0.535018	1.823874
C	3.47909	0.812482	1.899306
C	4.208985	1.399566	0.866531
C	4.585691	0.649588	-0.23998
C	4.234721	-0.702344	-0.338561

C	3.526495	-1.285176	0.717494
H	2.562789	-0.996764	2.614739
H	3.175046	1.400301	2.75851
H	4.492505	2.444791	0.929204
H	5.171407	1.112736	-1.026676
H	3.248603	-2.333188	0.658171
C	4.564273	-1.517377	-1.522353
H	4.540529	-2.593965	-1.368242
C	4.848016	-1.050637	-2.737167
H	4.85493	0.010638	-2.9637
H	5.075673	-1.723215	-3.555351

Table S33: Standard orientation of **1+6** [M062x/6-311+G(d)], T= 193 K, E_{SCF} = – 950.237783869 H, E_{ZPE} = –949.694158 H, no imaginary frequency.

Atomic symbol	x	y	z
C	1.161544	0.485852	1.543237
C	0.298992	-0.567681	2.243749
H	0.8584	-1.483446	2.487523
H	-0.113935	-0.200784	3.206562
H	-0.569856	-0.865758	1.643338
C	0.291005	1.715372	1.300968
H	-0.160952	2.101587	2.239478
H	0.865578	2.548777	0.875666
H	-0.540122	1.516716	0.614382
C	2.246341	0.896384	2.549473
H	2.878768	0.054518	2.86026
H	2.919692	1.685059	2.168633
H	1.817447	1.320377	3.477606
Li	2.961702	0.469691	0.388725
O	3.247038	2.042215	-0.662222
C	3.563254	3.351955	-0.234597
H	4.032174	3.912893	-1.04914
H	2.663807	3.879426	0.099718
H	4.26355	3.268581	0.595015
C	2.346145	2.02509	-1.758314
H	2.20845	0.981595	-2.040424
H	1.386562	2.465773	-1.467546
H	2.769102	2.579617	-2.602032
Li	1.388637	-1.064938	0.032841
C	3.419758	-1.361456	-0.574187
C	2.949018	-2.812292	-0.418778
H	2.163564	-3.070114	-1.142011
H	3.7649	-3.542099	-0.582826
H	2.557079	-3.033081	0.588786
C	3.921319	-1.187353	-2.0049
H	4.3393	-0.186856	-2.176226
H	4.723666	-1.907573	-2.261159
H	3.122653	-1.333343	-2.74304
C	4.622578	-1.185327	0.363156
H	5.058604	-0.171843	0.323392
H	4.37337	-1.38994	1.413143
H	5.458692	-1.863201	0.10648
O	-0.184669	-1.709006	-0.860468
C	-0.831084	-2.898469	-0.445656
H	-1.453451	-3.29147	-1.255981
H	-0.056695	-3.623196	-0.197563
H	-1.458673	-2.707371	0.431366
C	-1.11169	-0.68367	-1.187128
H	-1.723472	-0.431425	-0.317903
H	-0.537375	0.190117	-1.493873
H	-1.759964	-1.006043	-2.008463
C	-3.143133	0.535018	1.823874
C	-3.47909	-0.812482	1.899306
C	-4.208985	-1.399566	0.866531
C	-4.585691	-0.649588	-0.23998
C	-4.234721	0.702344	-0.338561
C	-3.526495	1.285176	0.717494
H	-2.562789	0.996764	2.614739
H	-3.175046	-1.400301	2.75851
H	-4.492505	-2.444791	0.929204
H	-5.171407	-1.112736	-1.026676
H	-3.248603	2.333188	0.658171
C	-4.564273	1.517377	-1.522353
H	-4.540529	2.593965	-1.368242
C	-4.848016	1.050637	-2.737167
H	-4.85493	-0.010638	-2.9637
H	-5.075673	1.723215	-3.555351

Table S34: Standard orientation of **1+6** [M062x/6-311+G(d),scrf=(pcm,diethyl ether)], T= 298 K, E_{SCF} = – 950.247848417 H, E_{ZPE} = – 949.698686 H, no imaginary frequency.

Atomic symbol	x	y	z
C	-0.955436	-0.251973	1.987519
C	0.038367	0.754451	2.574874
H	-0.416051	1.730465	2.800356
H	0.463334	0.393919	3.535123
H	0.894273	0.943991	1.916026
C	-0.193567	-1.562103	1.793202
H	0.329235	-1.879006	2.721319
H	-0.854136	-2.393567	1.514888
H	0.571861	-1.493225	1.011207
C	-2.008336	-0.506147	3.073436
H	-2.576928	0.397308	3.328439
H	-2.745227	-1.276668	2.794549
H	-1.554159	-0.87	4.01808
Li	-2.598554	-0.528415	0.5835
O	-2.444034	-2.077462	-0.550501
C	-2.999112	-3.372791	-0.400309
H	-3.247438	-3.795274	-1.378338
H	-2.293649	-4.034663	0.112635
H	-3.906064	-3.275939	0.194108
C	-1.255402	-2.083618	-1.323943
H	-0.873539	-1.062729	-1.345253
H	-0.506634	-2.738316	-0.869587
H	-1.469323	-2.416908	-2.344663
Li	-1.447549	1.411001	0.606245
C	-3.322548	1.188797	-0.495969
C	-3.497808	2.677935	-0.190434
H	-2.581334	3.262275	-0.358406
H	-4.266239	3.148289	-0.83849
H	-3.811893	2.861727	0.845125
C	-2.975277	1.065156	-1.97966
H	-2.924626	0.0183	-2.303764
H	-3.730276	1.559096	-2.627882
H	-2.006793	1.524039	-2.220384
C	-4.696595	0.541187	-0.295733
H	-4.705402	-0.529221	-0.548282
H	-5.06	0.636721	0.736123
H	-5.471418	1.004443	-0.941181
O	0.009999	2.124705	-0.434938
C	0.210444	3.360159	-1.096239
H	0.097412	3.236584	-2.178018
H	-0.535869	4.063068	-0.732497
H	1.210538	3.747418	-0.878384
C	1.006251	1.17112	-0.774624
H	1.991344	1.522202	-0.452375
H	0.764606	0.244838	-0.255549
H	1.014756	0.995831	-1.856644
C	4.020112	0.471095	1.489607
C	4.726877	1.290664	0.614249
C	4.790232	0.960609	-0.738908
C	4.134993	-0.168032	-1.215282
C	3.40156	-0.987595	-0.34806
C	3.373408	-0.664394	1.013034
H	3.969639	0.715144	2.544938
H	5.23592	2.173503	0.98412
H	5.357158	1.582482	-1.422803
H	4.205015	-0.423074	-2.267269
H	2.81735	-1.294295	1.700491
C	2.6401	-2.155261	-0.831327
H	2.407592	-2.908434	-0.081149
C	2.196997	-2.327251	-2.076901
H	2.365255	-1.592982	-2.858489
H	1.640753	-3.215087	-2.355031

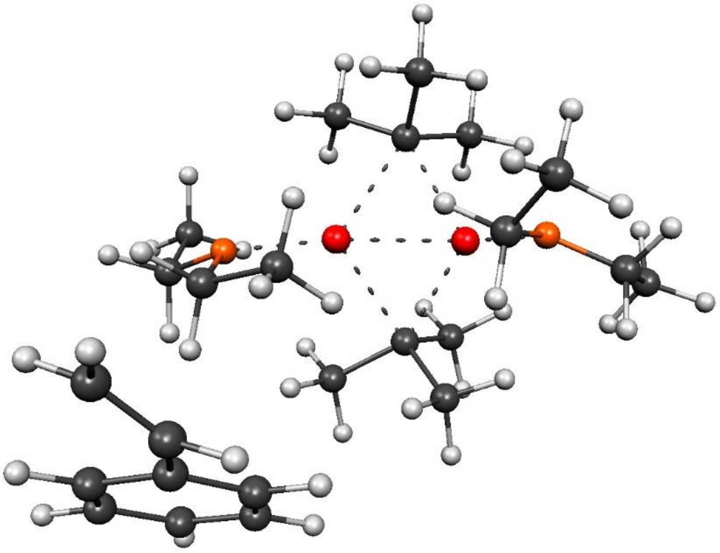

Figure S50: Molekel plot of **1+27.**

Table S35: Standard orientation of **1+26** [M062x/6-311+G(d)], T= 298 K, E_{SCF} = – 1107.46648142 H, E_{ZPE} = –1106.807139 H, no imaginary frequency.

Atomic symbol	x	y	z
C	-0.998343	0.565297	1.798596
C	0.250913	1.374313	2.151179
H	0.127742	2.451746	1.974586
H	0.51881	1.272368	3.2239
H	1.131161	1.0525	1.582896
C	-0.708684	-0.878015	2.220076
H	-0.407805	-0.945025	3.286526
H	-1.574121	-1.544781	2.10884
H	0.107197	-1.324525	1.63655
C	-2.128324	1.092931	2.693427
H	-2.300992	2.165379	2.544294
H	-3.103164	0.604269	2.52614
H	-1.914666	0.949186	3.771174
Li	-2.577583	0.135187	0.428317
O	-3.096538	-1.693322	0.012696
C	-3.766973	-2.676574	0.79159
H	-4.400815	-3.28299	0.137391
H	-3.020826	-3.338604	1.251548
C	-2.085811	-2.217605	-0.850354
H	-1.670924	-1.342493	-1.351342
H	-1.298731	-2.668742	-0.231345
Li	-0.858448	1.469935	-0.217896
C	-2.773947	1.407696	-1.259138
C	-2.303188	2.820656	-1.618155
H	-1.377492	2.819212	-2.208557
H	-3.053127	3.370472	-2.219556
H	-2.122269	3.446432	-0.72995
C	-3.051511	0.669169	-2.56574
H	-3.501087	-0.316407	-2.392072
H	-3.757832	1.221028	-3.217613
H	-2.141365	0.509973	-3.158786
C	-4.120229	1.575878	-0.537177
H	-4.591416	0.613383	-0.278548
H	-4.03322	2.158813	0.390576
H	-4.86741	2.101155	-1.161861
O	0.918279	2.02323	-0.877585
C	1.648735	3.177551	-0.480966
H	2.407307	3.388132	-1.244769
H	2.168837	2.958435	0.460325
C	1.778397	0.920314	-1.165812
H	2.462816	0.770477	-0.324087
H	2.375646	1.165666	-2.05258
C	3.065461	-0.820577	1.957917
C	3.974318	0.232342	2.009626
C	4.921137	0.376578	0.997752
C	4.950795	-0.516421	-0.06668
C	4.029306	-1.566888	-0.141356
C	3.099	-1.713787	0.89356
H	2.320619	-0.934961	2.737747
H	3.945565	0.932909	2.836725
H	5.644878	1.182948	1.04306
H	5.707402	-0.409274	-0.836441

H	2.381963	-2.528886	0.852474
C	3.99777	-2.504981	-1.27875
H	3.525128	-3.464887	-1.080649
C	4.458076	-2.257255	-2.504013
H	4.905248	-1.305096	-2.771199
H	4.392503	-3.001933	-3.288188
C	0.706039	4.350283	-0.329018
H	0.192038	4.561595	-1.267352
H	-0.045758	4.156107	0.438908
H	1.266114	5.238552	-0.030498
C	0.924787	-0.306004	-1.394514
H	0.420817	-0.594769	-0.467114
H	0.172237	-0.109495	-2.164295
H	1.545769	-1.142241	-1.72116
C	-4.600864	-1.983068	1.846354
H	-5.281643	-1.264612	1.384862
H	-5.198742	-2.714369	2.393323
H	-3.968862	-1.458312	2.564579
C	-2.619806	-3.205044	-1.873101
H	-1.837413	-3.428528	-2.601282
H	-2.929601	-4.14933	-1.421834
H	-3.468671	-2.775838	-2.409298

Table S36: Standard orientation of **1+26** [M062x/6-311+G(d)], T= 298 K, E_{SCF} = – 1107.46648145 H, E_{ZPE} = – 1106.807142 H, no imaginary frequency.

Atomic symbol	x	y	z
C	-0.998483	0.5652	1.798557
C	0.250791	1.37421	2.15105
H	0.127516	2.451663	1.974576
H	0.518893	1.272192	3.2237
H	1.130927	1.052575	1.582502
C	-0.708858	-0.878122	2.220029
H	-0.408142	-0.945187	3.286515
H	-1.574229	-1.544959	2.108571
H	0.107164	-1.324462	1.636561
C	-2.128469	1.092879	2.693353
H	-2.301131	2.165317	2.544162
H	-3.103321	0.604223	2.526079
H	-1.91485	0.949177	3.771114
Li	-2.5779	0.135287	0.428237
O	-3.096999	-1.69315	0.01248
C	-3.767438	-2.676414	0.791322
H	-4.401303	-3.282796	0.137104
H	-3.02132	-3.338488	1.251261
C	-2.086208	-2.217356	-0.850523
H	-1.671488	-1.34223	-1.351627
H	-1.29901	-2.668248	-0.231471
Li	-0.858305	1.469887	-0.217763
C	-2.773805	1.408146	-1.259007
C	-2.302876	2.821194	-1.617341
H	-1.377215	2.81988	-2.207797
H	-3.052742	3.371475	-2.218418
H	-2.121791	3.446519	-0.728838
C	-3.05122	0.670131	-2.565914
H	-3.500632	-0.315592	-2.392697
H	-3.757606	1.222128	-3.217609
H	-2.141017	0.511358	-3.158986
C	-4.120063	1.576045	-0.537033
H	-4.591342	0.61344	-0.278963
H	-4.03299	2.158393	0.391095
H	-4.867255	2.101811	-1.161289
O	0.918541	2.022952	-0.877612
C	1.649364	3.177129	-0.48123
H	2.407979	3.387324	-1.245093
H	2.169424	2.958016	0.460088
C	1.778364	0.919839	-1.165979
H	2.463019	0.76995	-0.324431
H	2.375407	1.164977	-2.052941
C	3.06592	-0.820176	1.95824
C	3.975012	0.232519	2.009607
C	4.921626	0.37638	0.997459
C	4.950846	-0.516788	-0.066817
C	4.029091	-1.567076	-0.141146
C	3.098984	-1.713546	0.893978
H	2.32117	-0.934295	2.738193
H	3.946656	0.933227	2.836599
H	5.645548	1.182605	1.042466
H	5.707311	-0.409934	-0.836759
H	2.3815	-2.528254	0.853248
C	3.997107	-2.505366	-1.278361
H	3.523995	-3.464999	-1.080054

C	4.457539	-2.258148	-2.503679
H	4.905192	-1.306283	-2.7711
H	4.391588	-3.002978	-3.287678
C	0.707057	4.350194	-0.329438
H	0.192991	4.56145	-1.267752
H	-0.044696	4.156431	0.438631
H	1.267462	5.23835	-0.031205
C	0.924431	-0.306323	-1.394358
H	0.420521	-0.594865	-0.466854
H	0.171817	-0.109723	-2.164057
H	1.545162	-1.142756	-1.720989
C	-4.601298	-1.982901	1.846102
H	-5.282096	-1.264455	1.384632
H	-5.199132	-2.714211	2.39312
H	-3.969272	-1.458159	2.564319
C	-2.620073	-3.204975	-1.873161
H	-1.837688	-3.428363	-2.601382
H	-2.929702	-4.149286	-1.42182
H	-3.469039	-2.775938	-2.409333

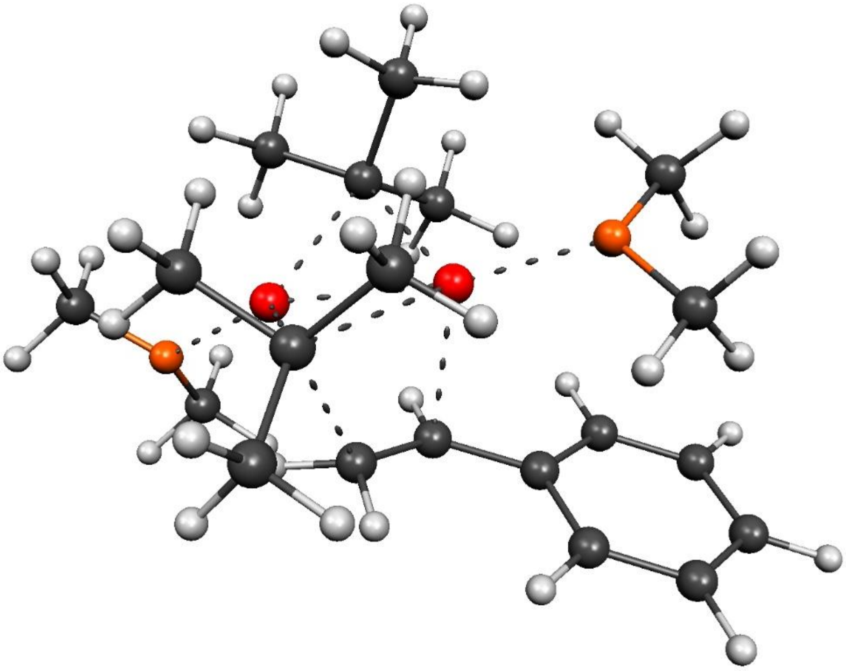

Figure S51: Molekel plot of **TSd$_{DME}$**.

Table S37: Standard orientation of **TSd$_{DME}$** [M062x/6-311+G(d)], T= 298 K, E_{SCF} = – 950.223605237 H, E_{ZPE} = – 949.676172 H, one imaginary frequency.

Atomic symbol	x	y	z
C	-1.460415	1.860443	-0.284861
C	-2.883399	2.080529	-0.821354
H	-3.667413	1.802629	-0.101115
H	-3.065621	3.143912	-1.066943
H	-3.074738	1.519609	-1.744461
C	-0.523255	2.267428	-1.430143
H	-0.595807	3.346044	-1.671169
H	0.538783	2.063524	-1.241653
H	-0.76542	1.723696	-2.351955
C	-1.307378	2.866296	0.865553
H	-2.067613	2.707543	1.640417
H	-0.340927	2.82742	1.382104
H	-1.429469	3.910043	0.515933
Li	0.132952	0.604606	0.540131
O	1.801906	1.585971	1.073998
C	2.256539	2.820542	0.539703
H	2.821208	3.368224	1.301029
H	2.897676	2.636469	-0.32916
H	1.391853	3.407612	0.241558
C	2.893991	0.856268	1.618846
H	2.52884	-0.11384	1.944607
H	3.665716	0.702836	0.859767
H	3.309479	1.399599	2.473899
Li	-2.032765	-0.172775	-0.039266
C	-1.26927	-1.071253	1.937806

C	-2.739815	-0.653296	1.991562
H	-3.375667	-1.318497	1.391304
H	-3.131469	-0.715066	3.020118
H	-2.915067	0.387829	1.681262
C	-1.180673	-2.484085	2.481847
H	-0.143687	-2.811847	2.611598
H	-1.662036	-2.562809	3.469649
H	-1.675226	-3.212055	1.827119
C	-0.447145	-0.146699	2.823094
H	0.620134	-0.392501	2.79316
H	-0.564493	0.915402	2.578714
H	-0.746401	-0.239043	3.881986
O	-3.311882	-1.026312	-1.247795
C	-4.700567	-0.749009	-1.177221
H	-5.273037	-1.626033	-1.49373
H	-4.939246	-0.510877	-0.141868
H	-4.955036	0.105912	-1.810617
C	-2.887941	-1.297705	-2.575544
H	-3.014896	-0.410203	-3.204272
H	-1.83524	-1.575612	-2.539947
H	-3.467344	-2.126453	-2.993242
C	4.070616	-1.859354	-0.224944
C	4.736474	-0.955404	-1.05087
C	3.978014	-0.07891	-1.826798
C	2.591651	-0.105266	-1.773586
C	1.895614	-1.011398	-0.944226
C	2.684082	-1.885507	-0.16576
H	4.63991	-2.558597	0.379274
H	5.819099	-0.940835	-1.09652
H	4.472507	0.628995	-2.484559
H	2.018808	0.576811	-2.395961
H	2.20619	-2.612511	0.482779
C	0.440602	-1.040906	-0.961084
H	-0.025828	-0.460896	-1.753632
C	-0.348953	-1.771262	-0.077876
H	0.134011	-2.513219	0.541629
H	-1.345931	-2.094688	-0.374195

Table S38: Standard orientation of **TSd$_{DME}$** [M062x/6-311+G(d)], T= 193 K, E_{SCF} = – 950.223605981 H, E_{ZPE} = – 949.676178 H, one imaginary frequency.

Atomic symbol	x	y	z
C	-1.460485	1.860742	-0.282222
C	-2.883618	2.081795	-0.817926
H	-3.667414	1.802806	-0.097869
H	-3.065841	3.145587	-1.061759
H	-3.075333	1.522379	-1.741859
C	-0.523678	2.269149	-1.427281
H	-0.59592	3.348168	-1.666598
H	0.538391	2.064519	-1.23964
H	-0.766513	1.726944	-2.349817
C	-1.306987	2.864933	0.869583
H	-2.06716	2.705259	1.644323
H	-0.340489	2.82513	1.385963
H	-1.42891	3.909202	0.521455
Li	0.133257	0.60392	0.54037
O	1.80205	1.585227	1.075228
C	2.25613	2.820523	0.542184
H	2.820623	3.367688	1.304015
H	2.89727	2.637626	-0.326927
H	1.391105	3.407466	0.244733
C	2.894457	0.855482	1.619356
H	2.529775	-0.115035	1.944385
H	3.666122	0.702993	0.860039
H	3.309846	1.398299	2.474782
Li	-2.032957	-0.172743	-0.039144
C	-1.269063	-1.074478	1.935933
C	-2.739524	-0.656343	1.991064
H	-3.375725	-1.320672	1.390239
H	-3.130567	-0.719408	3.019758
H	-2.914781	0.385211	1.682175
C	-1.180496	-2.48814	2.477838
H	-0.143507	-2.816214	2.606645
H	-1.66146	-2.568275	3.465718
H	-1.675401	-3.21505	1.822203
C	-0.446368	-0.151454	2.822276
H	0.620891	-0.397193	2.791181
H	-0.563891	0.911062	2.579788
H	-0.744896	-0.245668	3.881197
O	-3.312436	-1.024249	-1.248618
C	-4.701132	-0.747345	-1.17712
H	-5.273588	-1.623807	-1.495219

H	-4.939583	-0.51126	-0.141242
H	-4.955797	0.10879	-1.808791
C	-2.888867	-1.293139	-2.576983
H	-3.01656	-0.40471	-3.204254
H	-1.835977	-1.570438	-2.542151
H	-3.467875	-2.121585	-2.995829
C	4.07061	-1.859101	-0.226476
C	4.736678	-0.954473	-1.051473
C	3.978402	-0.077176	-1.826685
C	2.592031	-0.103492	-1.773756
C	1.895739	-1.010354	-0.945387
C	2.684059	-1.885165	-0.167498
H	4.639764	-2.558902	0.377233
H	5.819313	-0.939964	-1.096928
H	4.473058	0.631319	-2.483684
H	2.01934	0.579234	-2.395572
H	2.206089	-2.612643	0.480427
C	0.440703	-1.039624	-0.96276
H	-0.025374	-0.458436	-1.75465
C	-0.349266	-1.771238	-0.080878
H	0.133475	-2.514478	0.537245
H	-1.346255	-2.093918	-0.377991

Table S39: Standard orientation of **TSd$_{DME}$** [M062x/6-311+G(d),scrf=(pcm,diethyl ether)], T= 298 K, E_{SCF} = – 950.230024337 H, E_{ZPE} = – 949.683071 H, one imaginary frequency.

Atomic symbol	x	y	z
C	-1.481396	1.87143	-0.100379
C	-2.922476	2.160402	-0.546865
H	-3.677932	1.831063	0.181784
H	-3.092324	3.246297	-0.686415
H	-3.169575	1.688057	-1.504611
C	-0.589548	2.362077	-1.24807
H	-0.646792	3.460812	-1.38673
H	0.475845	2.121566	-1.135197
H	-0.892642	1.907381	-2.199438
C	-1.261764	2.762064	1.131765
H	-2.005645	2.552646	1.910259
H	-0.283891	2.652474	1.614076
H	-1.364579	3.837526	0.881771
Li	0.134285	0.546077	0.554144
O	1.808141	1.549494	1.127222
C	2.226769	2.818892	0.64342
H	2.788002	3.345456	1.421186
H	2.860877	2.690569	-0.240317
H	1.345062	3.398311	0.383119
C	2.922057	0.839181	1.654732
H	2.582161	-0.142044	1.97184
H	3.691778	0.717917	0.887919
H	3.331691	1.380857	2.512985
Li	-2.096954	-0.1749	-0.004391
C	-1.253783	-1.283125	1.796264
C	-2.661904	-0.743082	2.053265
H	-3.418075	-1.261765	1.447668
H	-2.963682	-0.904351	3.101928
H	-2.756452	0.338548	1.884104
C	-1.274937	-2.76663	2.111984
H	-0.270564	-3.202951	2.12822
H	-1.715017	-2.958441	3.104495
H	-1.867071	-3.335023	1.383795
C	-0.266535	-0.590374	2.720196
H	0.758256	-0.935374	2.54167
H	-0.282768	0.505742	2.645149
H	-0.482359	-0.808917	3.781585
O	-3.394775	-0.887305	-1.275023
C	-4.792834	-0.681172	-1.135656
H	-5.339836	-1.524271	-1.56624
H	-5.014436	-0.606016	-0.072562
H	-5.093805	0.245059	-1.633896
C	-3.003091	-0.950926	-2.640157
H	-3.236069	-0.009479	-3.147307
H	-1.928585	-1.12672	-2.673422
H	-3.520888	-1.772776	-3.142164
C	4.070546	-1.83825	-0.280775
C	4.774031	-0.909933	-1.047492
C	4.052517	0.01653	-1.801217
C	2.664437	0.00816	-1.79124
C	1.931278	-0.929056	-1.031315
C	2.681986	-1.845477	-0.263799
H	4.611356	-2.567111	0.314611
H	5.85784	-0.907997	-1.05722

H	4.576982	0.751192	-2.404052
H	2.120408	0.731392	-2.393047
H	2.174098	-2.583242	0.349122
C	0.477102	-0.939205	-1.104667
H	0.040764	-0.265892	-1.838401
C	-0.350475	-1.751303	-0.343774
H	0.089496	-2.573187	0.202365
H	-1.352422	-1.98957	-0.692792

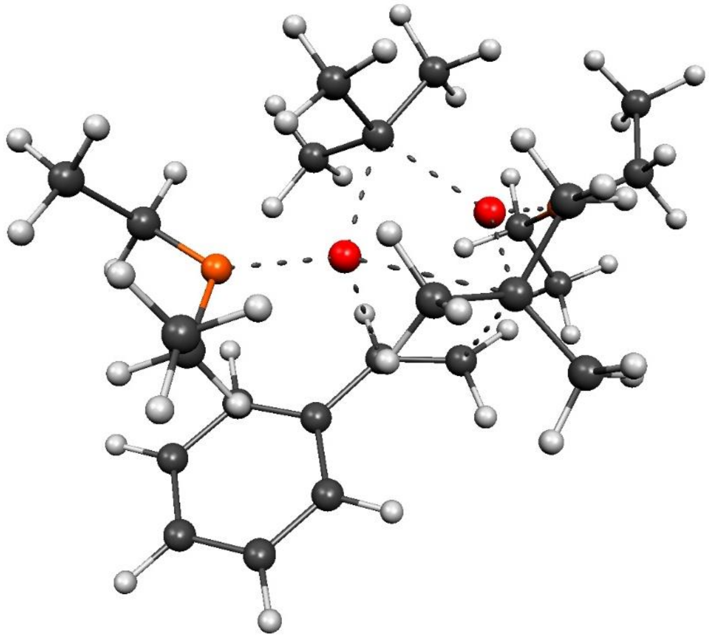

Figure S52: Molekel plot of **TSd**$_{\mathbf{DEE}}$.

Table S40: Standard orientation of **TSd**$_{\mathbf{DEE}}$ [M062x/6-311+G(d)], T= 298 K, E_{SCF} = – 1107.44316823 H, E_{ZPE} = – 1106.781794 H, one imaginary frequency.

Atomic symbol	x	y	z
C	0.900572	-0.354221	2.271982
C	-0.473778	0.246807	2.490306
H	-1.269903	-0.392004	2.081641
H	-0.710725	0.378172	3.561762
H	-0.574506	1.252479	2.05598
C	1.973905	0.673901	2.637201
H	1.874694	1.00644	3.685358
H	2.985821	0.256297	2.555267
H	1.928669	1.594837	2.037263
C	1.090183	-1.558305	3.173366
H	0.244497	-2.25268	3.124319
H	1.993282	-2.127326	2.917534
H	1.184468	-1.263112	4.232156
Li	1.951021	0.296549	0.444328
O	3.649392	-0.422877	-0.191631
C	3.570091	-0.900302	-1.536426
H	4.09415	-0.208078	-2.202217
C	4.960747	-0.330723	0.363638
H	4.816231	0.114297	1.348278
H	5.361132	-1.338727	0.513671
Li	-0.485737	0.609749	0.073908
C	1.074754	1.915948	-0.668872
C	0.499435	1.709987	-2.076918
H	-0.49403	1.238414	-2.104517
H	0.402386	2.659967	-2.638402
H	1.145098	1.056041	-2.675778
C	0.335896	3.084527	0.0077
H	0.828801	3.377123	0.942429
H	0.326531	3.985884	-0.636002
H	-0.710492	2.888577	0.27038
C	2.508443	2.427253	-0.875293
H	3.05514	2.570196	0.06889
H	3.11036	1.755684	-1.495898
H	2.517576	3.410604	-1.382718
O	-2.418112	1.293794	-0.034453
C	-2.878378	2.120999	-1.104613
H	-3.382951	1.482428	-1.840619

C	-3.471705	0.617989	0.66863
H	-4.349194	0.555952	0.017571
C	-3.740761	-1.774887	-1.892716
C	-4.330629	-2.565124	-0.907283
C	-3.540237	-3.018059	0.14795
C	-2.196475	-2.67873	0.229298
C	-1.582035	-1.873284	-0.752015
C	-2.396825	-1.43862	-1.817159
H	-4.333955	-1.419965	-2.729839
H	-5.379438	-2.831669	-0.96599
H	-3.976563	-3.644312	0.919395
H	-1.61517	-3.045894	1.068534
H	-1.95301	-0.824147	-2.5966
C	-0.174963	-1.496367	-0.717385
H	0.202742	-1.012738	-1.615977
C	0.692353	-1.754212	0.326534
H	0.396279	-2.4461	1.101223
H	1.763974	-1.760913	0.156496
C	5.912323	0.520645	-0.459556
H	6.829537	0.686214	0.109032
H	6.193218	0.041659	-1.399525
H	5.466306	1.492133	-0.681258
C	-3.780206	3.258809	-0.657333
H	-4.735136	2.904947	-0.26509
H	-3.286241	3.860279	0.108816
H	-3.99337	3.903842	-1.51268
C	-3.800817	1.289315	1.990887
H	-2.915449	1.342832	2.625907
H	-4.184276	2.301018	1.857255
H	-4.558619	0.703719	2.517677
H	-3.135812	-0.40517	0.83255
H	-1.983917	2.531819	-1.569572
H	2.50879	-0.847525	-1.791988
C	4.086695	-2.31859	-1.694562
H	3.593575	-2.990471	-0.988896
H	3.875869	-2.672876	-2.705217
H	5.166026	-2.382908	-1.540822

Table S41: Standard orientation of **TSd$_{\mathbf{DEE}}$** [M062x/6-311+G(d)], T= 193 K, E_{SCF} = – 1107.44316814 H, E_{ZPE} = – 1106.781797 H, one imaginary frequency.

Atomic symbol	x	y	z
C	0.90129	-0.350682	2.27213
C	-0.473947	0.248484	2.489925
H	-1.269298	-0.391044	2.080852
H	-0.711456	0.379536	3.561264
H	-0.575703	1.254093	2.05567
C	1.973031	0.679905	2.635275
H	1.872712	1.014885	3.682537
H	2.985577	0.263489	2.555051
H	1.926937	1.599225	2.033021
C	1.093626	-1.552945	3.175375
H	0.249499	-2.249279	3.127748
H	1.997892	-2.120376	2.920129
H	1.187638	-1.255833	4.233645
Li	1.951284	0.297147	0.443315
O	3.649225	-0.423372	-0.191844
C	3.56956	-0.901749	-1.536267
H	4.094101	-0.210379	-2.202572
C	4.960591	-0.331894	0.363431
H	4.81624	0.113274	1.348028
H	5.360454	-1.340085	0.513581
Li	-0.48575	0.610322	0.074272
C	1.074244	1.915787	-0.670689
C	0.498722	1.709192	-2.078561
H	-0.494556	1.237219	-2.105795
H	0.401241	2.65897	-2.640306
H	1.144544	1.055382	-2.677403
C	0.335771	3.084978	0.005233
H	0.828603	3.377982	0.93987
H	0.32675	3.985992	-0.638948
H	-0.710724	2.889479	0.267862
C	2.508108	2.426469	-0.877511
H	3.05488	2.57002	0.066546
H	3.109793	1.754154	-1.497534
H	2.517612	3.409407	-1.385728
O	-2.418424	1.293518	-0.034984
C	-2.878987	2.119764	-1.105755
H	-3.382996	1.480489	-1.841548
C	-3.471761	0.617514	0.668303
H	-4.349201	0.55486	0.017245
C	-3.739758	-1.776842	-1.89167

C	-4.32987	-2.566798	-0.906159
C	-3.539806	-3.019012	0.149628
C	-2.196169	-2.679288	0.231436
C	-1.581516	-1.873958	-0.749851
C	-2.39598	-1.440088	-1.815595
H	-4.332645	-1.422502	-2.72926
H	-5.378566	-2.833705	-0.965238
H	-3.976266	-3.645133	0.921106
H	-1.615197	-3.046152	1.071031
H	-1.952002	-0.82581	-2.595095
C	-0.174616	-1.496488	-0.714869
H	0.203342	-1.014001	-1.613959
C	0.692481	-1.753019	0.329675
H	0.395755	-2.443941	1.105007
H	1.764114	-1.760795	0.15968
C	5.912528	0.51892	-0.459923
H	6.829846	0.684227	0.108571
H	6.19316	0.039643	-1.399822
H	5.466877	1.490543	-0.681793
C	-3.781577	3.25731	-0.65937
H	-4.736156	2.903086	-0.266605
H	-3.287942	3.859904	0.106112
H	-3.995403	3.901357	-1.515295
C	-3.801202	1.28928	1.990242
H	-2.916206	1.342333	2.625814
H	-4.183875	2.301226	1.856146
H	-4.559737	0.704305	2.516665
H	-3.135463	-0.405457	0.832612
H	-1.984671	2.53085	-1.570748
H	2.508272	-0.848357	-1.791753
C	4.085121	-2.320534	-1.6934
H	3.591708	-2.991514	-0.987081
H	3.873747	-2.675459	-2.703715
H	5.164451	-2.385515	-1.539933

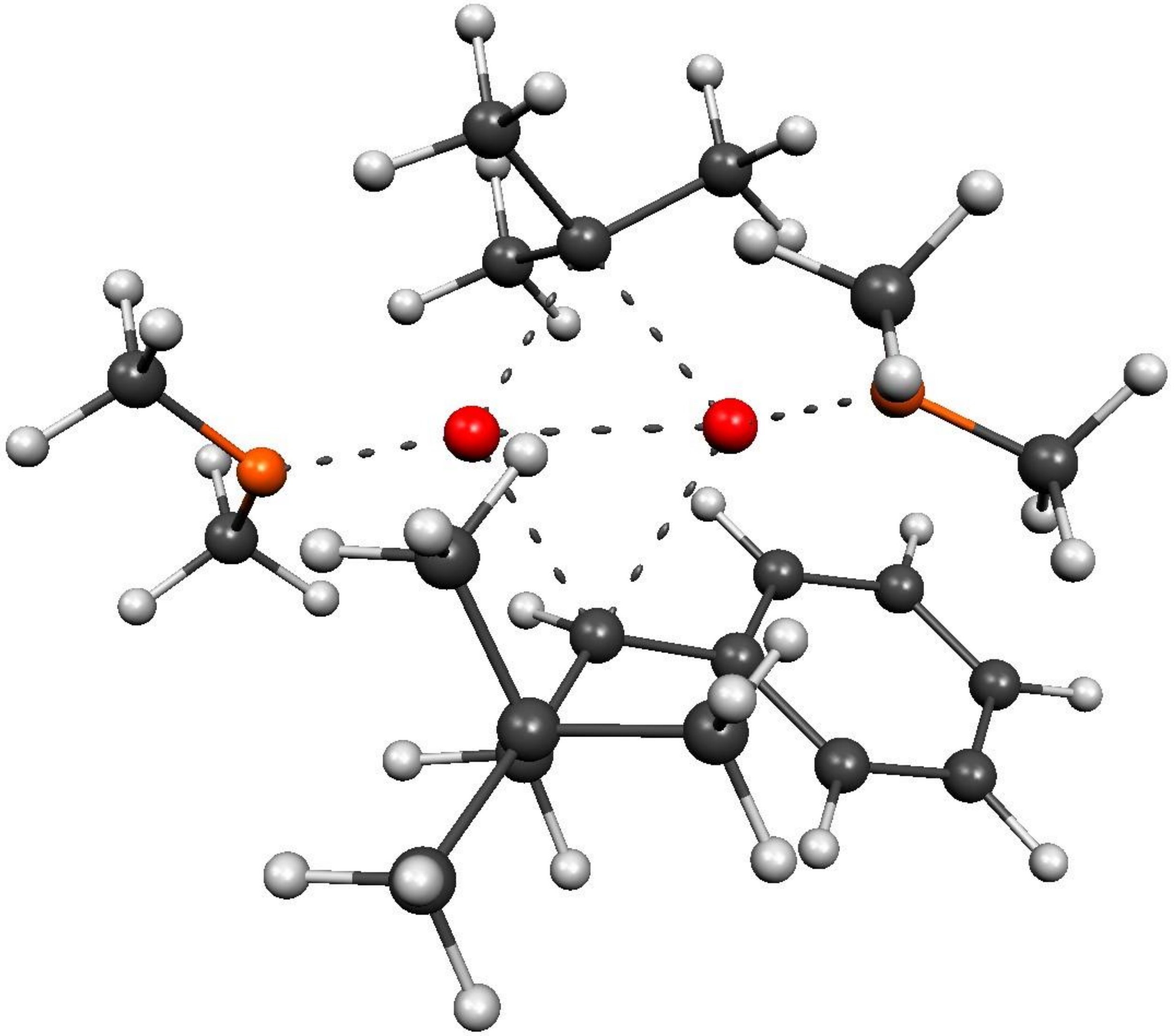

Figure S53: Molekel plot for **9**.

Table S42: Standard orientation of **9** [M062x/6-311+G(d)], T= 298 K, E_{SCF} = – 950.297156093 H, E_{ZPE} = – 949.748199 H, no imaginary frequency.

Atomic symbol	x	y	z
C	-1.147407	2.34288	0.440064
C	-1.747745	2.86634	-0.874033
H	-2.563428	2.237697	-1.263243
H	-2.186655	3.87547	-0.75156
H	-1.002951	2.946302	-1.674761
C	-0.093995	3.368355	0.872922
H	-0.521123	4.387505	0.934384
H	0.328408	3.149258	1.860194
H	0.75288	3.439614	0.174192
C	-2.265202	2.386729	1.486823
H	-3.112506	1.728982	1.242196
H	-1.919908	2.092399	2.484861
H	-2.698521	3.401185	1.589679
Li	0.600013	1.046346	0.243807
O	1.675015	0.851683	1.790023
C	1.081094	0.718985	3.065395
H	1.218126	-0.297736	3.449121
H	1.519258	1.436945	3.76669
H	0.017683	0.927679	2.949817
C	3.074029	0.624392	1.793917
H	3.441075	0.820064	0.788018
H	3.559138	1.297677	2.508382
H	3.290212	-0.41536	2.062307
Li	-1.600384	0.388692	-0.199924
C	-0.4999	-2.636449	0.67342
C	-1.54017	-1.778553	1.40227

H	-2.48426	-1.757248	0.848611
H	-1.749521	-2.179489	2.3985
H	-1.18084	-0.751794	1.551426
C	-0.982749	-4.090116	0.67751
H	-0.282491	-4.734963	0.138756
H	-1.076408	-4.476308	1.697123
H	-1.959724	-4.182178	0.192739
C	0.824815	-2.544213	1.433443
H	1.244168	-1.537309	1.356325
H	0.6807	-2.782514	2.492956
H	1.559503	-3.250196	1.036163
O	-3.292231	-0.20978	-0.968835
C	-4.528193	-0.091278	-0.285075
H	-5.21389	-0.880523	-0.607892
H	-4.331301	-0.19883	0.78044
H	-4.979112	0.888189	-0.475002
C	-3.45994	-0.208756	-2.377612
H	-3.918974	0.728422	-2.708428
H	-2.475604	-0.311967	-2.830422
H	-4.088904	-1.051046	-2.680248
C	3.064703	1.34847	-2.005251
C	4.132404	0.482751	-1.780409
C	3.846465	-0.809043	-1.334978
C	2.544674	-1.223156	-1.09788
C	1.430655	-0.373008	-1.32724
C	1.755223	0.935833	-1.788967
H	3.248307	2.355284	-2.367455
H	5.15336	0.799123	-1.957114
H	4.658949	-1.509857	-1.166521
H	2.376698	-2.237831	-0.760402
H	0.941662	1.621433	-2.027776
C	0.050944	-0.725969	-1.058722
H	-0.570436	-0.275546	-1.846803
C	-0.337523	-2.17722	-0.801759
H	0.359251	-2.881403	-1.285106
H	-1.307083	-2.363294	-1.284341

Table S43: Standard orientation of **9** [M062x/6-311+G(d)], T= 193 K, E_{SCF} = – 950.297173000 H, E_{ZPE} = – 949.748174 H, no imaginary frequency.

Atomic symbol	x	y	z
C	1.161306	-2.337046	0.4334
C	1.789354	-2.836809	-0.8772
H	2.613542	-2.201727	-1.237259
H	2.22557	-3.848132	-0.764042
H	1.062185	-2.901112	-1.695492
C	0.104639	-3.374397	0.828731
H	0.535789	-4.392219	0.883483
H	-0.338953	-3.172413	1.810367
H	-0.727453	-3.439205	0.111879
C	2.258559	-2.393527	1.500831
H	3.104486	-1.723335	1.287813
H	1.891129	-2.122408	2.497388
H	2.698214	-3.406395	1.592019
Li	-0.592512	-1.045969	0.24175
O	-1.684291	-0.877448	1.780621
C	-1.105333	-0.753393	3.063778
H	-1.242086	0.262228	3.450503
H	-1.555995	-1.472292	3.756068
H	-0.041603	-0.967004	2.961119
C	-3.083163	-0.647977	1.770267
H	-3.440313	-0.841996	0.760534
H	-3.576284	-1.321681	2.478792
H	-3.300669	0.391651	2.03784
Li	1.607399	-0.375487	-0.192171
C	0.499084	2.622011	0.707785
C	1.530954	1.752444	1.435332
H	2.480092	1.732725	0.889909
H	1.733661	2.143239	2.436938
H	1.166209	0.725979	1.572632
C	0.986489	4.073901	0.730395
H	0.292113	4.726554	0.193451
H	1.074135	4.449225	1.754574
H	1.967235	4.167646	0.253591
C	-0.831213	2.526307	1.457731
H	-1.252383	1.52114	1.369175
H	-0.693947	2.754683	2.520358
H	-1.561753	3.237244	1.061677
O	3.287559	0.25631	-0.973366
C	4.529808	0.032336	-0.327766
H	5.261631	0.778358	-0.651617
H	4.368898	0.128632	0.744913

H	4.904463	-0.971921	-0.551697
C	3.4199	0.299131	-2.385762
H	3.772411	-0.66429	-2.768082
H	2.440502	0.52366	-2.803999
H	4.124594	1.08555	-2.671177
C	-3.055133	-1.334388	-2.024575
C	-4.123991	-0.469748	-1.800634
C	-3.840793	0.818791	-1.344028
C	-2.540515	1.231102	-1.095737
C	-1.425527	0.382035	-1.324175
C	-1.74716	-0.923671	-1.796719
H	-3.236808	-2.338653	-2.394776
H	-5.143876	-0.784503	-1.986251
H	-4.654376	1.518441	-1.175991
H	-2.373882	2.243712	-0.751221
H	-0.931361	-1.607634	-2.033121
C	-0.047195	0.73238	-1.047006
H	0.577141	0.284219	-1.833559
C	0.34337	2.179687	-0.773394
H	-0.34948	2.890818	-1.252192
H	1.315774	2.369681	-1.248864

Table S44: Standard orientation of **9** [M062x/6-311+G(d),scrf=(pcm,diethyl ether)], T= 298 K, E_{SCF} = – 950.305131709 H, E_{ZPE} = – 949.756412 H, no imaginary frequency.

Atomic symbol	x	y	z
C	1.12438	-2.379133	0.446524
C	1.708413	-2.928849	-0.86268
H	2.536591	-2.321837	-1.257281
H	2.12227	-3.948599	-0.728991
H	0.959775	-2.998765	-1.660785
C	0.050448	-3.375043	0.896181
H	0.45959	-4.402018	0.974254
H	-0.366974	-3.130875	1.879924
H	-0.796833	-3.441442	0.197884
C	2.24361	-2.440819	1.491044
H	3.11157	-1.818171	1.230911
H	1.910694	-2.118887	2.484725
H	2.641044	-3.469716	1.608212
Li	-0.605343	-1.03605	0.277732
O	-1.676612	-0.807785	1.823031
C	-1.077638	-0.656208	3.096492
H	-1.197584	0.370684	3.456858
H	-1.527239	-1.350342	3.813368
H	-0.017775	-0.884488	2.985164
C	-3.075006	-0.572934	1.830376
H	-3.44563	-0.774736	0.826773
H	-3.561774	-1.236938	2.551464
H	-3.284817	0.469525	2.092393
Li	1.617993	-0.398866	-0.165151
C	0.49543	2.671329	0.633031
C	1.540842	1.839897	1.383149
H	2.486647	1.809459	0.83311
H	1.743538	2.266403	2.370005
H	1.187692	0.815202	1.55662
C	0.965192	4.129115	0.604513
H	0.258674	4.755507	0.051869
H	1.05469	4.537188	1.615918
H	1.941902	4.218317	0.118535
C	-0.831268	2.584862	1.390213
H	-1.240364	1.572155	1.336553
H	-0.692444	2.847371	2.444542
H	-1.570927	3.274925	0.974087
O	3.301166	0.16612	-0.947385
C	4.53179	0.119882	-0.241053
H	5.166975	0.959146	-0.537936
H	4.31007	0.193281	0.822449
H	5.051236	-0.822193	-0.441613
C	3.495933	0.16286	-2.354357
H	4.01176	-0.750348	-2.666149
H	2.516461	0.208096	-2.826847
H	4.084482	1.0347	-2.652492
C	-3.036663	-1.380435	-2.004721
C	-4.112204	-0.518656	-1.795204
C	-3.834823	0.781099	-1.363947
C	-2.536792	1.206502	-1.126206
C	-1.41347	0.358939	-1.33769
C	-1.730271	-0.957888	-1.789565
H	-3.212479	-2.393604	-2.353692
H	-5.13091	-0.844761	-1.968162
H	-4.652494	1.478213	-1.203878

H	-2.377002	2.224705	-0.795017
H	-0.911724	-1.642865	-2.010874
C	-0.043919	0.724086	-1.06519
H	0.601432	0.241706	-1.812127
C	0.34227	2.178943	-0.83206
H	-0.355521	2.871364	-1.331546
H	1.313488	2.355402	-1.314242

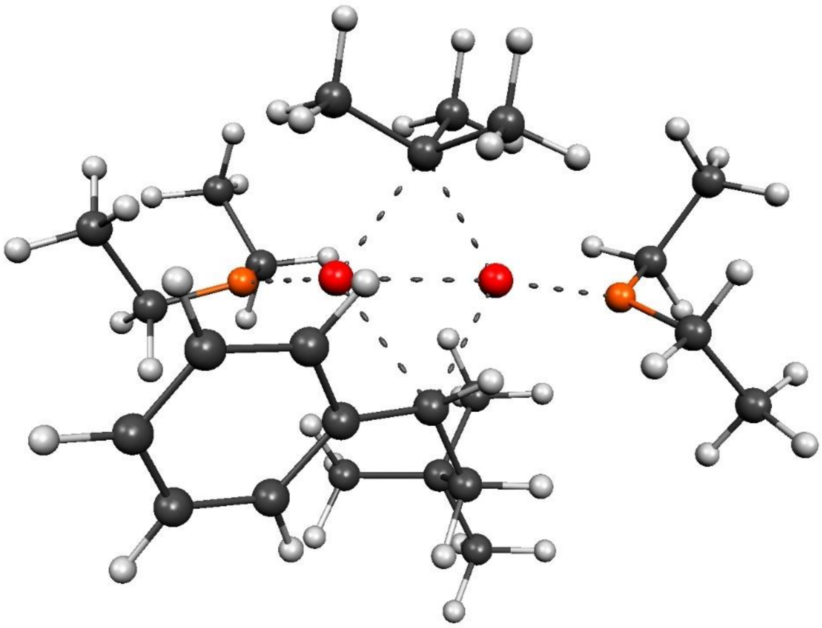

Figure S54: Molekel plot for **27**.

Table S45: Standard orientation of **27** [M062x/6-311+G(d)], T= 298 K, E_{SCF} = – 1107.51996226 H, E_{ZPE} = – 1106.855917 H, no imaginary frequency.

Atomic symbol	x	y	z
C	0.321917	2.375306	0.576516
C	1.166966	2.539038	1.849925
H	2.172122	2.095818	1.77759
H	1.334706	3.60552	2.095918
H	0.68824	2.090246	2.728775
C	-1.005828	3.089355	0.854281
H	-0.846186	4.149241	1.131241
H	-1.674891	3.092022	-0.013557
H	-1.567186	2.640567	1.685857
C	1.020685	3.16023	-0.537482
H	2.019685	2.769313	-0.779722
H	0.44619	3.153771	-1.471883
H	1.172147	4.224247	-0.266394
Li	-0.983355	0.648536	0.282719
O	-2.209846	0.736715	-1.151054
C	-1.794124	1.095771	-2.463487
H	-2.021999	0.267745	-3.145608
H	-0.707149	1.18709	-2.403145
C	-3.592763	0.399641	-0.990872
H	-3.621639	-0.539295	-0.434887
H	-4.030928	0.222656	-1.978934
Li	1.341631	0.545599	0.352343
C	0.788427	-2.378059	-1.466761
C	1.559877	-1.171967	-2.008894
H	2.591147	-1.172485	-1.646223
H	1.584312	-1.186825	-3.102975
H	1.085829	-0.227067	-1.714706
C	1.471013	-3.650209	-1.979972
H	0.961095	-4.544142	-1.609526
H	1.466457	-3.691258	-3.073565
H	2.513099	-3.69988	-1.647558
C	-0.644171	-2.327999	-2.003632
H	-1.200159	-1.494335	-1.565657
H	-0.642177	-2.208564	-3.092582
H	-1.184783	-3.251265	-1.778643
O	3.235856	0.241422	0.356565
C	4.120522	0.837914	-0.591722

H	4.785661	0.070044	-0.999303
H	3.47549	1.169093	-1.407414
C	3.845904	-0.346282	1.507182
H	4.286218	0.440296	2.128886
H	3.017784	-0.783818	2.067562
C	-2.967578	-0.350212	2.710278
C	-3.863317	-1.337327	2.307847
C	-3.395636	-2.336586	1.452195
C	-2.092156	-2.334865	0.978604
C	-1.154482	-1.338603	1.358466
C	-1.653346	-0.35255	2.258382
H	-3.288362	0.428235	3.396366
H	-4.886399	-1.34126	2.664428
H	-4.064927	-3.134312	1.143129
H	-1.778543	-3.134666	0.320449
H	-0.965483	0.405751	2.631618
C	0.196139	-1.232162	0.840398
H	0.846275	-0.899322	1.665209
C	0.81661	-2.402773	0.086657
H	0.382587	-3.365673	0.402232
H	1.877474	-2.467645	0.367564
C	4.904457	2.005152	-0.019595
H	5.632117	1.684422	0.728858
H	5.45412	2.504328	-0.820024
H	4.230631	2.732787	0.437961
C	4.866595	-1.415859	1.16124
H	5.778815	-0.998975	0.730081
H	5.148625	-1.953943	2.068034
H	4.442615	-2.13506	0.456447
C	-4.331416	1.487501	-0.234688
H	-4.318952	2.435851	-0.775763
H	-5.369765	1.18959	-0.070665
H	-3.868116	1.640379	0.74194
C	-2.41032	2.393979	-2.954578
H	-2.007076	2.643151	-3.938782
H	-3.495402	2.315819	-3.051751
H	-2.180074	3.213717	-2.271873

Table S46: Standard orientation of **27** [M062x/6-311+G(d)], T= 193 K, E_{SCF} = – 1107.51996226 H, E_{ZPE} = – 1106.855917 H, no imaginary frequency.

Atomic symbol	x	y	z
C	0.321917	2.375306	0.576516
C	1.166966	2.539038	1.849925
H	2.172122	2.095818	1.77759
H	1.334706	3.605521	2.095918
H	0.68824	2.090246	2.728775
C	-1.005827	3.089355	0.854281
H	-0.846186	4.149241	1.13124
H	-1.674891	3.092022	-0.013557
H	-1.567186	2.640567	1.685857
C	1.020686	3.16023	-0.537482
H	2.019685	2.769313	-0.779722
H	0.44619	3.153771	-1.471883
H	1.172147	4.224247	-0.266394
Li	-0.983355	0.648536	0.282719
O	-2.209846	0.736715	-1.151054
C	-1.794124	1.09577	-2.463486
H	-2.021999	0.267745	-3.145608
H	-0.707149	1.18709	-2.403145
C	-3.592763	0.399641	-0.990872
H	-3.621639	-0.539295	-0.434887
H	-4.030929	0.222656	-1.978934
Li	1.341631	0.545599	0.352343
C	0.788427	-2.378059	-1.466761
C	1.559877	-1.171967	-2.008894
H	2.591147	-1.172485	-1.646223
H	1.584312	-1.186825	-3.102975
H	1.085829	-0.227067	-1.714706
C	1.471013	-3.650209	-1.979972
H	0.961095	-4.544142	-1.609526
H	1.466457	-3.691258	-3.073565
H	2.513099	-3.69988	-1.647558
C	-0.644171	-2.327999	-2.003632
H	-1.20016	-1.494335	-1.565657
H	-0.642178	-2.208564	-3.092582
H	-1.184782	-3.251265	-1.778643
O	3.235856	0.241422	0.356565
C	4.120522	0.837914	-0.591722
H	4.785661	0.070044	-0.999303
H	3.475491	1.169093	-1.407414
C	3.845904	-0.346282	1.507182

H	4.286218	0.440296	2.128886
H	3.017784	-0.783818	2.067562
C	-2.967578	-0.350212	2.710278
C	-3.863316	-1.337327	2.307848
C	-3.395636	-2.336586	1.452195
C	-2.092156	-2.334865	0.978604
C	-1.154482	-1.338603	1.358466
C	-1.653346	-0.352549	2.258382
H	-3.288362	0.428235	3.396366
H	-4.886399	-1.34126	2.664429
H	-4.064927	-3.134312	1.14313
H	-1.778543	-3.134666	0.320449
H	-0.965483	0.405751	2.631618
C	0.196139	-1.232162	0.840398
H	0.846276	-0.899322	1.665209
C	0.81661	-2.402773	0.086657
H	0.382587	-3.365673	0.402232
H	1.877474	-2.467644	0.367564
C	4.904457	2.005152	-0.019595
H	5.632117	1.684422	0.728858
H	5.45412	2.504328	-0.820024
H	4.230631	2.732787	0.437961
C	4.866596	-1.415859	1.16124
H	5.778815	-0.998975	0.730081
H	5.148625	-1.953942	2.068034
H	4.442615	-2.13506	0.456447
C	-4.331417	1.487501	-0.234688
H	-4.318953	2.435851	-0.775763
H	-5.369765	1.18959	-0.070664
H	-3.868116	1.640379	0.74194
C	-2.410321	2.393979	-2.954579
H	-2.007076	2.643151	-3.938782
H	-3.495403	2.315819	-3.051751
H	-2.180074	3.213717	-2.271874

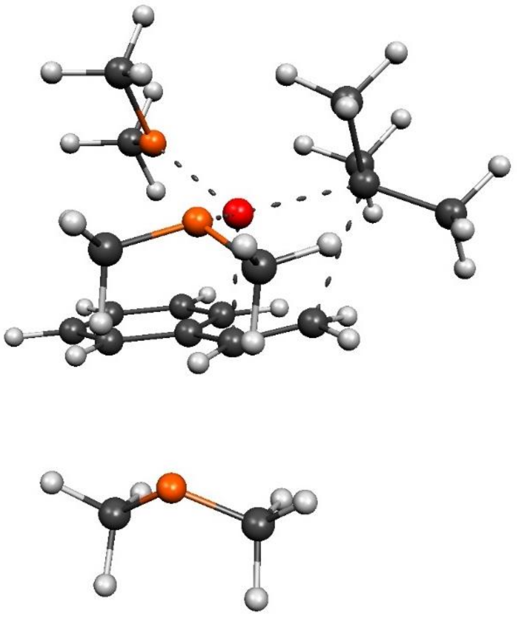

Figure S55: Molekel plot of **15**.

Table S47: Standard orientation of **15** [M062x/6-311+G(d),scrf=(pcm,water)], T= 298 K, E_{SCF} = – 939.913271336 H, E_{ZPE} = – 939.407926 H, one imaginary frequency.

Atomic symbol	x	y	z
C	1.868015	-1.853539	-1.225249
C	1.552899	-2.869667	-2.299409
H	0.84561	-3.632569	-1.950779
H	1.118316	-2.41081	-3.195934
H	2.460963	-3.407085	-2.631233
C	2.473643	-2.584656	-0.026749
H	3.379012	-3.148314	-0.328684
H	2.800752	-1.90564	0.769238
H	1.791842	-3.313802	0.42414
C	2.884499	-0.84773	-1.742526
H	3.835898	-1.338748	-2.028743
H	2.527711	-0.319697	-2.634491

H	3.138622	-0.086688	-0.993419
Li	0.824396	-0.7382	0.276591
O	-0.289537	-1.779184	1.485157
O	1.788829	0.294398	1.632093
O	-3.894361	-0.824229	0.148793
C	-1.029733	-2.831969	0.88525
H	-1.301759	-3.579719	1.636383
H	-1.938695	-2.433104	0.423898
H	-0.397479	-3.284966	0.123453
C	-1.062816	-1.079129	2.449459
H	-2.010559	-0.751617	2.009927
H	-1.266982	-1.724477	3.309134
H	-0.486814	-0.211133	2.767068
C	2.386347	1.571216	1.457306
H	2.011199	1.989791	0.526015
H	2.116749	2.22965	2.288572
H	3.475727	1.473486	1.411297
C	2.221308	-0.33412	2.828914
H	1.733821	-1.305873	2.886372
H	3.3075	-0.468354	2.81807
H	1.941926	0.271988	3.69619
C	-4.010449	-1.316868	-1.169198
H	-3.762736	-2.377951	-1.150142
H	-5.033323	-1.194427	-1.543671
H	-3.317579	-0.794505	-1.839038
C	-4.210502	0.549188	0.217715
H	-5.254185	0.725081	-0.067197
H	-4.060779	0.871941	1.247959
H	-3.559225	1.133925	-0.443713
C	-0.37919	1.619126	-0.731644
C	-0.864998	2.427371	0.317082
C	-0.454317	3.744608	0.471982
C	0.457934	4.311902	-0.418828
C	0.943687	3.53306	-1.469495
C	0.538645	2.212788	-1.623625
H	-1.563692	1.99566	1.02888
H	-0.842711	4.332449	1.297408
H	0.782821	5.338815	-0.297415
H	1.652539	3.957448	-2.173264
H	0.946326	1.631246	-2.44338
C	-0.806006	0.231417	-0.835019
H	-1.568495	-0.087701	-0.128801
C	-0.249417	-0.7124	-1.668273
H	-0.719323	-1.686127	-1.752761
H	0.347056	-0.425047	-2.525606

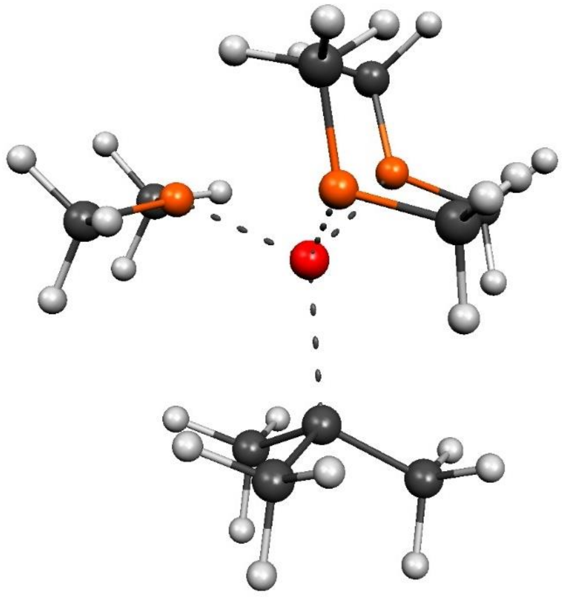

Figure S56: Molekel plot of **7**.

Table S48: Standard orientation of **7** [M062x/6-311+G(d),scrf=(pcm,water)], T= 298 K, E_{SCF} = – 630.339068091 H, E_{ZPE} = – 629.971266 H, no imaginary frequency.

Atomic symbol	x	y	z
C	2.105143	-0.242566	0.158296
C	2.654368	0.008193	-1.240999
H	2.393595	-0.78811	-1.950588

H	2.288019	0.948919	-1.673633
H	3.769594	0.07569	-1.248845
C	2.737626	-1.526661	0.672258
H	3.853563	-1.486321	0.64361
H	2.468906	-1.740073	1.715177
H	2.44871	-2.408055	0.08325
C	2.602444	0.897719	1.03896
H	2.251089	1.877424	0.68809
H	2.27863	0.799611	2.083747
H	3.718167	0.959739	1.061586
Li	0.01491	0.013668	-0.013146
O	-0.670438	1.828072	-0.032447
O	-0.765686	-0.645991	-1.69329
O	-1.25481	-0.779906	1.230803
C	-0.278025	2.629561	-1.133324
H	-0.995432	3.441007	-1.28968
H	0.717591	3.051991	-0.963207
H	-0.252079	1.985212	-2.011318
C	-0.710879	2.556681	1.182454
H	-0.941898	1.85104	1.979349
H	0.257831	3.024003	1.382917
H	-1.484831	3.328935	1.137066
C	-0.455405	-2.027471	-1.77319
H	0.602396	-2.133313	-1.537569
H	-1.050122	-2.592531	-1.046455
H	-0.658237	-2.40793	-2.779071
C	-2.140696	-0.397695	-1.922625
H	-2.755489	-0.97136	-1.221034
H	-2.313252	0.666379	-1.767702
H	-2.413075	-0.672459	-2.946303
C	-0.879479	-1.958602	1.922519
H	0.138726	-2.196989	1.617447
H	-0.91288	-1.795347	3.00444
H	-1.54987	-2.784015	1.661883
C	-2.57443	-0.363257	1.526618
H	-2.671988	-0.124116	2.590732
H	-2.77656	0.525617	0.930324
H	-3.292366	-1.148761	1.268903

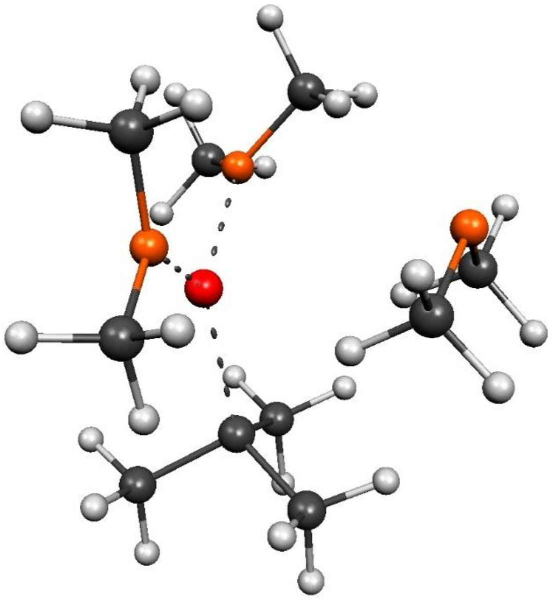

Figure S57: Molekel plot of **17.**

Table S49: Standard orientation of **17** [M062x/6-311+G(d),scrf=(pcm,water)], T= 298 K, E_{SCF} = – 630.328786902 H, E_{ZPE} = – 629.961787 H, one imaginary frequency.

Atomic symbol	x	y	z
C	2.172854	0.186736	-0.09006
C	2.544992	1.481716	0.625468
H	2.349706	2.373217	0.015105
H	1.996796	1.615295	1.567634
H	3.629024	1.517869	0.889201
C	3.043427	0.100549	-1.340689
H	4.131999	0.134204	-1.096187
H	2.885912	-0.830125	-1.901951
H	2.861862	0.927979	-2.03927
C	2.581951	-0.960806	0.822531

H	2.059759	-0.93867	1.787547
H	2.387228	-1.947947	0.381228
H	3.672394	-0.934944	1.05689
Li	0.186796	0.38687	-0.78581
O	-1.492944	-1.019677	2.152145
O	-1.103065	1.783469	-0.43191
O	-0.809065	-1.03014	-1.602011
C	-0.64539	-0.015653	2.669355
H	0.005164	-0.42027	3.454497
H	-0.016042	0.411406	1.878135
H	-1.277335	0.762552	3.095765
C	-0.752393	-2.096603	1.617685
H	-1.462817	-2.803208	1.188783
H	-0.062621	-1.747476	0.841963
H	-0.17249	-2.598257	2.402477
C	-0.782525	3.126582	-0.753995
H	0.128326	3.114377	-1.35073
H	-1.591975	3.583201	-1.331041
H	-0.6136	3.706953	0.157937
C	-2.295162	1.674017	0.328679
H	-3.152693	2.014122	-0.260465
H	-2.417073	0.626685	0.602081
H	-2.221447	2.278466	1.237996
C	-0.164932	-2.274542	-1.82232
H	0.859436	-2.17209	-1.465961
H	-0.670679	-3.072036	-1.269468
H	-0.165918	-2.517947	-2.888885
C	-2.18046	-1.050722	-1.956839
H	-2.733426	-1.728958	-1.299328
H	-2.562459	-0.037669	-1.846318
H	-2.300999	-1.374193	-2.994887

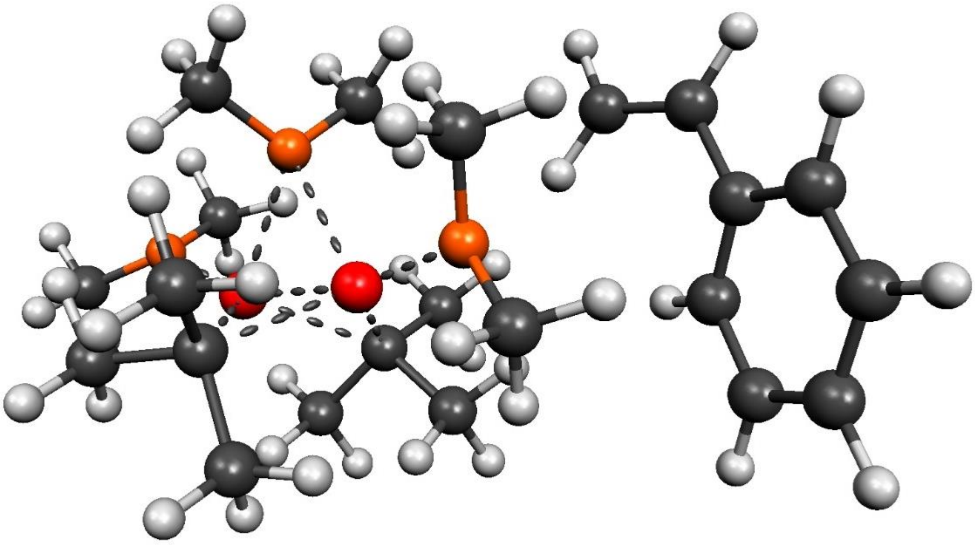

Figure S58: Molekel plot of **20.**

Table S50: Standard orientation of **20** [M062x/6-311+G(d),scrf=(pcm,water)], T= 298 K, E_{SCF} = – 1105.24654622 H, E_{ZPE} = – 1104.618169 H, no imaginary frequency.

Atomic symbol	x	y	z
O	1.707459	0.753364	-1.478379
O	4.312019	0.778971	0.048178
C	1.092472	0.756903	1.7166
C	1.164855	2.053346	-1.622112
H	1.948183	2.769488	-1.895909
H	0.734012	2.325126	-0.660481
C	5.621241	0.344561	0.368885
H	6.355437	0.873049	-0.247894
H	5.676206	-0.722162	0.168073
C	2.255586	0.413607	2.659998
H	2.000701	0.599743	3.721945
H	3.154817	1.014036	2.461583
H	2.553731	-0.643804	2.604399
C	4.17085	2.181973	0.183292
H	3.113059	2.414346	0.078497
H	4.751974	2.69819	-0.588749
C	-0.128297	-0.00423	2.253305
H	0.01052	-1.092884	2.241642
H	-1.040995	0.21586	1.682024
H	-0.354278	0.26083	3.305935

C	0.78492	2.240085	1.923245
H	0.597989	2.480126	2.989577
H	-0.115851	2.563636	1.383841
H	1.599907	2.903389	1.604408
Li	2.574326	-0.091512	0.305043
C	2.42844	0.343849	-2.627427
H	2.890738	-0.611496	-2.395479
H	3.204645	1.07905	-2.866305
O	-1.240366	-0.809322	-0.771542
C	1.934426	-2.192198	-0.130442
C	-2.06226	-1.82881	-0.218287
H	-1.917887	-1.817152	0.860294
H	-1.772939	-2.805944	-0.61923
C	1.38558	-3.048444	1.017686
H	1.701293	-4.107404	0.925222
H	0.287797	-3.061943	1.045157
H	1.72132	-2.706062	2.003101
C	-1.420142	-0.691838	-2.170138
H	-0.767165	0.107454	-2.519009
H	-2.459487	-0.431209	-2.393564
C	3.455901	-2.388061	-0.09892
H	3.904006	-2.03189	0.841679
H	3.978875	-1.877747	-0.921195
H	3.737538	-3.457292	-0.175956
C	1.431477	-2.860669	-1.415561
H	1.767185	-3.91503	-1.489159
H	1.765458	-2.368988	-2.337052
H	0.335734	-2.892345	-1.457941
Li	0.520762	-0.512606	-0.028761
H	-1.153684	-1.627794	-2.67229
H	-3.112822	-1.628322	-0.446331
H	1.757209	0.231367	-3.486471
H	0.393721	2.065032	-2.400884
H	4.507587	2.512008	1.171501
H	5.834553	0.531959	1.426603
C	-5.774712	-0.7516	0.000342
C	-5.333195	-0.944072	1.306231
C	-4.311223	-0.144402	1.813845
C	-3.727784	0.832259	1.01801
C	-4.146107	1.019759	-0.304145
C	-5.188219	0.226901	-0.795899
H	-6.577423	-1.362057	-0.398081
H	-5.791214	-1.703711	1.929499
H	-3.970002	-0.277265	2.834733
H	-2.953649	1.470247	1.430115
H	-5.535344	0.375178	-1.81427
C	-3.510566	2.026274	-1.174132
H	-4.133602	2.43935	-1.964882
C	-2.245103	2.425029	-1.061184
H	-1.581638	1.99631	-0.315231
H	-1.833207	3.183656	-1.717522

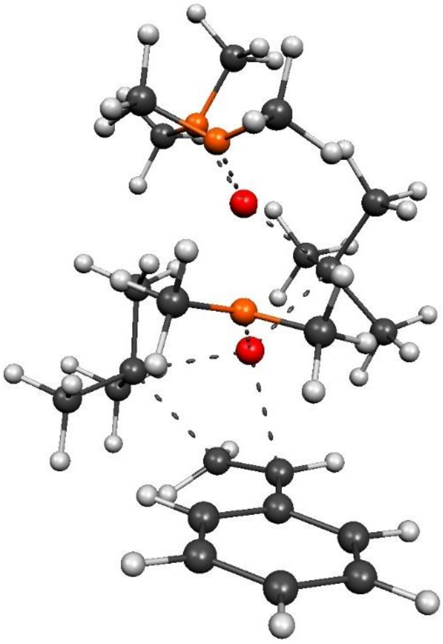

Figure S59: Molekel plot of **21.**

Table S51: Standard orientation of **21** [M062x/6-311+G(d),scrf=(pcm,water)], T= 298 K, E_{SCF} = – 1104.97105688 H, E_{ZPE} = – 1104.339012 H, one imaginary frequency.

Atomic symbol	x	y	z
O	-2.130446	-1.876071	-0.891902
O	-4.045937	0.066481	-0.322664
C	-1.271629	0.194459	2.101448
C	-1.806183	-3.169066	-0.417211
H	-2.717023	-3.772509	-0.297402
H	-1.314221	-3.046618	0.547655
C	-4.68862	1.322789	-0.485557
H	-5.476487	1.240851	-1.24444
H	-3.93738	2.041066	-0.813537
C	-2.306597	1.313452	2.300987
H	-2.685662	1.3403	3.344742
H	-3.200101	1.20958	1.665417
H	-1.880497	2.30417	2.088334
C	-4.95541	-0.924914	0.123919
H	-4.414376	-1.872211	0.169531
H	-5.794776	-1.013999	-0.577796
C	-0.213409	0.430398	3.181941
H	0.239993	1.42686	3.104251
H	0.605704	-0.302505	3.16033
H	-0.662759	0.373411	4.196373
C	-1.95943	-1.125022	2.495267
H	-2.428807	-1.061419	3.499599
H	-1.243856	-1.958546	2.532719
H	-2.782606	-1.447295	1.825128
Li	-2.117975	-0.299924	0.26434
C	-2.704074	-1.890624	-2.185923
H	-2.017129	-2.368731	-2.896148
H	-2.8807	-0.854357	-2.477288
O	0.705468	-1.184021	-0.442886
C	0.084572	2.294268	-1.103744
C	1.446584	-2.047246	0.410156
H	1.333005	-1.672741	1.430279
H	2.507853	-2.045255	0.136715
C	-0.29707	3.748456	-0.894596
H	-0.913237	4.12921	-1.731009
H	0.585957	4.398269	-0.832214
H	-0.875607	3.889865	0.02888
C	0.884129	-1.53031	-1.801209
H	0.213627	-0.909793	-2.398764
H	0.63214	-2.589132	-1.958738
C	-1.217315	1.500858	-1.212124
H	-1.84518	1.667273	-0.326215
H	-1.052454	0.420833	-1.361726
H	-1.814673	1.830468	-2.090813
C	0.834182	2.157239	-2.418683

H	0.229065	2.539481	-3.264144
H	1.092495	1.117539	-2.653563
H	1.769354	2.733475	-2.422952
Li	0.38155	0.604008	0.476907
H	1.923475	-1.357969	-2.107317
H	1.047632	-3.069285	0.341284
H	-3.657358	-2.43756	-2.180332
H	-1.129845	-3.676457	-1.116502
H	-5.336986	-0.67396	1.122336
H	-5.127652	1.657002	0.462777
C	1.731147	2.384757	0.42534
H	2.249443	2.835438	-0.418621
H	1.159399	3.092923	1.02142
C	2.353129	1.345221	1.120821
H	2.138106	1.223396	2.178048
C	3.378076	0.486239	0.573947
C	4.152345	-0.337703	1.425297
C	3.640414	0.391672	-0.810926
C	5.108582	-1.210296	0.925493
H	3.981767	-0.280559	2.499076
C	4.605073	-0.481583	-1.308503
H	3.062826	0.999868	-1.501872
C	5.342198	-1.300614	-0.451983
H	5.681762	-1.8275	1.613301
H	4.778309	-0.527492	-2.381847
H	6.089905	-1.983329	-0.844008

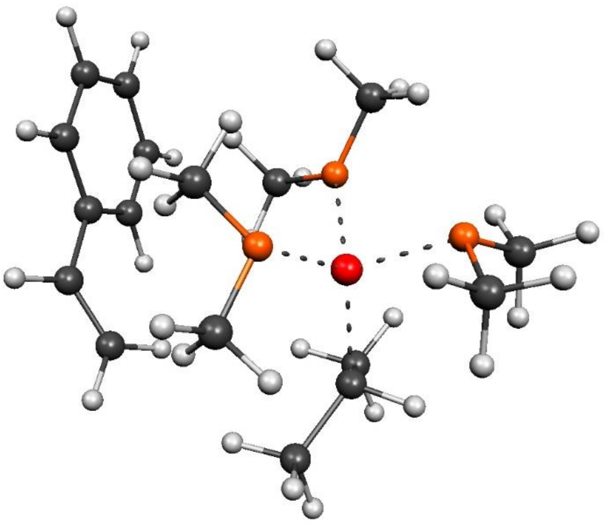

Figure S60: Molekel plot of **1+29.**

Table S52: Standard orientation of **1+29** [M062x/6-311+G(d)], T= 298 K, E_{SCF} = – 900.602711200 H, E_{ZPE} = – 900.126047 H, no imaginary frequency.

Atomic symbol	x	y	z
C	-2.217872	1.362185	-1.293223
C	-2.053379	2.872905	-1.107802
H	-2.653577	3.263037	-0.276017
H	-1.011719	3.144607	-0.887376
H	-2.334109	3.466674	-1.998809
C	-1.511294	0.966033	-2.597686
H	-0.428615	1.155849	-2.540538
H	-1.61725	-0.102891	-2.832775
H	-1.857851	1.52273	-3.489565
Li	-1.883519	-0.136766	0.123596
O	-1.372951	0.351365	1.975207
O	-0.640018	-1.561288	-0.274711
O	-3.45989	-1.313209	0.274536
C	-1.508418	1.760986	2.08814
H	-1.346896	2.068421	3.127684
H	-0.800446	2.267506	1.425713
H	-2.517412	2.017232	1.771724
C	-0.07966	-0.083152	2.346952
H	0.67783	0.342513	1.678979
H	0.139832	0.214835	3.37854
H	-0.061365	-1.169262	2.271702
C	0.611961	-1.150202	-0.793487
H	0.567265	-0.066499	-0.898595
H	1.421562	-1.425591	-0.108028
H	0.793561	-1.603862	-1.773819
C	-0.708058	-2.940947	0.000661

H	-1.687868	-3.136266	0.434693
H	-0.583731	-3.525277	-0.918411
H	0.074496	-3.233355	0.711305
C	-3.847977	-1.70296	-1.031802
H	-2.99648	-2.210273	-1.484531
H	-4.703398	-2.385747	-0.984234
H	-4.100956	-0.827244	-1.635886
C	-4.468776	-0.570128	0.933869
H	-5.382028	-1.167124	1.031383
H	-4.0873	-0.32147	1.923412
H	-4.685628	0.349563	0.379683
C	3.194236	0.814591	0.165122
C	3.620995	-0.044318	1.182812
C	4.192318	-1.277386	0.885298
C	4.358978	-1.665476	-0.440312
C	3.961612	-0.806942	-1.463749
C	3.388523	0.421994	-1.164588
H	3.492452	0.255717	2.218833
H	4.511078	-1.932665	1.688348
H	4.808488	-2.623395	-0.676054
H	4.1062	-1.094198	-2.499392
H	3.096754	1.090058	-1.967995
C	2.527913	2.081645	0.516051
H	2.766655	2.483706	1.498838
C	1.642837	2.72239	-0.246485
H	1.185804	3.647982	0.083435
H	1.324168	2.349894	-1.21528
H	-3.299714	1.212844	-1.48664

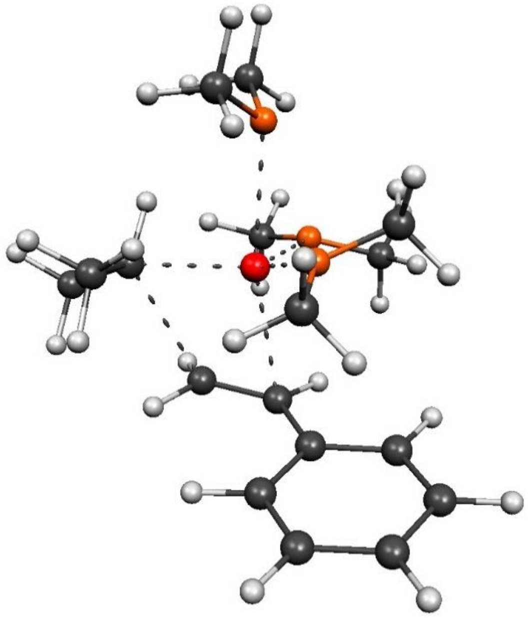

Figure S61: Molekel plot of **22.**

Table S53: Standard orientation of **22** [M062x/6-311+G(d)], T= 298 K, E_{SCF} = – 900.597268789 H, E_{ZPE} = – 900.119702 H, one imaginary frequency.

Atomic symbol	x	y	z
C	-1.650369	1.944398	-0.343118
C	-2.183065	3.188529	0.335925
H	-2.718486	2.965339	1.267387
H	-1.370426	3.878392	0.604427
H	-2.873246	3.768417	-0.296646
C	-1.013117	2.293856	-1.68016
H	-0.095741	2.882573	-1.537625
H	-0.731313	1.407864	-2.259026
H	-1.663004	2.902769	-2.333122
Li	-0.785413	0.048199	0.116127
O	-1.658107	-0.891523	1.673944
O	0.273779	-0.993713	-1.18419
O	-2.526225	-1.156005	-1.10225
C	-2.524105	-0.05146	2.414477
H	-3.345727	-0.634347	2.847124
H	-1.974841	0.448707	3.22001
H	-2.917535	0.696493	1.728176
C	-1.101463	-1.919215	2.46536
H	-0.628313	-1.508412	3.364065

H	-1.875941	-2.634026	2.765267
H	-0.345306	-2.427682	1.868633
C	1.306292	-0.58229	-2.064977
H	1.309566	0.504225	-2.08574
H	2.279472	-0.937094	-1.713279
H	1.109503	-0.973737	-3.070883
C	0.234414	-2.397988	-1.037955
H	-0.637751	-2.639503	-0.433338
H	0.126015	-2.8814	-2.016209
H	1.153851	-2.754605	-0.559364
C	-2.570579	-0.765439	-2.458357
H	-1.549188	-0.7833	-2.834202
H	-3.190114	-1.45903	-3.039407
H	-2.973022	0.249635	-2.558975
C	-3.814891	-1.200058	-0.534918
H	-4.468132	-1.871517	-1.104936
H	-3.712335	-1.573781	0.481833
H	-4.268287	-0.200596	-0.514147
C	2.225881	0.433765	0.869924
C	2.846722	-0.813283	1.076343
C	4.047094	-1.144357	0.46181
C	4.678138	-0.237126	-0.38784
C	4.086918	1.008694	-0.596024
C	2.886714	1.338846	0.017478
H	2.369304	-1.529246	1.741211
H	4.495327	-2.115286	0.647633
H	5.616392	-0.490275	-0.867686
H	4.57076	1.733563	-1.242835
H	2.455244	2.318134	-0.161809
C	0.970303	0.737726	1.540638
H	0.688311	0.044507	2.328643
C	0.149232	1.806248	1.283626
H	-0.625535	2.068816	1.993319
H	0.480795	2.630208	0.661896
H	-2.494367	1.25446	-0.512208

References

[1] T. Kottke, D. Stalke, *J. Appl. Cryst.* **1993**, *26*, 615–619.
[2] Bruker, *Apex4*, Bruker AXS Inc., Madison, Wisconsin, USA **2021**.
[3] O. V. Dolomanov, L. J. Bourhis, R. J. Gildea, J. A. K. Howard, H. Puschmann, *J. Appl. Cryst.* **2009**, *42*, 339–341.
[4] G. M. Sheldrick, *Acta Cryst.* **2015**, *A71*, 3–8.
[5] G. M. Sheldrick, *Acta Cryst.* **2015**, *C71*, 3–8.
[6] R. Dennington, T. Keith, J. Millam, *GaussView 6.0.16*, Semichem Inc., Shawnee Mission, KS, USA **2016**.
[7] M. J. Frisch, G. W. Trucks, H. B. Schlegel, G. E. Scuseria, M. A. Robb, J. R. Cheeseman, G. Scalmani, V. Barone, G. A. Petersson, H. Nakatsuji, X. Li, M. Caricato, A. V. Marenich, J. Bloino, B. G. Janesko, R. Gomperts, B. Mennucci, H. P. Hratchian, J. V. Ortiz, A. F. Izmaylov, J. L. Sonnenberg, D. Williams-Young, F. Ding, F. Lipparini, F. Egidi, J. Goings, B. Peng, A. Petrone, T. Henderson, D. Ranasinghe, V. G. Zakrzewski, J. Gao, N. Rega, G. Zheng, W. Liang, M. Hada, M. Ehara, K. Toyota, R. Fukuda, J. Hasegawa, M. Ishida, T. Nakajima, Y. Honda, O. Kitao, H. Nakai, T. Vreven, K. Throssell, J. A. Montgomery, Jr., J. E. Peralta, F. Ogliaro, M. J. Bearpark, J. J. Heyd, E. N. Brothers, K. N. Kudin, V. N. Staroverov, T. A. Keith, R. Kobayashi, J. Normand, K. Raghavachari, A. P. Rendell, J. C. Burant, S. S. Iyengar, J. Tomasi, M. Cossi, J. M. Millam, M. Klene, C. Adamo, R. Cammi, J. W. Ochterski, R. L. Martin, K. Morokuma, O. Farkas, J. B. Foresman, D. J. Fox, *Gaussian 16*, Gaussian Inc., Wallingford CT, USA **2016**.
[8] P. Flükiger, H. P. Lüthi, S. Portmann, J. Weber, *MOLEKEL 4.3*, Swiss Center for Scientific Computing, Manno, Switzerland **2000**.
[9] a) X. Wei, R. L. K. Taylor, *Chem. Commun.* **1996**, 187–188; b) X. Wei, P. Johnson, R. J. K. Taylor, *J. Chem. Soc., Perkin Trans 1* **2000**, 1109–1116.
[10] L. J. Farrugia, *J. Appl. Cryst.* **2012**, *45*, 849–854.
[11] V. H. Gessner, S. G. Koller, C. Strohmann, A.-M. Hogan, D. F. O'Shea, *Chem. Eur. J.* **2011**, *17*, 2996–3004.
[12] K. Götz, V. H. Gessner, C. Unkelbach, M. Kaupp, C. Strohmann, *Z. Anorg. Allg. Chem.* **2013**, *639*, 2077–2085.
[13] J. Kleinheider, T. Schrimpf, R. Scheel, T. Mairath, A. Hermann, K. Knepper, C. Strohmann, *Chem. Eur. J.* **2024**, *30*, e202304226

Author Contributions

A.S.: data curation, formal analysis, investigation, project administration, validation, writing of the original draft
C.S.: funding acquisition, validation, correction of the original draft
